# Protein length distribution is remarkably uniform across the tree of life

**DOI:** 10.1186/s13059-023-02973-2

**Published:** 2023-06-08

**Authors:** Yannis Nevers, Natasha M. Glover, Christophe Dessimoz, Odile Lecompte

**Affiliations:** 1 Department of Computational Biology, University of Lausanne, Lausanne, Switzerland; 2 Swiss Institute for Bioinformatics, University of Lausanne, Lausanne, Switzerland; 3 Department of Computer Science, University College London, London, UK; 4 Centre for Life’s Origins and Evolution, Department of Genetics, Evolution and Environment, University College London, London, UK; 5 Department of Computer Science, Centre de Recherche en Biomédecine de Strasbourg, ICube, UMR 7357, University of Strasbourg, CNRS, Strasbourg, France

**Keywords:** Genome evolution, Comparative genomics, Protein length, Genome annotation

## Abstract

**Background:**

In every living species, the function of a protein depends on its organization of structural domains, and the length of a protein is a direct reflection of this. Because every species evolved under different evolutionary pressures, the protein length distribution, much like other genomic features, is expected to vary across species but has so far been scarcely studied.

**Results:**

Here we evaluate this diversity by comparing protein length distribution across 2326 species (1688 bacteria, 153 archaea, and 485 eukaryotes). We find that proteins tend to be on average slightly longer in eukaryotes than in bacteria or archaea, but that the variation of length distribution across species is low, especially compared to the variation of other genomic features (genome size, number of proteins, gene length, GC content, isoelectric points of proteins). Moreover, most cases of atypical protein length distribution appear to be due to artifactual gene annotation, suggesting the actual variation of protein length distribution across species is even smaller.

**Conclusions:**

These results open the way for developing a genome annotation quality metric based on protein length distribution to complement conventional quality measures. Overall, our findings show that protein length distribution between living species is more uniform than previously thought. Furthermore, we also provide evidence for a universal selection on protein length, yet its mechanism and fitness effect remain intriguing open questions.

**Supplementary Information:**

The online version contains supplementary material available at 10.1186/s13059-023-02973-2.

## Background

The relentless sequencing of whole genomes across the tree of life has revealed an enormous diversity in how evolution has shaped them—be it in terms of resulting genome size [1, 2], gene and protein sequence content (e.g., GC content) [3–5], gene length and structure [6, 7], and number of protein-coding genes. Indeed, both the size of genomes and coding genes repertoire vary greatly between species. This is especially true among eukaryotes, which tend to have the highest number of genes and the biggest genomes—differences in genome size can reach up to 60,000-fold [2]. In archaea and bacteria, the genome size and number of genes are generally correlated; however, it is more complicated in eukaryotes, as genome size is mostly impacted by non-coding elements [2]. Other features such as GC content or isoelectric point vary on a gene per gene basis, but studying their distribution at the genome scale has shown that GC content distribution varies considerably across species. This has been associated with adaptation to high temperatures in bacteria and bias in codon usage and is affected by mutation bias toward GC bases in vertebrates [3]. The distribution of isoelectric points of proteins is more uniform among species [4], with the exception of species living in extreme saline environments [8, 9]. While the precise mechanism between the interspecies variation of these variables is not well known, they are probably multifactorial, for example, both GC content [10] of protein-coding genes and isoelectric point of proteins [4] have been shown to be to some extent linked to protein length.

Similarly, the global distribution of protein length can vary between species which evolved under different constraints. A protein’s function is directly dependent on its 3D structure, which ultimately depends on its primary amino acid (aa) sequence and organization into structural domains. A functional protein needs to be long enough to shape itself into structural folds, accommodating one or more functional domains [11] but longer proteins likely have a higher energetic cost (see discussion in [12]). However, the global distribution of protein length within genomes has been scarcely studied, unlike other genome features. The few studies on the subject trace back to early 2000: early studies [13] reported that protein length follows a similarly shaped distribution in the species sequenced at the time — described as either a gamma or log-normal distribution with a long tail more fitting of a power-law distribution [14] — and that proteins were smaller on average in prokaryotes than in eukaryotes. A second study confirmed the divergence between eukaryotic and prokaryotic protein length and noted that protein length distribution was generally uniform within domains of life (bacteria, archaea, and eukaryotes) [15]. Another study reported similar results when comparing eukaryotic and prokaryotic orthologous proteins [16]. A more recent study [17] aimed to revisit these analyses by including more species with a higher taxonomic diversity (1,442 species). The authors confirmed the previous observations but reported that the shape parameter of the distribution was not uniform across species, with up to a two-fold difference. Within eukaryotes, they reported smaller proteins in plants and longer proteins in unicellular eukaryotic species.

All of these previous studies focused on the relative differences of protein length between clades. They relied on summary statistics and did not attempt to explore the causes of underlying differences or similarities in protein length distributions. Thus, several fundamental questions remain unanswered: how different are empirical protein length distributions across the tree of life? Is the difference between Domains due to a complete shift of the distribution, or merely due to an excess of long proteins? How does the variation in protein length distribution compare with the variation in other aspects of genome architecture?

Here, we address these questions by analyzing protein length distributions across 2326 species spanning the three domains of life. We observed a remarkable consistency in the empirical protein length distribution, especially within each domain of life. The near-universality of protein length distribution is particularly striking in comparison with other genomic features, which tend to be much more variable across different species. Additionally, we show that the most divergent exceptions to this observation are likely due to lower genome annotation quality, with annotation errors that escape standard quality assessment methods—which suggests that the true variation in protein length distribution may be even smaller than what we report here.

## Results

### Protein length distribution in the three Domains

We used a dataset of 2326 species, extracted from the Orthologous Matrix (OMA) database [18]. The dataset comprises species from the three domains of life: 485 eukaryotes, 153 archaea, and 1688 bacteria (full list in Additional File 1: Table S1). First, we compared summary statistics of protein length to evaluate how it varies between species and clades (Additional File 2: Table S2). Considering median protein size, proteins are on average smaller in bacteria (270 aa) and archaea (242 aa), compared to eukaryotic proteins (353 aa). Variation in protein length is lower among bacterial and archaeal species (standard deviation 23.3 and 21.3, respectively) than among eukaryotes (standard deviation: 62.5). The higher dispersion in eukaryotes in regard to the median protein length is observed when considering mean and quartiles (Additional File 2: Table S2). However, the variation is smaller for the first quartile protein length than for the median in both eukaryotes (standard deviation: 44.7) and prokaryotes (22.6 in bacteria and 15.0 in archaea) meaning that most of the variance is due to variation in the distributions of larger proteins, which is consistent with previously observed gamma distributions [13, 17].

Based on this observation, we tested whether protein length distribution evolved differently in eukaryotes than in bacteria and archaea. Using a subset of 603 species in our dataset for which a molecular phylogeny was available [19] (listed in Additional File 3: Table S3) and the MvMorph software [20], we tested several models of evolution and different parameters. These included whether the evolution of mean protein length was better described using a model of evolution with global parameters, a model with a different set of parameters in eukaryotes, and a model with three distinct sets of parameters for eukaryotes, archaea, and bacteria. As expected, the models where eukaryotic protein length evolved under a different set of parameters were consistently better supported than the ones where it did not. The best-supported model was a Brownian model of evolution with a distinct set of parameters for the three Domains, suggesting protein length distribution is under different constraints in all three of them (full results in Additional File 4: Table S4). Particularly, it estimated a much higher evolutionary rate in eukaryotes (25,742) than in bacteria (1595.7) and a comparatively lower rate in archaea (525.2). These observations hold true using the median, or first and third quartiles of protein length rather than the mean, indicating it is not meaningfully influenced by the choice of the descriptor.

**Fig. 1:**
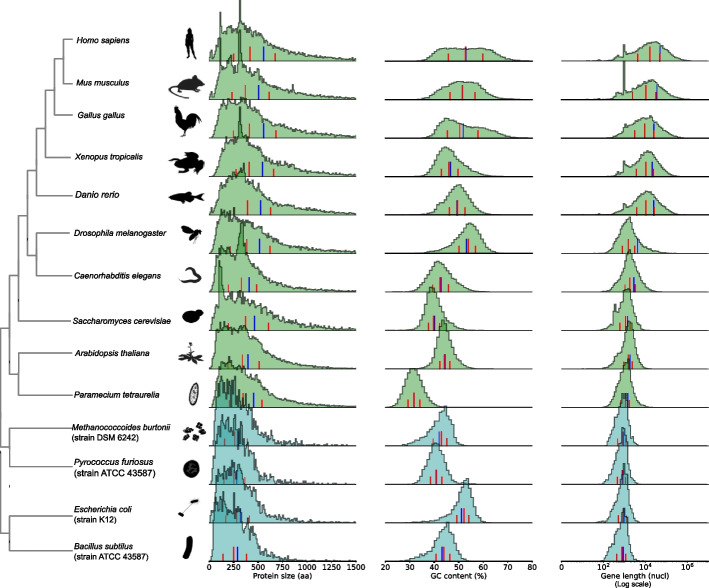
Distributions of protein length, GC content, and gene length (*x*-axis in logarithmic scale), for selected model eukaryotic species (light green), bacterial and archaeal species (blue). Summary statistics are shown as lines at the bottom of the distribution: red lines indicate the first quartile, median, and third quartile, and the blue line indicates the mean. An alternative representation with protein length on a logarithmic scale is available in Additional File 2: Fig. S1

Summary statistics alone are an incomplete reflection of actual protein size distribution. Thus, we plotted empirical protein length distributions for a small subset of diverse, well-annotated model species (Fig. 1).

When considering the length distributions of these representative proteomes, a more homogenous picture emerges. Protein size distributions across species greatly overlap, particularly at the left tail and center of the distribution. The right tail — corresponding to larger proteins — shows more variation. In our representative proteomes, eukaryotes consistently have a higher proportion of proteins longer than 475 aa compared to bacteria and archaea. Individual proteome length distributions display peaks at certain protein lengths that appear to correspond to highly duplicated gene families in their respective lineages. For example, the peak of proteins around 320 aa in humans and mice (*Mus musculus*) corresponds to olfactory receptors, a large protein family expanded by gene duplications in mammals [21].

### Protein length is more uniform across species than other genomic features

**Fig. 2:**
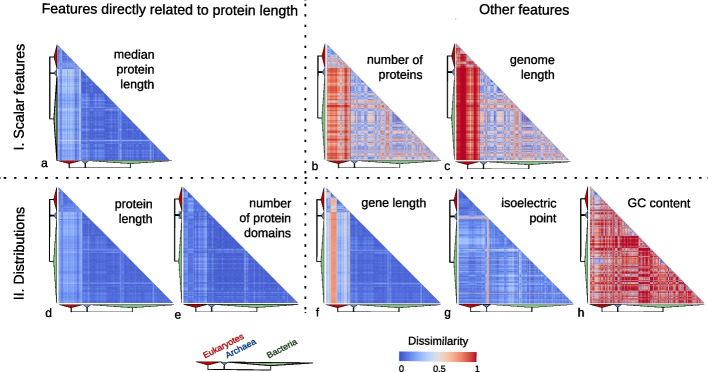
Heatmaps of pairwise species comparison of genomic features. Row and columns are species, ordered by taxonomy. **I** Heatmaps of dissimilarity of three genomic features for every comparison of species. The dissimilarity measure used is an inverted ratio of the pair. An inverted ratio close to 0, in cool colors, means the compared values are identical or very similar. An inverted ratio higher than 0.5, in warm colors, represents a more than 2-fold difference between the highest and lowest values in the pair. Features compared are (**a**) median protein length, (**b**) protein number, and (**c**) genome length. **II** Heatmaps of dissimilarity between distributions of gene-centric features for every comparison of species. The dissimilarity measure used is the Kolmogorov–Smirnov statistics. A statistic of 0 (in blue) means complete overlap between distribution and a statistic to 1 (red) no overlap at all, with intermediate ranges between the two extremes. Compared features are (**d**) protein length distribution, (**e**) protein domain number distribution, (**f**) gene length distribution, (**g**) isoelectric point distribution, and (**h**) GC content distribution. The heatmaps on the left section correspond to variables directly associated with protein length

The similarity of protein length distributions is even more remarkable in comparison with other genomic features, such as the number of protein-coding genes, the number of proteins, the genome length (i.e., the size of the genome in base pairs including coding and noncoding sequence), the GC content distribution, and the gene length (including non-coding elements) distribution (Fig. 2). We quantified this observation by comparing the variability of protein length distribution to the aforementioned genomic features across all species in our dataset. To compare scalar features (i.e., features with one global number per genome, such as total genome length) between two species, we used the “inverted ratio” (IR; see the “Methods” section). An IR close to 0 (in blue) means that the two species have very similar values, while an IR higher than 0.5 (in red) represents a more than 2-fold change between species.

To compare distributions between two species, we used the Kolmogorov–Smirnov statistic (KS). Briefly, KS is the maximum difference between two cumulative density distributions: it ranges from 0, when the distributions are identical, to 1, when the distributions are so different that they do not overlap. KS is a point estimate of the largest divide between two cumulative probability distributions; thus, it can account for variation in both shape and location of the distribution and does not need to estimate continuous distributions from discrete data. However, since it is only a point measurement it may not accurately reflect subtle differences in distribution. Thus, we also performed a comparison using the Jensen-Shannon distance. All values below are reported in KS, but equivalent Jensen-Shannon distances are reported in Additional File 2: Supplementary Results and support similar conclusions.

Results of all pairwise comparisons were used to generate symmetric dissimilarity matrices. On these matrices, we performed Analysis of Similarities (ANOSIM) [22] tests to evaluate whether species within the same Domains tended to have more similar values than species of other domains. Briefly, the ANOSIM R statistics can vary from 1 to − 1, where values close to 1 would mean that species have more similarity within groups (here, domains) than between groups, a value close to 0 indicates that there is no more similarity in species within and between groups, and a value closer to − 1 indicates that there is more similarity between groups than within groups.

These quantitative measures confirmed the small observed difference in protein length across all species pairs, be it in terms of the median (mean IR: 0.15; Fig. 2a, Additional File 2: Fig. S2) or in terms of the entire distributions (mean KS: 0.13; Fig. 2d, Additional File 2: Fig. S3). Protein lengths are particularly similar among archaea and bacteria (mean IR: 0.09; mean KS: 0.08). As noted above, the protein length variation is higher between eukaryotes and the other two domains (mean IR: 0.20; mean KS: 0.13) and this division is strongly and significantly supported by the dissimilarity matrix (IR-ANOSIM R: 0.69, *p*-value: 0.001, KS-ANOSIM R: 0.79, *p*-value: 0.001). Variation within eukaryotes is also higher (mean IR: 0.17; mean KS: 0.13) compared to the very low variation within both archaea and bacteria (mean IR: 0.08 and 0.09, mean KS: 0.07 and 0.07, respectively), although the division of the two taxa is weak but significant (IR-ANOSIM *R*: 0.2, *p*-value: 0.001, KS-ANOSIM *R*: 0.79, p-value: 0.001). Comparing the distributions of the number of structural protein domains per protein-coding gene yielded similar results (mean KS: 0.10, Fig. 2e, Additional File 2: Fig. S4) but with higher similarity between eukaryotes and the other domains (mean KS: 0.14).

In comparison, other features vary considerably more. The number of proteins (Fig. 2b, Additional File 2: Fig. S5) can change by several orders of magnitudes within the same domain, with similar intra-domain variation in eukaryotes and bacteria (mean IR: 0.42, 0.43 respectively), and slightly lower variation in archaea (mean IR: 0.29). As expected, huge inter-domain variations of protein number are observed between eukaryotes and prokaryotes (mean IR of 0.8 and 0.73 between eukaryotes versus archaea and bacteria respectively). We found strong and significant support for the distinction between eukaryotes and bacteria in the dissimilarity matrix (ANOSIM R: 0.88, *p*-value: 0.001), compared to smaller variations and no significant divergences between bacteria and archaea (mean IR of 0.40, ANOSIM R: − 0.02, *p*-value: 0.87).

Genome length (Fig. 2c, Additional File 2: Fig. S6) varies similarly to protein length in archaea and bacteria (mean IR: 0.35, 0.43 respectively). In eukaryotes, the inter- and within-domain magnitude of differences is even higher (mean IR: 0.92 and 0.74, respectively). Thus, like protein number, the distinction in genome length between eukaryotes and other species is strong and significant (ANOSIM *R*: 0.88, *p*-value: 0.001), while there is no strong difference between archaea and bacteria (ANOSIM *R*: 0.04, *p*-value: 0.01).

Gene length includes the untranslated regions (UTR), introns, and exons. As such, it is related to, but not equivalent to, protein length. In archaea and bacteria where UTR are short and there are no introns, the distribution of gene length is as consistent across species as that of protein length (mean KS: 0.07 and 0.08). By contrast, gene length diverges highly between eukaryotes and prokaryotes (Fig. 2f, Additional File 2: Fig. S7; ANOSIM R: 0.92,* p*-value: 0.001). Specifically, gene length distribution varies more (mean KS: 0.35), and with higher intensity (KS from 0 to 1 in extreme cases) within eukaryotes as well as between eukaryotes and the other domains (mean KS: 0.45). Even among eukaryotes, deuterostomes (red line in Fig. 2f) diverge highly from all other species (average KS between deuterostomes and other eukaryotes: 0.48, mean KS between non-deuterostomian eukaryotes: 0.21; ANOSIM *R*: 0.89, *p*-value: 0.001). These divergences can be attributed to the intron–exon structure that leads to longer genes in eukaryotes, and particularly in deuterostomes [23].

Thus, protein number, genome size, and gene length are much more variable than protein length across species. However, regardless of scale, these features appear to follow a similar trend in terms of evolutionary trajectory: faster variation in eukaryotes than in bacteria and archaea. However, these comparisons do not correct for phylogenetic relatedness of species. Thus, as we did in the previous section for protein length, we modeled the evolution of these features across a species tree and tested different evolutionary models. Given the change in magnitude of these features between species, we used logarithmic-transformed data. Similarly as for protein length, the best-supported model of evolution for all three features was a Brownian model of evolution with distinct parameters for the three domains. Again, the rate of evolution predicted by this model was much higher in eukaryotes than in bacteria, and even lower in archaeal species (Additional File 4: Table S4). Finally, for gene length, we tested a model with an additional set of parameters for deuterostomes only, because of the aforementioned higher divergence. This model was better supported than even the ones with distinct parameters for each Domain, confirming a distinct pattern in this group of species.

Likewise GC content distribution is not consistent across domains of life. GC distributions are similar only within some smaller clades. In each domain, the variation (Fig. 2h, Additional File 2: Fig. S8) ranges from a KS of 0 to 1 (i.e., no overlap at all). Barring a few exceptions, GC content distributions are relatively more stable within eukaryotic species (mean KS: 0.55) than within bacteria and archaea (mean KS: 0.76 and 0.71, respectively). In contrast with length-related features, there was no significant support for a divergence between eukaryotes and prokaryotes in the dissimilarity matrix, nor for divergence between archaea and bacteria (ANOSIM *p*-value: 1). We note, however, that the high divergence between GC content distribution is mainly due to difference of location in distribution, leading them in the most extreme case to not overlap. When performing similar analysis over standardized GC distribution, the shape of distribution displays similar uniformity as protein length distribution across life (Additional File 2: Supplementary Results).

Finally, isoelectric point (pH at which a protein is neutrally charged) distributions do not vary as much as GC content across species (Fig. 2g, Additional File 2: Fig. S9). But, as with GC content and in contrast with protein length, isoelectric point is more consistent within eukaryotes (mean KS: 0.10) than bacteria (mean KS: 0.18) and archaea (mean KS: 0.32). The exception was a clade of archaea which deviates particularly from other species in terms of isoelectric point: the Haloarchaea. This is not unexpected as these species are known to reside in extreme pH conditions [8, 24]. Expectedly, and in contrast with protein length distribution, eukaryotes and prokaryotes were not significantly more similar within themselves than between them (ANOSIM p-value: 0.955), although archaea and bacteria had significantly but weakly more similarity within their own domain than between them (ANOSIM R: 0.27, *p*-value: 0.001).

The different trends of evolution that are apparent when comparing distributions pairwise are also apparent when modeling the evolution of GC content and protein isoelectric point across species. When testing different evolutionary models, we found that the best-supported model for isoelectric point evolution is a Brownian Model with one set of parameters for eukaryotes and another for the other species, with a reduced rate in eukaryotes (0.25 vs. 0.49). This is in contrast with all the genomic parameters seen so far that had an elevated rate in eukaryotes. Evolution of GC content was best modeled by an Ornstein–Uhlenbeck process, with a distinct set of parameters in bacteria than for other species. Both of these results confirm that these genomic features follow a distinct trend differing from that of protein length distribution (Additional File 4: Table S4).

### Many protein length distribution outliers are explained by quality issues

**Fig. 3:**
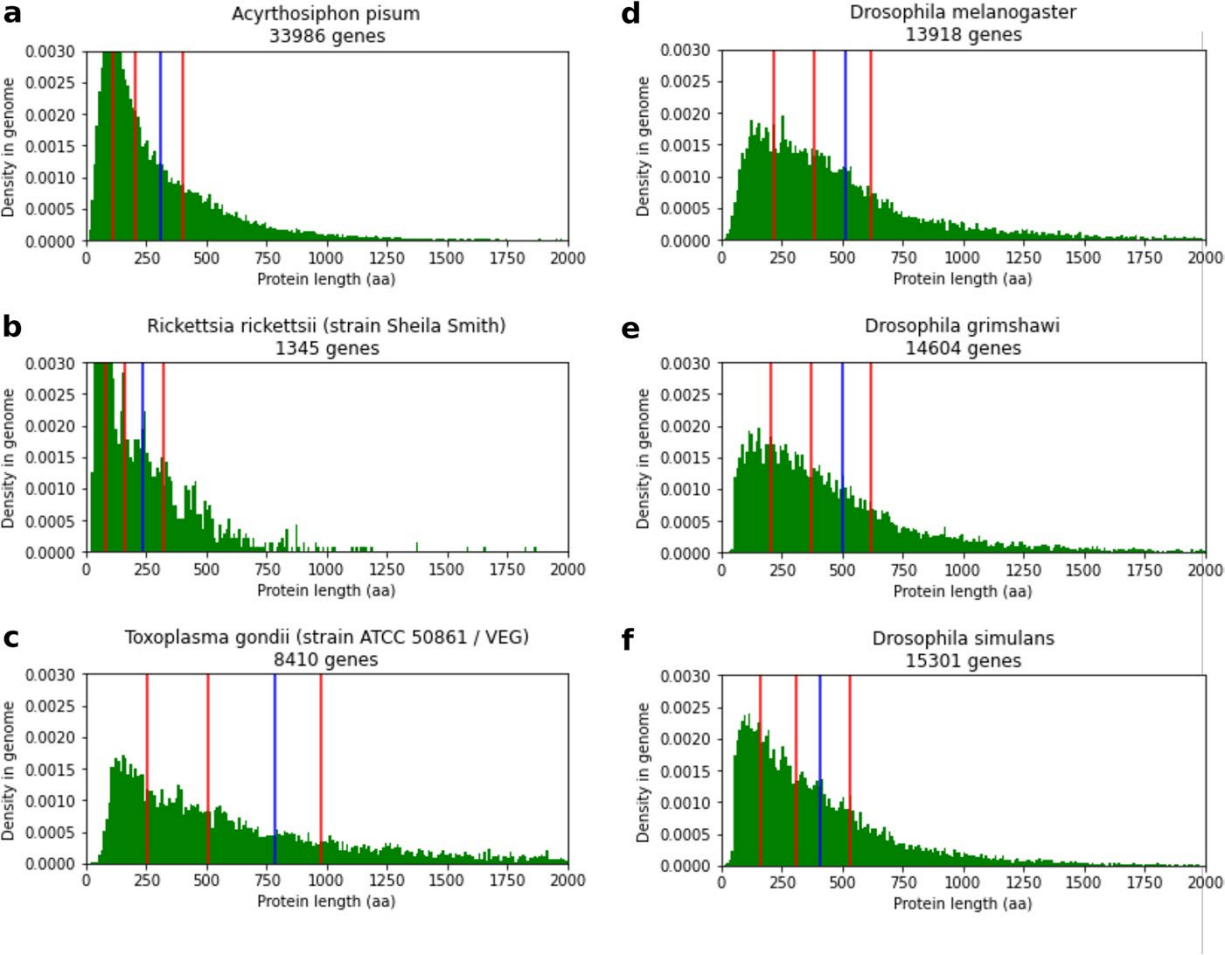
Examples of atypical protein length distributions and distribution heterogeneity between close species. All graphs show the density distribution of protein lengths. The red lines represent the first quartile, median, and third quartile of protein lengths, and the blue lines represent the mean. **a**, **b** Examples of proteomes with an overabundance of small proteins (eukaryote *Acyrthosiphon pisum* (pea aphid) (a), and bacteria *Rickettsia rickettsii* (b)). **c** *Toxoplasma gondii*, an example of a proteome with a high proportion of longer proteins. **d–f** Example of difference in protein length distributions in the *Drosophila* genus. *Drosophila melanogaster* (**d**) has a canonical protein length distribution shape, and similar distributions exist in other *Drosophila* species like *Drosophila grimshawi* (**e**). *Drosophila simulans*, however, shows a relative abundance of small proteins (**f**). An alternative representation with protein length on logarithmic scale is available in Additional File 2: Figure S10

Despite the high overall similarity in protein length distribution, a few species have a protein length distribution that departs from the canonical one, apparent in Fig. 2 as white lines crossing their respective domains. These proteomes have a markedly different shape (examples in Fig. 3a–c) and are often found in species taxonomically related to species with canonical distribution. For example, Fig. 3d–f shows that while *Drosophila melanogaster* (d) and a non-model species of the same genus (e) have a canonical distribution, other close species like *Drosophila simulans* (f) have a comparatively higher abundance of small proteins. Interestingly, a most recent annotation of *Drosophila simulans* has a protein length distribution much closer to *Drosophila melanogaster* and deprecated proteins—which tend to be small proteins—have low transcriptomic support and are predicted as disordered (see Additional File 2: Supplementary Results). Additionally, species with an atypical protein length distribution do not have an obvious biological phenotype in common and given the otherwise consistent protein length distribution among most proteomes, even taxonomically distant ones, we hypothesized that these departures could be artifactual. To test this, we sought to assess the genome and proteome quality of outliers from our pairwise comparison of protein length distribution. For each domain, we identified outlier proteomes in terms of divergence by using Tukey’s fences method. Outliers were defined as proteomes with an average KS dissimilarity with the species of their respective domains of more than 0.2 for eukaryotes, 0.12 for bacteria, and 0.08 for archaea (Additional File 5: Table S5).

Thus, we obtained 37 eukaryotes, 15 archaea, and 122 bacteria as outliers. With the exception of three eukaryotes with a large tail of long proteins, all the divergent proteomes are characterized by a high peak of proteins in a certain length range, most often small proteins (< 100 amino acids). While such distributions were suspected before as being potentially erroneous [17], to our knowledge, this has not yet been demonstrated to be the case. Therefore, we investigated the role of annotation completeness and coding sequence integrity on the occurrence of these outliers.

**Fig. 4:**
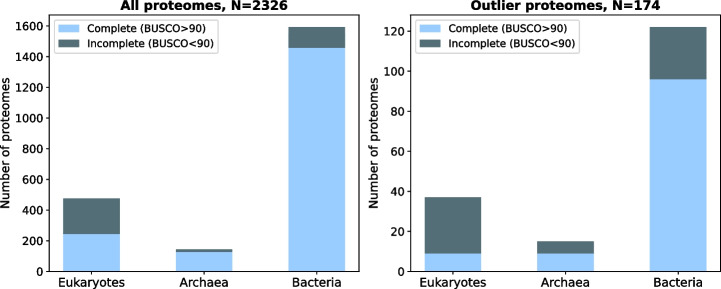
Outlier proteomes in terms of gene length distribution are more likely to be incomplete. Left: Stacked bar of proteomes by domain: mostly complete proteomes in light blue and incomplete proteomes in dark blue. Right: Same representation, with proteomes having the most atypical distribution in regard to their domain (outlier proteomes)

As smaller proteins are commonly observed in the outlier distributions, it could be an indicator of a high proportion of fragmented protein-coding genes, or incomplete representation of the protein-coding gene repertoire. BUSCO [25] is a commonly used method to assess proteome completeness and fragmentation. We ran BUSCO on our dataset of 2326 proteomes and flagged all proteomes for which less than 90% of complete BUSCO genes were found (Fig. 4, Additional File 5: Table S5): 228 of the 485 (47%) eukaryotic proteomes, 17 of the 153 (11%) archaeal proteomes, and 133 of the 1688 (8%) bacterial proteomes. In particular, proteomes with an atypical distribution are enriched in genomes from the incomplete category: 28 of 37 eukaryotic proteomes (75%, 1.6 fold enrichment, Fisher one-sided exact test *p*-value: 3.3e^−4^), 6 of the 15 archaeal proteomes (40%, 3.6-fold enrichment, Fisher one-sided exact test *p*-value: 2.0e^−3^), and 26 of the 122 bacterial proteomes (21%, 2.7-fold enrichment, Fisher one-sided exact test *p*-value:1.1e^−6^), meaning that in these cases, low-quality genomes may be a cause of the atypical distribution.

For the remaining outliers with mostly complete genomes and few fragments according to BUSCO, we investigated whether these atypical distributions could be due to the biology of these species, or merely annotation artifacts not captured by BUSCO. For this experiment, we selected only the most atypical proteomes of each domain (mean KS ≥ 0.2, 24 example species). We checked UniProt [26], RefSeq [27], and the literature for alternative annotation sets (details in Additional File 2: Supplementary Results) and found seven examples where the annotation sets were not consistent and an alternative annotation of the same species had a canonical protein length distribution, suggesting these outliers are mainly due to artifactual annotation.

Three eukaryotic species (the fungal plant pathogen *Ustilago maydis*, and the protozoan obligate parasites *Toxoplasma gondii* (strain VEG) and *Hammondia hammondi*) have diverging distributions characterized by a relatively high amount of proteins longer than 500 amino acids and no overrepresentation of small proteins. In-depth analysis of these proteomes (details in Additional File 2: Supplementary Results) suggested a possible but not conclusive link to their parasitic lifestyles in the case of Apicomplexa.

Combined together, these results suggest that many of the genomes with atypical protein length distribution are characterized by an excess of small proteins, which may be explained in many cases by annotation artifacts — not all of which are captured by conventional quality measures. As for the few which had an excess of larger proteins, we did not find any evidence of artifact, and they all happened to be parasites.

## Discussion

We showed that the distribution of protein size is remarkably consistent within and across the three domains of life, particularly in comparison with other genomic features. Moreover, the exceptions appear to be largely caused by genome annotation artifacts.

While staying within tight boundaries, eukaryotic proteins are noticeably longer than both archaeal and bacterial proteins, which is mostly due to a higher proportion of proteins over 500 amino acids. Other studies have shown that this holds true even when comparing orthologous genes [16, 28]. It is not clear what evolutionary forces drove eukaryotic proteins to be longer, but it may be associated with the higher modularity of eukaryotic proteins [11], their adoption of alternative splicing [29], and an extension of their chaperone (proteins associated with protein folding) repertoire [30].

The few atypical proteomes characterized by an excess of long proteins are found in the *Ustilago* fungal genus and the Apicomplexa phylum, which are both characterized by a partly intracellular parasitic lifestyle. While this excess could be artifactual— and indeed a previous study has suggested that many gene models in Apicomplexa may be erroneously long [31] — it could also be explained by biological particularities. For instance, proteins directed to the apicoplasts, an organelle-specific to the Apicomplexa, typically have signal extensions that make the proteins longer. These proteins are especially long in *T. gondii* [32]—one of the species in our dataset. Second, the process of host-cell invasion in these species rely on proteolytic processing of long protein precursors, located in other organelles specific to the Apicomplexa [33]. This includes proteins involved in host-parasite adhesion [34], which could explain the enrichment of these functions in the longer genes of *Plasmodium falciparum*. While relatively few proteins are well characterized as being part of this process [35], the existence of long protein precursors of smaller functional proteins in the genomes of these species may partly contribute to the observed bias in length distribution.

In contrast, most atypical protein length distributions featured an abundance of small proteins (< 100 aa). Small proteins are known to be involved in important biological processes [36] and are generally under-annotated [37]; however, our analysis suggests that these outliers stem from annotation artifacts [17]. First, none of the well-annotated model species display enrichment in small proteins. Second, proteomes with high numbers of small proteins were more likely to be incomplete or fragmented according to BUSCO. If the atypical protein distribution were representative of the species lifestyle itself, we could expect orthologs of universally conserved genes to be also shorter in the analyzed proteomes and reported as fragmented by BUSCO. However, BUSCO fragments are defined by having a difference of more than 2 standard deviations to the mean length of each gene family [38]; which by definition are highly conserved genes with likely critical biological function. It is unlikely many proteins significantly smaller than their conserved orthologous counterpart exist while retaining their original function; it is more likely they result from a fragmented genomic assembly or annotation artifacts.

Third, for a given species, different annotation sets have different proportions of small proteins. For instance, the recent reannotation of the *Daphnia pulex* genome [39] showed that the high number of small proteins in the previous genome is likely spurious. In this manuscript, we observed a similar trend for the most recent annotation of *Drosophila simulans*, with now deprecated—and likely erroneous—sequences leading to overestimation of small proteins. These errors are possibly due to fragmented assembly leading to genome annotation errors [40, 41] or by the notorious difficulty to discriminate between coding and non-coding ORFs [42, 43] The overabundance of spurious proteins is likely to bias all downstream analyses, leading to an inflated genome size [39, 44], an inflated number of orphan genes [45], and errors in orthology inference (see the ‘Addressing Proteome Quality' section in [46]). Yet, the proportion of small proteins is generally ignored when providing a new annotation set. We propose that the distribution of protein length be used as a new criterion of protein-coding gene quality upon publication, to complement existing quality measures.

The universal character of protein length distribution across life suggests strong, universal selective pressure that would keep a high proportion of the coding sequence between 50 to 500 amino acids. This force does not act uniformly across all proteins, as the length of known active proteins ranges from two amino-acid peptides [47] to more than 30,000 aa [48] our observations support that it does have an effect at the proteome level. The limitation of protein size can be viewed as a simple stochastic process linked to the nature of the genetic code: for any random sequence of codons, the probability of not encountering a stop codon decreases exponentially with length [49]. A similar phenomenon happens due to random mutations—the longer the protein, the higher the chance of accumulating deleterious mutations [50]. One may also consider the length of proteins is subject to a trade-off between keeping a minimum length to perform function and the cost of such proteins for the organism. A longer coding sequence implies increased costs of protein synthesis [12], transcription [51], splicing (in eukaryotes) [52], translation, and chaperone-mediated folding. In this regard, previous studies have shown evidence that the speed of protein folding was subject to evolutionary pressure that favors fast folding proteins [53] and shorter protein lengths [54] via fewer protein domains and shorter inter-domain linker sequences [55]. The authors of these studies have suggested protein length distribution was linked to growth-rate optimization, which applies more strongly to archaea and bacteria, possibly in part due to high differences in population size and to the physical constraints of a non-compartmentalized single cell. Such conclusions are congruent with our observations that protein lengths vary more in eukaryotic species.

While evolution might favor shorter proteins overall, the shape of their distribution across species has been described as a gamma distribution or a log-normal distribution with a long tail fitting of a power-law distribution in the last percentile (rather than a decreasing exponential shape) [13, 14, 17]. This implies the existence of other factors favoring proteins of intermediate length, especially within the 50–500 length range where most of the proteins fall. The modular organization of proteins into structural domains [56] and the stabilization of proteins by folding may explain this [49], as our data suggest that the distribution of the number of protein domains is even more uniform than protein size across species. Thus, accounting for the average length of a protein domain (100 amino acids [57]), it reflects that most functional proteins are composed of one to five protein domains [11]. In this context, the length distribution of proteins could be the result of an optimization process whereby adding new domains may contribute to functional flexibility, albeit at an energy cost for the cell, with diminishing returns. The relationship between protein length and domain organization in the context of resource economy and functional flexibility has been explored before and shown to follow the Menzerath-Altmann law of language [58].

Future investigations of the evolutionary forces acting on protein length would necessitate measuring how the size variation of orthologous sequences varies between species and whether it is an agreement with the overall trend of protein length distribution. Other insights could be gleaned by studying how proteins of different lengths vary in terms of evolutionary rates or differential expression to better characterize how the way they evolve dictates change in overall distributions. For instance, recent studies have shown that housekeeping genes tend to be shorter than other transiently expressed genes [50], making them easier to regulate than longer genes. A full exploration of these hypotheses is beyond the scope of this article.

## Conclusions

Our comprehensive survey of 2326 species has demonstrated that protein length distribution is a remarkably consistent feature across species. This finding stands in stark contrast to other genomic features and suggests that protein length may be subject to unique evolutionary constraints. Our investigation also revealed that unusual length distributions in publicly available proteomes are likely artifacts arising from issues with gene annotation. These findings provide an operational framework for developing a new metric to evaluate gene annotation coherence based on protein length distribution.

Moving forward, our results invoke intriguing questions about the underlying mechanisms shaping gene repertoire evolution, and future studies will be needed to explore the causes of this unexpected consistency.

## Methods

### Dataset acquisition

Data regarding genomic features of all species were extracted from the August 2020 (All.Aug2020) release of the OMA Database [18]. It consists of 2326 species: 485 eukaryotes, 153 archaea, and 1688 bacteria. Genomic and proteomic data available in OMA are from different databases, whose origin can be found on the release page. Genomic features were extracted from OMA as described below:

- *Number of proteins*: We counted the number of protein-coding genes in each species’ proteomes.
- *Genome length*: Genome length data is not available in OMA and not easily obtainable due to the heterogeneity of different data sources. We estimated the genome size by adding for each chromosome or contig, the difference between the 3′-most position (either starting or ending position) of the 3′-most genes and the 5′-most position (either starting or ending position) of the 5′-most gene. This is an estimate that systemically underestimates the real genome length, but is likely to be of a similar order of magnitude.
- *Median protein length*: Median of the protein length of every unique protein in the genome, selecting only one isoform in case of alternative splicing (see below).

#### Isoform and distribution acquisition

All distributions used in this analysis were obtained using one representative protein sequence per protein-coding gene, selecting the main isoform in OMA. These representative isoforms were selected as described in Altenhoff et al. [18], as the isoform with the highest sequence match compared to orthologous sequences across all species. For each gene, the values for the gene-centric metrics were obtained as follows:

- *Protein length*: The length of the string representing the amino-acid sequence of the protein stored in OMA.
- *Gene length*: The difference between the 3′-most position of the gene and the 5′-most position of the gene, as sorted in OMA. These positions account for untranslated regions.
- *Number of protein domains*: The count of the number of domains as stored in OMA, obtained from the Gene3D [59] database (see Altenhoff et al. [60]).
- *GC content*: Proportion of guanine and cytosine in the cDNA sequence, as stored in OMA.
- *Isoelectric point*: The isoelectric point is equal to the pH at which a protein is neutrally charged. It was computed from the protein sequence in OMA, using the ProtParam module within the SeqUtils package from the Biopython package [61].

### Modeling the evolution of genomic features

#### Species tree acquisition

The original species tree used was acquired from [19]. We performed a semi-automatic mapping of the species in the tree to the species in our dataset. Briefly, for each species in our dataset, we selected all species with mentions of its genus in the tree leaves’ label. Then, we performed a manual selection for each species which had one or more matches in the species tree, in order to make sure they corresponded to the correct species. The final retained mapping is available in Additional File 3: Table S3. The tree was rooted so that bacteria and archaea + eukaryotes were monophyletic clades.

We then pruned the tree to retain only leaves shared with our dataset and created a dataframe of average genomic features (average protein length, logarithm of the average gene length, average number of domains, logarithm of the genome size, logarithm of the number of proteins, GC content, isoelectric point of proteins) for this reduced dataset.

#### Phylogenetic independent contrasts

Phylogenetic independent contrasts were computed using the PhylogeneticIndependantConstrast function of the dendropy python library (v4.5.2) [62] on the different genomic parameters. The input tree and data were the ones described above (see the “Species tree acquisition” section).

Spearman correlation between contrasts was computed using the implementation from the SciPy library (v1.9.1).

#### Evolutionary model testing

All maximum likelihood modeling of evolution was done using R (v 4.1.3) and the MvMorph package [20] (v 1.1.6). We used the species tree and data described above (see the “Species tree acquisition” section) and fit multiple models of evolution to these continuous variables. For all variables, we tested:

- A Brownian model of evolution with a single set of parameters.
- A Brownian model of evolution with distinct parameters in bacteria and the clade formed of archaea + eukaryotes.
- A Browmian model of evolution with distinct parameters in eukaryotes.
- A Brownian model of evolution with distinct parameters for eukaryotes, archaea, and bacteria.
- A single parameter Ornstein–Uhlenbeck model of evolution.
- A Ornstein–Uhlenbeck model of evolution with different parameters in archaea.
- A Ornstein–Uhlenbeck model of evolution with distinct parameters in bacteria.
- A Ornstein–Uhlenbeck model of evolution with distinct parameters in eukaryotes.
- A Ornstein–Uhlenbeck model of evolution with distinct parameters in eukaryotes, bacteria, and archaea.

The best fitting model was selected according to the corrected Akaike Information Criterion and the significance of fit for nested models was tested against the models they were nested into with a log-likelihood ratio test.

### Species pairwise comparisons (Heatmaps)

#### Discrete pairwise comparisons (inverted ratio)

The pairwise comparisons (IR) between discrete values (protein number, genome length, median protein length) were computed using the formula:

$${ir}_{x,y}=1-min\left(x,y\right)/max\left(x,y\right)$$

where *x* is the value in species 1 and *y* is the value in species 2.

The score is 0 when the values are equal in both species and goes closer to 1 the more they diverge.

#### Distribution pairwise comparisons

The pairwise comparisons between genewise distributions (protein length, gene length, number of protein domains, GC content, isoelectric comparisons) were done using the two-sample Kolmogorov–Smirnov (KS) statistic. The statistic is computed according to this formula:

$${KSs}_{n,m}=max\left(Fn\left(x\right)-Fm\left(x\right)\right)$$

where *Fn* and *Fm* are the two compared empirical cumulative distributions.

All KS statistics were computed using the SciPy Python package [63].

For the protein length comparison, the mean KS statistic was computed for each species (row), including only comparisons with species of the same Domains (columns).

#### ANOSIM validation

ANOSIM tests [22] were performed on similarity matrices in order to test multiple hypotheses:

- *H0*: There is no more proximity within domains (archaea, bacteria, eukaryotes) than there is between groups.
- *H0*: There is no more proximity within prokaryotes and eukaryotes than between groups.
- *H0*: There is no more proximity within archaea and bacteria than there is between them.

For gene length distribution, we additionally tested:

- *H0*: There is no more proximity within deuterostomes and within other eukaryotes than there is between them.

All ANOSIM computations were done using the anosim function in the stats.distance module of the scikit-bio python library (v0.5.8) with 9999 permutations.

### BUSCO runs

We computed BUSCO [25] statistics on the whole proteome dataset.

First, we generated a FASTA file for each proteome. Then, we ran BUSCO (version 4.1.4) on every individual proteome, using the most specific odb10 reference set for this species. This was determined automatically by mapping datasets to the NCBI taxonomic ID in each species lineage. Indication of the BUSCO set used for each species and all statistics are available in Additional File 1: Table S1.

We divided all proteomes into two sets: complete or incomplete proteomes, based on the number of genes with a complete BUSCO score (not missing nor fragmented). “Complete” proteomes were those with at least 90% of their genes found as “Complete” BUSCO. The complete set was composed of 1942 species, including 253 eukaryotes, 136 archaea, and 1553 bacteria. The incomplete set (< 90% complete BUSCO) was composed of 228 eukaryotic, 17 archaeal, and 133 bacterial proteomes.

### Outlier proteomes definition

We defined proteomes as outliers in regard to their protein length distribution on the basis of the global pairwise comparisons of protein length distribution (see the “Distribution pairwise comparisons” section). We labeled proteomes with an average KS with the species of their respective domain higher than a given threshold as outliers. For each domain, we used Tukey’s fences [64] method to select an appropriate threshold *T*, following this formula:

$$T={Q}_{3}+1.5IQR$$

with ${Q}_{3}$ the third quartile and IQR the interquartile range of the mean KS distribution.

### Third-party proteomes acquisition

For 24 species, with high distribution divergence (Additional File 7: Table S7), we manually queried two sequence databases: Uniprot [26] and RefSeq [27], and downloaded the reference proteome for the same species if available. All proteomes were last downloaded in March 2021.

### Summary statistics and analysis

All summary statistics were computed from the data using the Numpy [65] (v. 1.19.0) Python module. Figures were made using the Seaborn [66] (v. 0.11.0) and Matplotlib [67] (v. 3.3.2) Python module. Fisher’s exact test was run using the implementation in the stats module of the Scipy [63] Python module.

All code was run with Python v. 3.7.7.

### Gene Ontology enrichment analysis of long genes

We investigated the functional representation of long genes in proteomes with outlier distribution characterized by an abundance of long proteins. The analysis was carried out for *Ustilago maydis* and all representatives of the Apicomplexa clade in the dataset. Sequence data and GO annotations were extracted from OMA. One species (*Hammondia hammondi*) had no existing GO annotation; thus, proteins were automatically annotated using the “Gene Ontology Functional Prediction” of the OMA browser. Briefly, genes from *H. hammondia* were mapped to their closest sequence in the OMA database, and then GO terms were propagated to them from genes in the same Orthologous Group [60, 68].

All enrichment analyses were run in Python using the goatools library [69]. For each species, the enrichment procedure was performed using all genes from that species with a protein size greater than different length thresholds (1000, 2000, 3000, 4000, 5000 aa) as study sets. Two background populations were used: either all genes from that species or all genes from the 25 Apicomplexans in the OMA database of the same length requirement as the foreground population. Only GO terms enriched with a Bonferroni-corrected *p*-value <  = 0.05 were considered significant. Results were plotted and visualized using the Go-Figure software [70]. The analysis described here was run with Python v. 3.7.7.

## Supplementary Information

**Additional file 1:** **Table S1.** Per species protein length data summary and BUSCO results.

**Additional file 2.** Supplementary Materials and Results. This file include all Supplementary Results: alternative distribution comparisons using Jensen-Shannon distance and standardized distribution; analysis of correlations between genomic features; analysis of “dubious” proteins contributing to atypical distribution and its support; comparisons of outliers proteomes with other annotation sets and functional analysis of proteomes with abundance of long proteins. It also includes all Supplementary Figures and Supplementary Table S2 [72–82].

**Additional file 3:** **Table S3.** Mapping of species in our original dataset to the species in Hug et al., 2016 [19], and associated data used in MvMorph.

**Additional file 4:** **Table S4.** Aggregated results of tests for different models of evolution for each evaluated genomic feature. Parameters of the models, maximum likelihood estimation and model ranking according to AICw are reported

**Additional file 5:** **Table S5.** BUSCO quality score for every species dataset and Mean KS of protein length distribution according to their taxonomic Domain. Proteomes labeled as outlier in this current species are also reported.

**Additional file 6:** **Table S6.** Report of assessed features for proteins in the Drosophila genome, whether they are deprecated in the most recent annotationor not.

**Additional file 7:** **Table S7.** List of complete proteomes with atypical distribution and comparisons with other annotation sets.

**Additional file 8.** Review history.

## Data Availability

Analyses described in this paper were done using Jupyter Notebook. The notebook and the data generated during the analyses are available on Zenodo [71] at 10.5281/zenodo.7712057 under the Creative Commons Attribution 4.0 International license.

## References

[1] Wright SI. Evolution of Genome Size [Internet]. eLS. Chichester, UK: John Wiley & Sons, Ltd; 2017. p. 1–6. Available from: 10.1002/9780470015902.a0023983

[2] Elliott TA, Gregory TR (2015). What’s in a genome? The C-value enigma and the evolution of eukaryotic genome content. Philos Trans R Soc Lond B Biol Sci.

[3] Li X-Q, Du D (2014). Variation, evolution, and correlation analysis of C+G content and genome or chromosome size in different kingdoms and phyla. PLoS ONE.

[4] Kiraga J, Mackiewicz P, Mackiewicz D, Kowalczuk M, Biecek P, Polak N (2007). The relationships between the isoelectric point and: length of proteins, taxonomy and ecology of organisms. BMC Genomics.

[5] Kozlowski LP (2017). Proteome-pI: proteome isoelectric point database. Nucleic Acids Res.

[6] Yandell M, Mungall CJ, Smith C, Prochnik S, Kaminker J, Hartzell G (2006). Large-scale trends in the evolution of gene structures within 11 animal genomes. PLoS Comput Biol.

[7] Rogozin IB, Carmel L, Csuros M, Koonin EV (2012). Origin and evolution of spliceosomal introns. Biol Direct.

[8] Falb M, Pfeiffer F, Palm P, Rodewald K, Hickmann V, Tittor J (2005). Living with two extremes: conclusions from the genome sequence of Natronomonas pharaonis. Genome Res.

[9] Kennedy SP, Ng WV, Salzberg SL, Hood L, DasSarma S (2001). Understanding the adaptation of Halobacterium species NRC-1 to its extreme environment through computational analysis of its genome sequence. Genome Res.

[10] Oliver JL, Marín A (1996). A relationship between GC content and coding-sequence length. J Mol Evol.

[11] Middleton S, Song T, Nayak S (2010). Length constraints of multi-domain proteins in metazoans. Bioinformation.

[12] Lipman DJ, Souvorov A, Koonin EV, Panchenko AR, Tatusova TA (2002). The relationship of protein conservation and sequence length. BMC Evol Biol.

[13] Zhang J (2000). Protein-length distributions for the three domains of life. Trends Genet.

[14] Jain R, Ramakumar S (1999). Stochastic dynamics modeling of the protein sequence length distribution in genomes: implications for microbial evolution. Physica A.

[15] Xu L, Chen H, Hu X, Zhang R, Zhang Z, Luo ZW (2006). Average gene length is highly conserved in prokaryotes and eukaryotes and diverges only between the two kingdoms. Mol Biol Evol.

[16] Brocchieri L, Karlin S (2005). Protein length in eukaryotic and prokaryotic proteomes. Nucleic Acids Res.

[17] Tiessen A, Pérez-Rodríguez P, Delaye-Arredondo LJ (2012). Mathematical modeling and comparison of protein size distribution in different plant, animal, fungal and microbial species reveals a negative correlation between protein size and protein number, thus providing insight into the evolution of proteomes. BMC Res Notes.

[18] Altenhoff AM, Train C-M, Gilbert KJ, Mediratta I, Mendes de Farias T, Moi D, OMA orthology in,  (2021). website overhaul, conserved isoforms, ancestral gene order and more. Nucleic Acids Res.

[19] Hug LA, Baker BJ, Anantharaman K, Brown CT, Probst AJ, Castelle CJ (2016). A new view of the tree of life. Nat Microbiol.

[20] Clavel J, Escarguel G, Merceron G (2015). Mv morph : An r package for fitting multivariate evolutionary models to morphometric data. Methods Ecol Evol Wiley.

[21] Niimura Y, Nei M (2007). Extensive gains and losses of olfactory receptor genes in mammalian evolution. PLoS ONE.

[22] Clarke KR. Non-parametric multivariate analyses of changes in community structure. Aust J Ecol. John Wiley & Sons, Ltd; 1993;18:117–43.

[23] McCoy MJ, Fire AZ (2020). Intron and gene size expansion during nervous system evolution. BMC Genomics.

[24] Moran-Reyna A, Coker JA (2014). The effects of extremes of pH on the growth and transcriptomic profiles of three haloarchaea. F1000Res.

[25] Seppey M, Manni M, Zdobnov EM (2019). BUSCO: assessing genome assembly and annotation completeness. Methods Mol Biol.

[26] UniProt Consortium (2021). UniProt: the universal protein knowledgebase in 2021. Nucleic Acids Res.

[27] O’Leary NA, Wright MW, Brister JR, Ciufo S, Haddad D, McVeigh R (2016). Reference sequence (RefSeq) database at NCBI: current status, taxonomic expansion, and functional annotation. Nucleic Acids Res.

[28] Wang D, Hsieh M, Li W-H (2005). A general tendency for conservation of protein length across eukaryotic kingdoms. Mol Biol Evol.

[29] Zhuang Y, Ma F, Li-Ling J, Xu X, Li Y (2003). Comparative analysis of amino acid usage and protein length distribution between alternatively and non-alternatively spliced genes across six eukaryotic genomes. Mol Biol Evol.

[30] Rebeaud ME, Mallik S, Goloubinoff P, Tawfik DS. On the evolution of chaperones and co-chaperones and the exponential expansion of proteome complexity [Internet]. bioRxiv. 2020:2020.06.08.140319. Available from: https://www.biorxiv.org/content/10.1101/2020.06.08.140319v1.full. Cited 14 Apr 2021.

[31] Wakaguri H, Suzuki Y, Sasaki M, Sugano S, Watanabe J (2009). Inconsistencies of genome annotations in apicomplexan parasites revealed by 5’-end-one-pass and full-length sequences of oligo-capped cDNAs. BMC Genomics.

[32] Seliverstov AV, Zverkov OA, Istomina SN, Pirogov SA, Kitsis PS (2015). Comparative analysis of apicoplast-targeted protein extension lengths in apicomplexan parasites. Biomed Res Int.

[33] Blackman MJ, Bannister LH (2001). Apical organelles of Apicomplexa: biology and isolation by subcellular fractionation. Mol Biochem Parasitol.

[34] Li H, Child MA, Bogyo M (2012). Proteases as regulators of pathogenesis: examples from the Apicomplexa. Biochim Biophys Acta.

[35] Silmon de Monerri NC, Flynn HR, Campos MG, Hackett F, Koussis K, Withers-Martinez C (2011). Global identification of multiple substrates for Plasmodium falciparum SUB1, an essential malarial processing protease. Infect Immun.

[36] Su M, Ling Y, Yu J, Wu J, Xiao J (2013). Small proteins: untapped area of potential biological importance. Front Genet.

[37] Frith MC, Forrest AR, Nourbakhsh E, Pang KC, Kai C, Kawai J (2006). The abundance of short proteins in the mammalian proteome. PLoS Genet.

[38] Simão FA, Waterhouse RM, Ioannidis P, Kriventseva EV, Zdobnov EM (2015). BUSCO: assessing genome assembly and annotation completeness with single-copy orthologs. Bioinformatics.

[39] Ye Z, Xu S, Spitze K, Asselman J, Jiang X, Ackerman MS (2017). A New Reference Genome Assembly for the Microcrustacean Daphnia pulex. G3.

[40] Florea L, Souvorov A, Kalbfleisch TS, Salzberg SL (2011). Genome assembly has a major impact on gene content: a comparison of annotation in two Bos taurus assemblies. PLoS ONE.

[41] Nehrt NL, Clark WT, Radivojac P, Hahn MW (2011). Testing the ortholog conjecture with comparative functional genomic data from mammals. PLoS Comput Biol.

[42] Das S, Yu L, Gaitatzes C, Rogers R, Freeman J, Bienkowska J (1997). Biology’s new Rosetta stone. Nature.

[43] Fickett JW (1995). ORFs and genes: how strong a connection?. J Comput Biol.

[44] Denton JF, Lugo-Martinez J, Tucker AE, Schrider DR, Warren WC, Hahn MW (2014). Extensive error in the number of genes inferred from draft genome assemblies. PLoS Comput Biol.

[45] Prabh N, Rödelsperger C (2016). Are orphan genes protein-coding, prediction artifacts, or non-coding RNAs?. BMC Bioinformatics.

[46] Nevers Y, Defosset A, Lecompte O, Pontarotti P (2020). Orthology: promises and challenges. Evolutionary Biology—A Transdisciplinary Approach.

[47] Henry J, Favrel P, Boucaud-Camou E (1997). Isolation and identification of a novel Ala-Pro-Gly-Trp-amide-related peptide inhibiting the motility of the mature oviduct in the cuttlefish. Sepia Officinalis Peptides.

[48] Labeit S, Kolmerer B (1995). Titins: giant proteins in charge of muscle ultrastructure and elasticity. Science.

[49] White SH (1994). The evolution of proteins from random amino acid sequences: II. Evidence from the statistical distributions of the lengths of modern protein sequences. J Mol Evol.

[50] Lopes I, Altab G, Raina P, de Magalhães JP (2021). Gene Size Matters: An Analysis of Gene Length in the Human Genome. Front Genet.

[51] Urrutia AO, Hurst LD (2003). The signature of selection mediated by expression on human genes. Genome Res.

[52] Castillo-Davis CI, Mekhedov SL, Hartl DL, Koonin EV, Kondrashov FA (2002). Selection for short introns in highly expressed genes. Nat Genet.

[53] Debès C, Wang M, Caetano-Anollés G, Gräter F (2013). Evolutionary optimization of protein folding. PLoS Comput Biol.

[54] Wang M, Caetano-Anollés G (2009). The evolutionary mechanics of domain organization in proteomes and the rise of modularity in the protein world. Structure.

[55] Wang M, Kurland CG, Caetano-Anollés G (2011). Reductive evolution of proteomes and protein structures. Proc Natl Acad Sci U S A.

[56] Doolittle RF. The multiplicity of domains in proteins. Annual Reviews 4139 El Camino Way, P.O. Box 10139, Palo Alto, CA 94303–0139, USA; 2003. Available from: https://www.annualreviews.org/doi/abs/10.1146/annurev.bi.64.070195.001443. Cited 6 Aug 2021.

[57] Wheelan SJ, Marchler-Bauer A, Bryant SH (2000). Domain size distributions can predict domain boundaries. Bioinformatics.

[58] Shahzad K, Mittenthal JE, Caetano-Anollés G (2015). The organization of domains in proteins obeys Menzerath-Altmann’s law of language. BMC Syst Biol.

[59] Lam SD, Dawson NL, Das S, Sillitoe I, Ashford P, Lee D (2016). Gene3D: expanding the utility of domain assignments. Nucleic Acids Res.

[60] Altenhoff AM, Glover NM, Train C-M, Kaleb K, Warwick Vesztrocy A, Dylus D (2018). The OMA orthology database in 2018: retrieving evolutionary relationships among all domains of life through richer web and programmatic interfaces. Nucleic Acids Res.

[61] Cock PJA, Antao T, Chang JT, Chapman BA, Cox CJ, Dalke A (2009). Biopython: freely available Python tools for computational molecular biology and bioinformatics. Bioinformatics Oxford Academic.

[62] Sukumaran J, Holder MT (2010). DendroPy: a Python library for phylogenetic computing. Bioinformatics.

[63] Virtanen P, Gommers R, Oliphant TE, Haberland M, Reddy T, Cournapeau D (2020). SciPy 1.0: fundamental algorithms for scientific computing in Python. Nat Methods.

[64] Tukey JW (1977). Exploratory data analysis.

[65] Harris CR, Millman KJ, van der Walt SJ, Gommers R, Virtanen P, Cournapeau D (2020). Array programming with NumPy. Nature.

[66] Waskom M (2021). seaborn: statistical data visualization. J Open Source Softw.

[67] Hunter JD (2007). Matplotlib: A 2D Graphics Environment. Comput Sci Eng.

[68] Altenhoff AM, Škunca N, Glover N, Train C-M, Sueki A, Piližota I (2015). The OMA orthology database in 2015: function predictions, better plant support, synteny view and other improvements. Nucleic Acids Res.

[69] Klopfenstein DV, Zhang L, Pedersen BS, Ramírez F, Warwick Vesztrocy A, Naldi A (2018). GOATOOLS: A Python library for Gene Ontology analyses. Sci Rep.

[70] Reijnders MJMF, Waterhouse RM (2021). Summary Visualizations of Gene Ontology Terms With GO-Figure!. Front Bioinform.

[71] Nevers Y, Glover N, Dessimoz C, Lecompte O. Protein length distribution is remarkably consistent across Life. Zenodo. 2023. 10.5281/zenodo.7712057

[72] Jumper J, Evans R, Pritzel A, Green T, Figurnov M, Ronneberger O (2021). Highly accurate protein structure prediction with AlphaFold. Nature.

[73] Gene Ontology Consortium (2021). The Gene Ontology resource: enriching a GOld mine. Nucleic Acids Res.

[74] Ashburner M, Ball CA, Blake JA, Botstein D, Butler H, Cherry JM (2000). Gene ontology: tool for the unification of biology The Gene Ontology Consortium. Nat Genet.

[75] Brumlik MJ, Wei S, Finstad K, Nesbit J, Hyman LE, Lacey M (2004). Identification of a novel mitogen-activated protein kinase in Toxoplasma gondii. Int J Parasitol.

[76] Wei S, Marches F, Daniel B, Sonda S, Heidenreich K, Curiel T (2002). Pyridinylimidazole p38 mitogen-activated protein kinase inhibitors block intracellular Toxoplasma gondii replication. Int J Parasitol.

[77] Wei F, Wang W, Liu Q (2013). Protein kinases of Toxoplasma gondii: functions and drug targets. Parasitol Res.

[78] Sibley LD (2004). Intracellular parasite invasion strategies. Science.

[79] Suarez C, Lentini G, Ramaswamy R, Maynadier M, Aquilini E, Berry-Sterkers L (2019). A lipid-binding protein mediates rhoptry discharge and invasion in Plasmodium falciparum and Toxoplasma gondii parasites. Nat Commun.

[80] Pedregosa F, Varoquaux G, Gramfort A, Michel V, Thirion B, Grisel O (2011). Scikit-learn: Machine Learning in Python. J Mach Learn Res.

[81] Altschul SF, Gish W, Miller W, Myers EW, Lipman DJ (1990). Basic local alignment search tool. J Mol Biol.

[82] Camacho C, Coulouris G, Avagyan V, Ma N, Papadopoulos J, Bealer K (2009). BLAST+: architecture and applications. BMC Bioinformatics.

