# Supplementary material for: Protein length distribution is remarkably uniform across the tree of life
Source: Genome Biol. 2023 Jun 8;24:135. doi: 10.1186/s13059-023-02973-2 (PMC10251718; doi:10.1186/s13059-023-02973-2)

Supplementary materials

Supplementary Data 1: Archive with image files of protein length distribution for each proteome in the dataset. Available on Zenodo at https://doi.org/10.5281/zenodo.7712057 .

Supplementary Data 2: Dataset files and associated Jupyter Notebook for the main analyses of the paper (species comparison of genomic features, outlier protein length investigation, etc). Available on Zenodo at https://doi.org/10.5281/zenodo.7712057 .

Supplementary Data 3: Jupyter Notebook for the gene ontology enrichment analysis in species with an outlier distribution characterized by long proteins, and resulting files of the enrichment. Available on Zenodo at https://doi.org/10.5281/zenodo.7712057 .

Table S1: Data summary and BUSCO sets.

Table S2: Summary statistics of protein length for genomes in the dataset, by domains.

	**eukaryotes**		**bacteria**		**archaea**	
	Average	Std dev	Average	Std dev	Average	Std dev
First Quartile	216.8	41.9	161.5	22.1	145.3	12.2
Median	372.3	55.8	270.8	22.5	245.5	14.5
Mean	486.3	83.2	317.3	24.9	286.3	15.6
Third Quartile	604.1	98.1	407.4	25.7	373.0	16.0

Table S3: Mapping of species in our original dataset to the species in Hug et al, 2016 [19], and associated data used in MvMorph.

Table S4: Aggregated results of tests for different models of evolution for each evaluated genomic feature. Parameters of the models, maximum likelihood estimation and model ranking according to AICw are reported.

Table S5: BUSCO quality score for every species dataset and Mean KS of protein length distribution according to their taxonomic Domain. Proteomes labeled as outlier in this current species are also reported.

Table S6: Report of assessed features for proteins in the Drosophila genome, whether they are deprecated in the most recent annotation (Dubious set) or not.

Table S7: Complete proteomes with atypical distribution and comparisons with other annotation sets.

Supplementary results

- Distribution comparison using Jensen-Shannon distance
    - Tables S8-S17
- Standardized distribution comparisons
    - Tables S18-S25
- Correlations between genomic features
- Atypical protein length is attributable to weakly supported protein sequence
- Comparison of outliers proteomes with other annotation sets
- Functional analysis of proteomes with abundance of long proteins

Supplementary results

Distribution comparison using Jensen-Shannon distance

Kolmogorov-Smirnov divergence is a point estimate of the difference between distributions based on maximal difference between their cumulative distribution function. It provides an easy to interpret measurement of the difference between distributions of samples with minimal transformation of the data but does not provide a global measure of their divergence over their interval. In order to make sure this choice did not impact our results, we reran our pairwise comparisons between distributions using the Jensen-Shannon distance measure. This measurement evaluates the similarity between pairs of probability distributions, and ranges from 0 to 1, where 0 is least diverged and 1 is most diverged. In the table below, we summarize all results obtained using this measure on our dataset.
While Jensen-Shannon (JS) distance measurements were often higher than the Kolmogorov-statistics, this was the case over all of the feature comparisons, and thus yielded similar results in terms of relative comparisons. We recapitulate these results below, and report all pairwise comparisons in Tables S8-S25.

Protein length distribution

As with the KS statistics, protein length distributions are more similar within the bacterial and archaeal Domains than between eukaryotes. Similarly, the average distance is especially high between eukaryotes and prokaryotes. Similar results are observed when comparing the distribution of log-protein length rather than protein length directly. In every case, on the basis of protein length, higher similarity within Domains than between Domains is supported by the similarity matrix, though the distinction between archaea and bacteria is weaker.

Average distance

Distance metric / Comparison	Kolmogorov-Smirnov	Jensen-Shannon	Jensen-Shannon (Log Length)

Within eukaryota	0.13	0.16	0.17
Within bacteria	0.08	0.12	0.14
Within archaea	0.07	0.10	0.11
Between eukaryota-Other	0.20	0.25	0.26

Table S8: Average divergence of protein length distributions. Average divergence within and between domains for protein length distributions using either the Kolmogorov-Smirnov statistics or the Jensen-Shannon distance as a metric, on non-transformed and logarithmic data.

ANOSIM results

Distance metric / H1	Kolmogorov-Smirnov	Jensen-Shannon	Jensen-Shannon (Log Length)
One group per domain	0.66 (p:0.001)	0.68 (p:0.001)	0.66 (p:0.001)
Two groups: eukaryota - Other	0.80 (p:0.001	0.84 (p:0.001)	0.81 (p:0.001)
Two groups: archaea - bacteria	0.21 (p:0.001)	0.14 (p:0.001)	0.13 (p:0.001)

Table S9: ANOSIM results for tests of different hypotheses on dissimilarity matrices of protein length distributions. ANOSIM R-statistic and associated p-value of hypotheses tests that two groups of species are not closer to each other in the similarity matrix of protein length distributions; using either the Kolmogrov-Smirnov statistic and Jensen-Shanon distance as a metric, on non-transformed or log-transformed data. The associated H1 hypothesis is written in the first column.

Number of domains

As with the KS statistics, the distribution of the number of domains is on average more similar than protein length distribution, within and between clades. Again, the highest dissimilarity is noted within eukaryotes and between eukaryotes and other domains. The weak distinction between archaea and bacteria supported by the KS statistics matrix is not supported when using JS.

Average values

Distance metric / Comparison	Kolmogorov-Smirnov	Jensen-Shannon
Within eukaryota	0.13	0.14
Within bacteria	0.07	0.08

Within archaea	0.07	0.08
Between eukaryota-Other	0.14	0.15

Table S10: Average divergence of the distribution of the number of domains. Average divergence within and between domains for distributions of numbers of protein domains; using either the Kolmogorov-Smirnov statistics or the Jensen-Shannon distance as a metric.

ANOSIM results

H1 \ Distance metric	Kolmogorov-Smirnov	Jensen-Shannon
One group per domain	0.46 (p:0.001)	0.55 (p:0.001)
Two groups: eukaryota - Other	0.50 (p:0.001	0.60 (p:0.001)
Two groups: archaea - bacteria	0.28 (p:0.001)	0.02 (p:0.1916)

Table S11: ANOSIM results for tests of different hypotheses on dissimilarity matrices of distributions of number of domains. ANOSIM R-statistic and associated p-value for hypotheses tests that two groups of species are not closer to each other in the similarity matrix of the distributions of number of domains; using either the Kolmogrov-Smirnov statistic and Jensen-Shanon distance as a metric. The associated H1 hypothesis is written in the first column.

Gene length

As with the KS statistics, differences in gene length distribution have a similar pattern than protein length distribution in archaea and bacteria but much higher distance within eukaryotes, and between eukaryotes and other Domains. Again, within eukaryota, the gene length distribution of deuterostomia varies strongly and significantly from the rest.

As with protein length, the distribution of log-gene length rather than gene length yields similar distance values, showing the results are robust to data transformation.

Average values

Comparison \ Distance metric	Kolmogorov-Smirnov	Jensen-Shannon	Jensen-Shannon (Log Gene Length)
Within eukaryota	0.35	0.39	0.40
Within bacteria	0.08	0.12	0.14

Within archaea	0.07	0.10	0.12
Between eukaryota-Other	0.45	0.48	0.48
Between Deuterostomia - other eukaryota	0.48	0.60	0.60
Between non-deuterostomia eukaryotes	0.21	0.27	0.27

Table S12: Average divergence of gene length distributions. Average divergence within and between groups of species for gene length distributions; using either the Kolmogorov-Smirnov statistics or the Jensen-Shannon distance as a metric, on non-transformed and logarithmic data.

ANOSIM results

Distance metric / H1	Kolmogorov-Smirnov	Jensen-Shannon	Jensen-Shannon (Log Gene Length)
One group per domain	0.74 (p:0.001)	0.73 (p:0.001)	0.73 (p:0.001)
Two groups: eukaryota - Other	0.92 (p:0.001)	0.93 (p:0.001)	0.93 (p:0.001)
Two groups: archaea - bacteria	0.21 (p:0.001)	0.14 (p:0.001)	0.13 (p:0.001)
Two groups: Deuterostomia - Other	0.89 (p:0.001)	0.89 (p:0.001)	0.89 (p:0.001)

Table S13: ANOSIM results for tests of different hypotheses on dissimilarity matrices of gene length distributions. ANOSIM R-statistic and associated p-value for test of hypotheses that two groups of species are not closer to each other in the similarity matrix of gene length distributions; using either the Kolmogrov-Smirnov statistic and Jensen-Shanon distance as a metric on non-transformed or log-transformed data. The associated H1 hypothesis is written in the first column.

GC content

Using JS distance for comparing distribution, we observe similar patterns as that with the KS statistics. The average distance between species is high, and is especially strong within archaea and bacteria, much higher than the difference in terms of protein length distribution. On the basis of the similarity matrix, a higher proximity of species within one domain than between domains is not supported.

Average values

Comparison \ Distance metric	Kolmogorov-Smirnov	Jensen-Shannon
Within eukaryota	0.55	0.59
Within bacteria	0.76	0.76
Within archaea	0.71	0.72

Table S14: Average divergence of GC content distributions. Average divergence within domains of GC content distributions; using either the Kolmogorov-Smirnov statistics or the Jensen-Shannon distance as a metric.

ANOSIM results

H1 \ Distance metric	Kolmogorov-Smirnov	Jensen-Shannon
One group per domain	-0.09 (p:1)	-0.08 (p:1)
Two groups: eukaryota - Other	-0.11 (p:1	-0.10 (p:1)
Two groups: archaea - bacteria	0.06 (p:1)	0.06 (p:1)

Table S15. ANOSIM results for tests of different hypotheses on dissimilarity matrices of GC content distributions. ANOSIM R-statistic and associated p-value for test of hypotheses that two groups of species are not closer to each other in the similarity matrix of GC content distributions; using either the Kolmogrov-Smirnov statistic and Jensen-Shanon distance as a metric. The associated H1 hypothesis is written in the first column.

Isoelectric point

JS distance between isoelectric points displays a similar pattern as that of the KS statistics. Relatively high average similarity between eukaryotes, comparable to protein length distribution, but a much higher distance within bacteria and especially archaea. The similarity matrix significantly supports a higher similarity within archaea and bacteria than between them, though weakly (ANOSIM R: 0.25).

Average values

Comparison \ Distance metric	Kolmogorov-Smirnov	Jensen-Shannon
Within eukaryota	0.10	0.12
Within bacteria	0.18	0.21

Within archaea	0.32	0.32

Table S16: Average divergence of isoelectric point distributions. Average divergence within domains of isoelectric point distributions; using either the Kolmogorov-Smirnov statistics or the Jensen-Shannon distance as a metric.

ANOSIM results

Distance metric / H1	Kolmogorov-Smirnov	Jensen-Shannon
One group per domain	-0.08 (p:0.0001)	0.09 (p:0.0001)
Two groups: eukaryota - Other	-0.02 (p:0.95)	-0.01 (p:0.84)
Two groups: archaea - bacteria	0.27 (p:0.0001)	0.25 (p:0.0001)

Table S17: ANOSIM results for tests of different hypotheses on dissimilarity matrices of isoelectric point distributions. ANOSIM R-statistic and associated p-value for test of hypotheses that two groups of species are not closer to each other in the similarity matrix of isoelectric point distributions; using either the Kolmogrov-Smirnov statistic and Jensen-Shanon distance as a metric. The associated H1 hypothesis is written in the first column.

Standardized distribution comparisons

Our main analyses of genomic feature distribution were based on pairwise comparisons of the distributions, accounting for shape and location of the values. We reiterated the analysis over standardized distribution in order to assess whether the observed higher similarity in protein length distribution differed when comparing only the shape of the distribution. To do this, for any given genomic feature from a given species, we subtracted each observation by the mean of their distribution and divided the result by the standard deviation of the distribution, resulting in – for each feature -- distributions with a mean of 0 and a standard deviation of 1.

When comparing standardized distribution, the Jensen-Shannon distance is, over all comparisons, much greater than the Kolmogorov-Smirnov statistic, but the relative comparison between clades keeps a similar trend regardless of the dissimilarity measure used.

When comparing standardized distribution, results are overall comparable to the comparison of distribution only with one notable difference. Standardized GC content distribution keeps overall low dissimilarity within and across Domains, and is generally more similar within Domains than across, showing a similar trend than protein length distribution. This is in stark contrast to what we observe when comparing the original distributions; and show that the previously noted higher distance is mainly due to change in the location of distribution rather than its shape.

Protein length distribution

When comparing the shape of distributions only, we observe the same overall patterns as when comparing the distributions themselves, with eukaryotes being slightly more dissimilar between themselves than bacteria and archaea, and a higher distance between eukaryotes and other species. Accordingly, the similar matrix over shape still supports a significant distinction between eukaryotes and prokaryotes. However, the weak support for a distinction between an archaeal and bacterial group is not supported when comparing shape only.

Average distance

Distance metric / Comparison	Kolmogorov-Smirnov	Jensen-Shannon	Kolmogorov -Smirnov (Log Length)	Jensen-Shannon (Log Length)
Overall	0.09	0.19	0.04	0.11
Within eukaryota	0.09	0.18	0.03	0.10
Within bacteria	0.07	0.17	0.03	0.11
Within archaea	0.07	0.15	0.03	0.09
Between eukaryota-Other	0.11	0.22	0.05	0.12

Table S18: Average divergence of standardized protein length distributions. Average divergence within and between domains for standardized protein length distributions (non-transformed and logarithmic data); using either the Kolmogorov-Smirnov statistics or the Jensen-Shannon distance as a metric.

ANOSIM results

Distance metric / H1	Kolmogorov-Smirnov	Jensen-Shannon	Kolmogorov -Smirnov (Log Length)	Jensen-Shannon (Log Length)
One group per domain	0.30 (p:0.0001)	0.28 (p:0.0001)	0.23 (p:0.0001)	0.11 (p:0.0001)
Two groups: eukaryota - Other	0.41 (p:0.001)	0.38 (p:0.001)	0.28 (p:0.001)	0.15 (p:0.0001)
Two groups: archaea - bacteria	-0.03 (p:0.875)	-0.04 (p:0.95)	0.06 (p:0.01)	-0.04 (p:0.92)

Table S19: ANOSIM results for tests of different hypotheses on dissimilarity matrices of standardized protein length distributions. ANOSIM R-statistic and associated p-value for test of hypotheses that two groups of species are not closer to each other in the similarity matrix of standardized protein length distributions (non-transformed and logarithmic data); using either the Kolmogorov-Smirnov statistic and Jensen-Shanon distance as a metric. The associated H1 hypothesis is written in the first column.

Gene length

Comparison of standardized gene length distribution gives similar results than comparing gene length distribution. In particular, eukaryotes diverge more highly between themselves and with other species in terms of gene length than in terms of protein length, with the separation of eukaryotes and other species being supported by the similarity matrix.
The higher divergence of Deuterostomes in terms of gene length is still observed with standardized distribution, as well, and significant according to the similarity matrix.

However, the overall divergence of standardized gene length distribution, and higher variation within eukaryotes in particular, are less marked when comparing standardized logarithmic distribution.

Average values

Distance metric / Comparison	Kolmogorov-Smirnov	Jensen-Shannon	Kolmogorov-Smirnov (Log Gene Length)	Jensen-Shannon (Log Gene Length)
Overall	0.12	0.23	0.04	0.12
Within eukaryota	0.22	0.32	0.05	0.13
Within bacteria	0.08	0.18	0.04	0.11
Within archaea	0.07	0.16	0.03	0.09
Between eukaryota-Other	0.18	0.32	0.05	0.13
Between Deuterostomia - other eukaryotes	0.31	0.42	0.05	0.13
Between non-deuterostomia eukaryotes	0.17	0.26	0.05	0.13

Table S20. Average divergence of standardized gene length distributions. Average divergence within and between domains for standardized gene length distributions (non-transformed and logarithmic data); using either the Kolmogorov-Smirnov statistics or the Jensen-Shannon distance as a metric.

ANOSIM results

Distance metric / H1	Kolmogorov-Smirnov	Jensen-Shannon	Kolmogorov-Smirnov (Log Length)	Jensen-Shannon (Log Length)

One group per domain	0.37 (p:0.0001)	0.35 (p:0.0001)	0.21 (p:0.0001)	0.12 (p:0.0001)
Two groups: eukaryotes - Other	0.50 (p:00.001)	0.49 (p:0.0001)	0.26 (p:0.0001)	0.18 (p:0.0001)
Two groups: archaea - bacteria	-0.03 (p:0.9113)	-0.04 (p:0.9671)	0.06 (p:0.088)	-0.03 (0.9188)
Two groups: Deuterostomia - Other	0.61 (p:0.0001)	0.58 (p:0.0001)	0.03 (p:0.0856)	0.08 (p:0.0038)

Table S21. ANOSIM results for tests of different hypotheses on dissimilarity matrices of standardized gene length distributions. ANOSIM R-statistic and associated p-value for test of hypotheses that two groups of species are not closer to each other in the similarity matrix of standardized gene length distributions (non-transformed and logarithmic data); using either the Kolmogrov-Smirnov statistic and Jensen-Shanon distance as a metric. The associated H1 hypothesis is written in the first column.

GC content

GC content distribution, in our main analysis, is marked by a high divergence within and between domains, which is mainly contributed by change in the location of distribution. When comparing standardized distribution, the overall distance between distributions is slightly smaller than within protein length distributions showing the shape of GC distribution is also stable across life. As with protein length, we observe that the division between eukaryotes and prokaryotes is well supported by the similarity matrix, as is the division between archaea and bacteria, although more weakly so.

Average values

Distance metric / Comparison	Kolmogorov-Smirnov	Jensen-Shannon
Overall	0.07	0.15
Within eukaryota	0.07	0.14
Within bacteria	0.05	0.13
Within archaea	0.07	0.15
Between eukaryota-Other	0.08	0.18

Table S22: Average divergence of standardized GC content distributions. Average divergence within and between domains for standardized GC content distributions; using either the Kolmogorov-Smirnov statistics or the Jensen-Shannon distance as a metric.

ANOSIM results

Distance metric / H1	Kolmogorov-Smirnov	Jensen-Shannon
One group per domain	0.38 (p:0.0001)	0.38 (p:0.0001)
Two groups: eukaryota - Other	0.44 (p:0.0001)	0.42 (p:0.0001)
Two groups: archaea - bacteria	0.14 (p:0.0001)	0.17 (p:0.0001)

Table S23: ANOSIM results for tests of different hypotheses on dissimilarity matrices of standardized GC content distributions. ANOSIM R-statistic and associated p-value for test of hypotheses that two groups of species are not closer to each other in the similarity matrix of standardized GC content distributions; using either the Kolmogrov-Smirnov statistic and Jensen-Shanon distance as a metric. The associated H1 hypothesis is written in the first column.

Isoelectric point

Finally, the results of comparison of standardized isoelectric distribution is generally similar to the comparisons over the non-transformed distribution. In both cases, we observe a generally low dissimilarity within eukaryotes, and higher divergence within bacteria and especially within archaea.
In this case too, similarity matrices between clades do not favor the distinction between eukaryotes and prokaryotes, but do significantly support a divergence between archaea and bacteria.

Average values

Distance metric / Comparison	Kolmogorov-Smirnov	Jensen-Shannon
Overall	0.11	0.26
Within eukaryota	0.06	0.15
Within bacteria	0.11	0.26
Within archaea	0.18	0.37

Table S24: Average divergence of standardized isoelectric point distributions. Average divergence within and between domains for standardized isoelectric point distributions; using either the Kolmogorov-Smirnov statistics or the Jensen-Shannon distance as a metric.

ANOSIM results

Distance metric / H1	Kolmogorov-Smirnov	Jensen-Shannon
One group per domain	0.09 (p:0.0001)	0.09 (p:0.0001)
Two groups: eukaryota - Other	-0.02 (p:0.92)	-0.01 (p:0.87)
Two groups: archaea - bacteria	0.28 (p:0.0001)	0.26 (p:0.0001)

Table S25: ANOSIM results for tests of different hypotheses on dissimilarity matrices of standardized isoelectric point distributions. ANOSIM R-statistic and associated p-value for test of hypotheses that two groups of species are not closer to each other in the similarity matrix of standardized isoelectric point distributions; using either the Kolmogrov-Smirnov statistic and Jensen-Shanon distance as a metric. The associated H1 hypothesis is written in the first column.

Correlation between genomic features

Within each domain, we computed all pairs of correlations between the three scalar genomic features (median protein length, protein number, genome size). Variation of protein length in regard to the other parameters are mainly uncorrelated (pearson correlation < 0.3). The exception, as noted in a previous study [17], was that median protein length in eukaryotes is inversely correlated to protein number. We find a weak but significant Pearson correlation between these variables (correlation=-0.38, p: 1.9e-18). In order to correct for phylogenetic autocorrelation, we computed Phylogenetic Independent Contrasts for the same three variables (using logarithmic values for proteome and genome size), over a subset of 603 species for which a molecular species tree was available. We tested for correlation over these standardized contrasts. Again, we detected a weak inverse Spearman correlation (correlation=-0.34, p-value: 5e-4) between median protein length and number of proteins in eukaryotes, but also in archaea although with a weaker signal (correlation=-0.30, p-value: 1.4e-2). This indicates that the trend, while weak, is not due to phylogenetic noise.

From our main analysis (see main text : *Protein length is more uniform across species than other genomic features*) distribution of protein length, isoelectric point, and GC content appear to follow different patterns across species. At the gene level, however, correlation between these three distinct features has been noted before [10,4]. To check if this relation could be retrieved at the species level, we computed the Pearson correlation coefficient between all pairs of species' mean protein length, mean GC content and mean isoelectric point for all domains. Only isoelectric point and GC content in archaea were moderately inversely correlated (correlation=-0.54, p-value=6.0e-13), with weaker but statistically significant association in bacteria (correlation=-0.30, p-value: 4.9e-36) and eukaryotes (correlation=-0.18, p-value: 5.3e-05). Protein length was only weakly positively correlated to GC content in bacteria (correlation=0.22, p-value=3.9e-21) and eukaryotes (correlation=0.12, p-value=0.006) though not in archaea, but no significant relation was

found between mean protein length and isoelectric point in any domain. Again, we controlled for phylogenetic signals by computing standardized Phylogenetic Independent Contrasts for these variables and testing for Spearman correlation. The moderate inverse correlation between isoelectric point and GC content was still supported for archaea (correlation=-0.42, p-value: 4.43e-4), but was much weaker in bacteria (correlation=-0.1, p-value: 0.03) and not significant in eukaryotes. The weak relationship between GC content and protein length in eukaryotes (correlation=0.25, p-value:0.01) and bacteria (correlation=0.12, p-value: 9.1e-3) was still supported by this analysis.

Atypical protein length is attributable to weakly supported protein sequence

To better characterize the contributor to atypical protein length, we performed a deeper analysis of the *Drosophila simulans* proteome in our dataset (Figure S41B). When compared to the *Drosophila melanogaster* proteome (Figure S41C), *D. simulans* has a higher proportion of small proteins. As a result, its distribution is more dissimilar to *D. melanogaster* (KS statistics: 0.1) than *D. melanogaster* is to humans (KS statistics: 0.06). Interestingly, this atypical distribution is not found in the latest version of the proteome for *D. simulans* available in RefSeq [27] Figure S41A) which is nearly identical to our *D. melanogaster* (KS statistics: 0.006). The RefSeq annotation pipeline makes use of experimental evidence, including many RNAseq experiments, which add their to their protein coding genes prediction. Given the distribution and the annotation pipeline, our working hypothesis is that the RefSeq *D. simulans* proteome is of much higher quality than our original *D. simulans* dataset.

In this section, we test this hypothesis using expression, sequence features, and conservation. Based on the three lines of evidence, we conclude that the RefSeq *D. simulans* is indeed of much higher quality, and that sequences only annotated in our original *D. simulans* dataset are overwhelmingly artefactual.

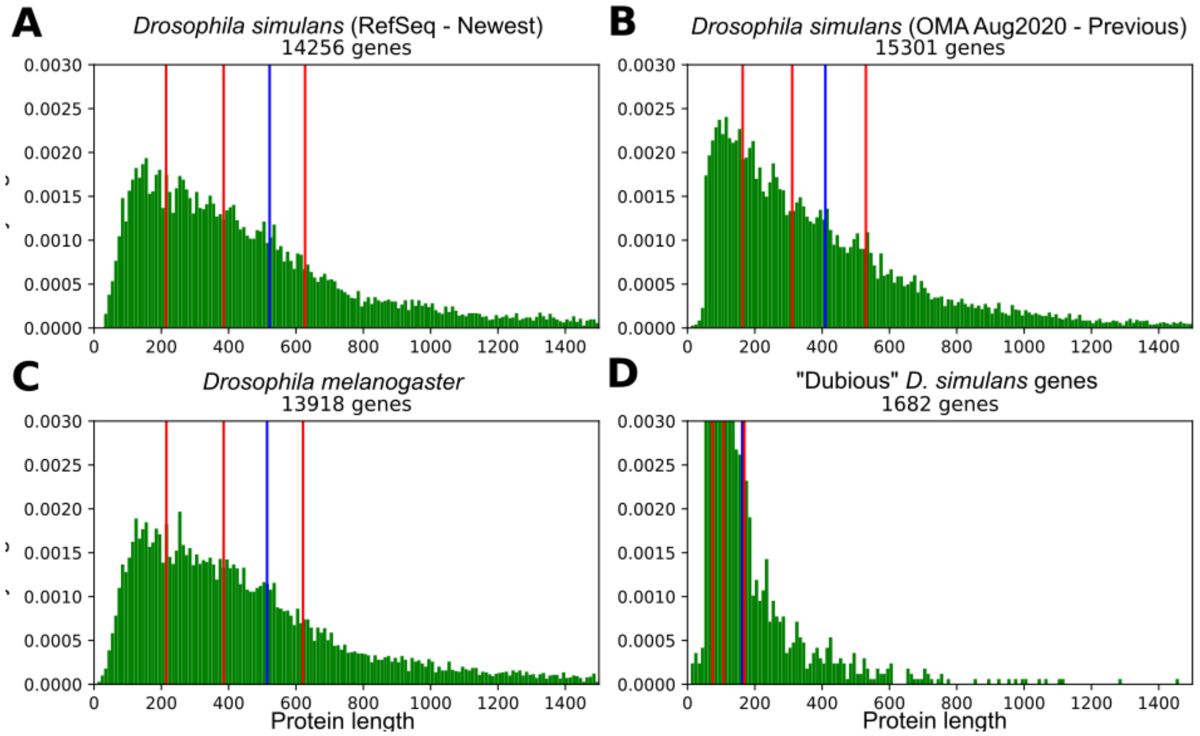

Figure S41: Atypical protein length distribution of *Drosophila simulans* compared to other annotations. All plots show the density of protein length in the dataset, with quartiles shown as vertical red lines and mean as a blue vertical line. A. Protein length distribution for the most recent RefSeq version of *Drosophila simulans.* B. Protein length distribution for the *Drosophila simulans* proteome from our dataset - exported from OMA August 2020 release. C. Protein length distribution of the *Drosophila melanogaster* dataset from our dataset. D. Protein length distribution for proteins from B with no exact match in A.

We used a BLASTP search of all proteins in our current *Drosophila simulans* proteome against the latest RefSeq annotation to identify the proteins with strict sequence correspondence in the newest annotation (percentage identity to closest hit <90%). 1682 proteins were labeled this way as having no strict hit in the newest proteome. As expected, these proteins had a high bias toward short proteins (Figure S41D, Median length: 107).

We selected 50 of these proteins (“dubious” set) at random for a semi-automatic analysis of their features in order to estimate whether they were likely false positives. To avoid our analysis being biased purely by an overrepresentation of small proteins, we also selected a control set of 50 proteins from our proteome with an exact match in the RefSeq proteome. The control set was chosen so each protein had an equivalent of the same size in the ‘dubious’ set (Table S6).

First, we assessed whether there was RNA-expression support for the proteins in either set by querying the Expressed Sequence Tag database for *D. simulans* on the NCBI database. We found support for 18 of the 50 proteins of the “dubious” set, significantly less than for the control (32 over 50, chi-square test P-value: 1.35*10e-3). Of these 18 proteins, 10 of them corresponded to a location of a gene in the current annotation but inside a non-coding region (intron, UTR), a non-coding gene (ncRNA) or as a fragment of an actual CDS. Since identifying such cases requires manual investigations, the presence of EST alone is not a sufficient indicator of protein annotation quality; with this limitation in mind, we tentatively conclude that this “dubious” set likely comprises many artifactual annotations.

Second, we evaluated the sequence features of the proteins in our “dubious” set by querying the UniProt [26] entry of these proteins. 28 out of 50 were annotated as having disordered regions, significantly more than the 15 in the control dataset (chi-square test P-value: 0.015). Accordingly, the structure predicted by AlphaFold [72] for these proteins had a lower average per-residue confidence score (pLLDT) over the proteins than for the control (Figure S42A; Average all sequence pLLDT:51.7 for the ‘dubious set’ against 74.9 for the control set, one-sided Mann-Whitney U test P-value: 2.69*10-8), meaning their protein structure was harder to predict overall. While disordered proteins are known to occur in nature, such a feature is also more likely to occur in randomly occurring sequences than structured protein domains which have more constraint.

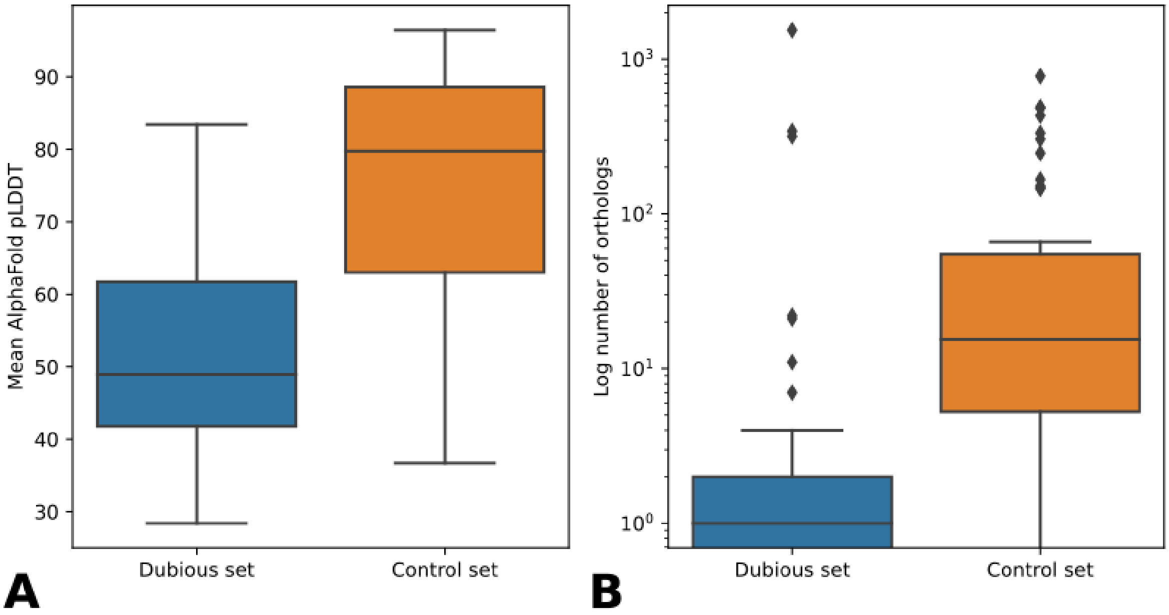

Figure S42. Dubious proteins have less confident structure prediction and less predicted orthologs. A. Boxplot of the mean per residue confidence score for structures predicted by AlphaFold for the dubious and the control set. B. Boxplot of the numbers of orthologs predicted by OMA for protein in the dubious and the control set. The y axis is a logarithmic scale.

Finally, we also evaluated the conservation of the genes across species. To do this we queried the OMA database orthology data for the genes, and counted the number of orthologs detected Figure S42B). Again, the average number of orthologs was significantly higher (One-sided Mann-Whitney U test P-value: 2.57*10-10) for the control genes (median: 15.5) than for the “dubious” (median: 1). Specifically, nearly half (24) the genes categorized as dubious had no orthologs and all but 8 had 3 or fewer. In contrast to the “Control” set that had 6 genes with no orthologs and 12 with 3 or fewer. While no detected homology is not proof of annotation error and would for example be expected for *de novo* genes or fast-evolving genes, protein sequences with essential function are expected to be conserved across species. The fact that these “additional” genes that are not carried over to the latest annotation have little evidence of evolutionary conservation adds weight to the hypothesis they do not correspond to actual protein sequences.

Overall, our analysis provides evidence that a bias toward smaller protein in protein length distribution is partly due to erroneously annotated sequences that are less conserved, less expressed and are likely to be annotated as structurally disordered proteins.

Comparisons of outliers proteomes with other annotation sets

For the 24 outlier proteomes with high BUSCO scores (see main text: *Many protein length distribution outliers are explained by quality issues*), we checked UniProt [6] and RefSeq [5] for alternative annotation sets. We were able to retrieve 23 annotation sets from UniProt and 21 from RefSeq, with one species for which no additional annotation set was available in both databases (Table S7). For 6 species, at least one of the alternative distributions was much closer to the typical distribution of the species' domain than the one available in OMA, with an average KS<0.15, and would not have been considered as an outlier in the present analysis. In each of these cases, the proportion of small proteins differed between sets (Figures S11-S35), with higher median protein sizes in the alternative protein sets. The differences are clearly visible in the case of four eukaryotic protein sets (*Amborella trichopoda*, *Acyrthosiphon pisum*, *Brugia malayi, Loa loa* - Figures S25 and S27-S29): the alternative annotation distributions are much closer to a 'canonical' distribution.

No differences between the retrieved annotation sets does not necessarily mean that the annotations are correct. For example, while few differences were found in the available annotation sets we compared for *Daphnia pulex*, a reannotation of the genome [39] showed significant differences between the new and old annotations. However, the annotation sets from UniProt and from our present dataset are based on the older annotations. In the reannotation study, the authors found a total of 18,440 protein-coding genes in their annotations, instead of the 30,097 previously reported, yet both annotations had a similarly high BUSCO completeness score, close to 96%. Discrepancies between the two sets were reported to be mostly due to a high number of small protein-coding genes in the original annotation, that were not retrieved in the new one. This example supports the hypothesis that an excess of small proteins in genome annotations is due to the methodology of genome assembly or annotation rather than biological particularity.

Finally, even in the cases where few differences are found between the original and alternative annotation sets, other species that are closely related to the "outlier" species have a protein length distribution close to the canonical one. In particular, the atypically-distributed set of bacteria proteomes is composed of 9 representatives of the *Rickettsia* genus. This could hint at a taxonomic biological specificity of the genus, however other species of the same genus display a "canonical" distribution (Figure S36). The fact that inconsistency of the protein length distribution is not verified in the whole clade goes against the hypothesis that these uncommon distributions may be explained by the biological specificities.

Functional analysis of proteomes with abundance of long proteins

In contrast to genomes with a relatively high amount of short proteins, three eukaryotic species (the fungal plant pathogen *Ustilago maydis*, and the protozoan obligate parasites

Toxoplasma gondii (strain VEG) and *Hammondia hammondi)* had diverging distributions characterized by a relatively high amount of proteins longer than 500 amino acids and no overrepresentation of small proteins. Few differences in terms of protein number and length distributions were found between our annotation set and those found on UniProt and RefSeq (although none could be found for *Hammondia hammondi*). Other species in Apicomplexa (clade comprising T*oxoplasma gondii* and *Hammondia hammondi*) - in particular from the *Plasmodium* and *Toxoplasma* genus - and in the *Ustilago* genus displayed similar shape of distributions (large tail of long proteins) (Figures S37 and S38), though they were not flagged as outliers in this analysis, likely because their divergence were not as extreme as the aforementioned three.

The possibility of taxonomic-specific biological particularity appears likelier for these species, especially when taking into account that these are all parasitic or pathogenic species. In order to check if the longer proteins were associated with a specific function, we checked the longest protein-coding genes in these genomes for enrichment of Gene Ontology (GO) [73,74] terms (Figures S38-S40). For the background populations, we used two sets of genes: either the entire gene repertoire of a given species, or the entirety of similarly long-size genes in 25 Apicomplexan species. These analyses are complementary since the former informs on functional categories overrepresented in the longest proteins of the genomes, while the latter informs on functional terms that are specific to long genes in the target species.

No enrichment was supported for longer genes in *Hammondia hammondi* using both backgrounds, likely because it only had 115 genes (1.4%) of its genes annotated with GO terms. Significant enrichments were found for *Ustilago maydis* (Figures S38) and *Toxoplasma gondii* (Figure S39) for proteins longer than 1,000 aa, with both backgrounds. However, the enriched GO terms were mostly generic terms. In particular, *Ustilago maydis* genes were highly enriched over many non-specific categories compared to the "all species' long proteins" background, as exemplified by the most enriched terms (GO:0005515 protein binding, GO:0009987 cellular process, etc). This may reflect that the observed abundance of long genes is not tied to a species-specific feature and that genes in these species are longer than their orthologs in other species regardless of function.

In the case of the *Toxoplasma gondii* GO enrichment using all Apicomplexa long proteins as a background, kinase activity seems important, specifically positive regulation of MAP kinase activity (GO:0043406) and myosin light chain kinase activity (GO:0004687). *T. gondii* contains two MAP kinases, one of which has an increased expression under osmotic stress and during parasite life cycle differentiation [75]. This, combined with the finding that MAPK inhibitors can block *T. gondii* replication [76], suggests that MAP kinases are important for parasite proliferation [77]. Furthermore, myosin light chain kinase was shown to play a key role in the apicomplexan 'gliding' motility, a strategy for penetrating host cells [78].

We extended this analysis to other Apicomplexa, that have a similar, yet less marked, abundance of higher-length proteins. Doing this, we found a significant enrichment ($p < 10^{-10}$) on terms pertaining to host-pathogen interactions in *Plasmodium falciparum* (strain D7) for genes longer than 1,000 amino acids, using either background. For example, 'adhesion of symbiont to host' GO:0044406), 'modulation by symbiont of host erythrocyte aggregation' (GO:0020013), or 'rhoptry' (GO:0020008), which is a specialized apicomplexan organelle

important for host invasion [79]. Whilst it is unclear whether it applies to other apicomplexans, these results point to the abundance of long protein-coding genes possibly contributing to the parasitic lifestyle of *Toxoplasma gondii* and *Plasmodium falciparum*. Overall, however it is not conclusive enough to exclude the possibility of annotation artifacts.

Supplementary methods

Divergence between distributions

Jensen-Shannon distance

Discrete distributions of genomic features were used to generate Kernel Density Estimate with the epanechnikov function, using the scikit-learn module (v1.2.1) [80], over an interval covering the space from the minimal value observed over all sample and maximal value observed over all sample. Results of distribution comparisons were dependent on the bandwidth used for this purpose and of the number of observations. An optimal bandwidth *W* was automatically selected using the freedman_diaconis formula

$$W = 2\frac{IQR(x)}{\sqrt[3]{n}}$$

where IQR(x) is the interquartile range of the data and *n* the number of observations (proteins).
Jensen-Shannon distance between densities was then computed using the implementation in the SciPy python library, with parameter base=2.

Log-transformation

Log transformation of protein length and gene length were done using the Numpy python library (v1.23.3) [65].

Standardisation of the distributions

Standardized values of the genomic observations X were obtained according to this formula:

$$z = \frac{X-\mu}{\sigma}$$

where μ was the mean of the sample and σ its standard deviation.

Analysis of atypical protein in *D. simulans*

External data access

The annotation of the *Drosophila simulans* genome was downloaded from the RefSeq website, and correspondence to the version 103 of the annotation. For each gene represented in the FASTA file, one isoform was selected at random and used to generate a non-redundant FASTA file that was used for all analysis.

PDB files were downloaded from the AlphaFold website (https://alphafold.ebi.ac.uk) on 10th January, 2023 and pLDDT were extracted from the BFactor command using the PDBParser module of the BioPython library (v 1.79) [61]. Domain annotations were downloaded from the UniProt API on 10th January, 2023.
Number of orthologs was extracted from the August 2020 release of the OMA database [18].

Identification of annotation-specific proteins

A BLASTP [81] database was created from the sequences in the RefSeq *Drosophila simulans* annotation using Blast+/makeblastdb (v2.12) and default parameters.

A BLASTP search was run against this database using the *Drosophila simulans* proteome extracted from the August 2020 release of the OMA Database. This was run with Blast+/blastp (v2.12) [82], specifying a maximum number of 5 results and a custom output format ("6 qseqid sseqid pident length mismatch gapopen qstart qend sstart send sl evalue bitscore"). All other parameters were kept as default.

The output of the BLAST search was parsed using the SearchIO module of the BioPython library (v 1.79) and categorized proteins in the OMA proteome as shared with RefSeq if the highest hits in the output had a percentage identity higher than 90.

Selection of proteins

50 proteins from the OMA *D. simulans* proteome were selected from the proteins not shared with RefSeq [5] using the function sample from the random module in Python. 50 control proteins were chosen from the proteins shared with RefSeq, searching for proteins of equal size than the one in the first set in the fraction of the OMA proteome shared with RefSeq.

Expression evidence

Evidence of expression was found using TBLASTN [20] on the NCBI Webserver (January 13th, 2023), targeted to the EST database and the *Drosophila simulans* species. A protein was considered as having trace of expression if a hit was found with more than 90% percent identity over a proportion of the sequence higher than 50%.

Proteins from the “non-shared” groups were mapped to the *Drosophila simulans* genome using TBLASTN on the NCBI Web Server (January 13th, 2023) targeted to the RefSeq_genome database and the *Drosophila simulans* species. This corresponds to the GCF_01676395.2 assembly. When only one locus was found with more than 90% identity, location of genomic elements in the current RefSeq annotation was visually assessed on the Genome Browser on the same webserver.

Statistical tests

Significance of the difference in the number of proteins with disordered proteins and EST support between the non-shared proteins and the control was assessed using a ChiSquare test, as implemented in the SciPy python library (v1.9.1) [63].

Significance of a higher number of orthologs and higher average AlphaFold pLDDT score in the control group rather than in the non-shared group was assessed using a one sided Mann-Whitney U test as implemented in the SciPy python library.

Supplementary Figures

Figure S1: Distributions of protein length (X-axis in logarithmic scale), GC content and gene length (X-axis in logarithmic scale), for selected model eukaryotic species (light green), bacterial and archaeal species (blue). Summary statistics are shown as lines at the bottom of distribution: red lines indicate first quartile, median and third quartile, and the blue line indicates the mean.

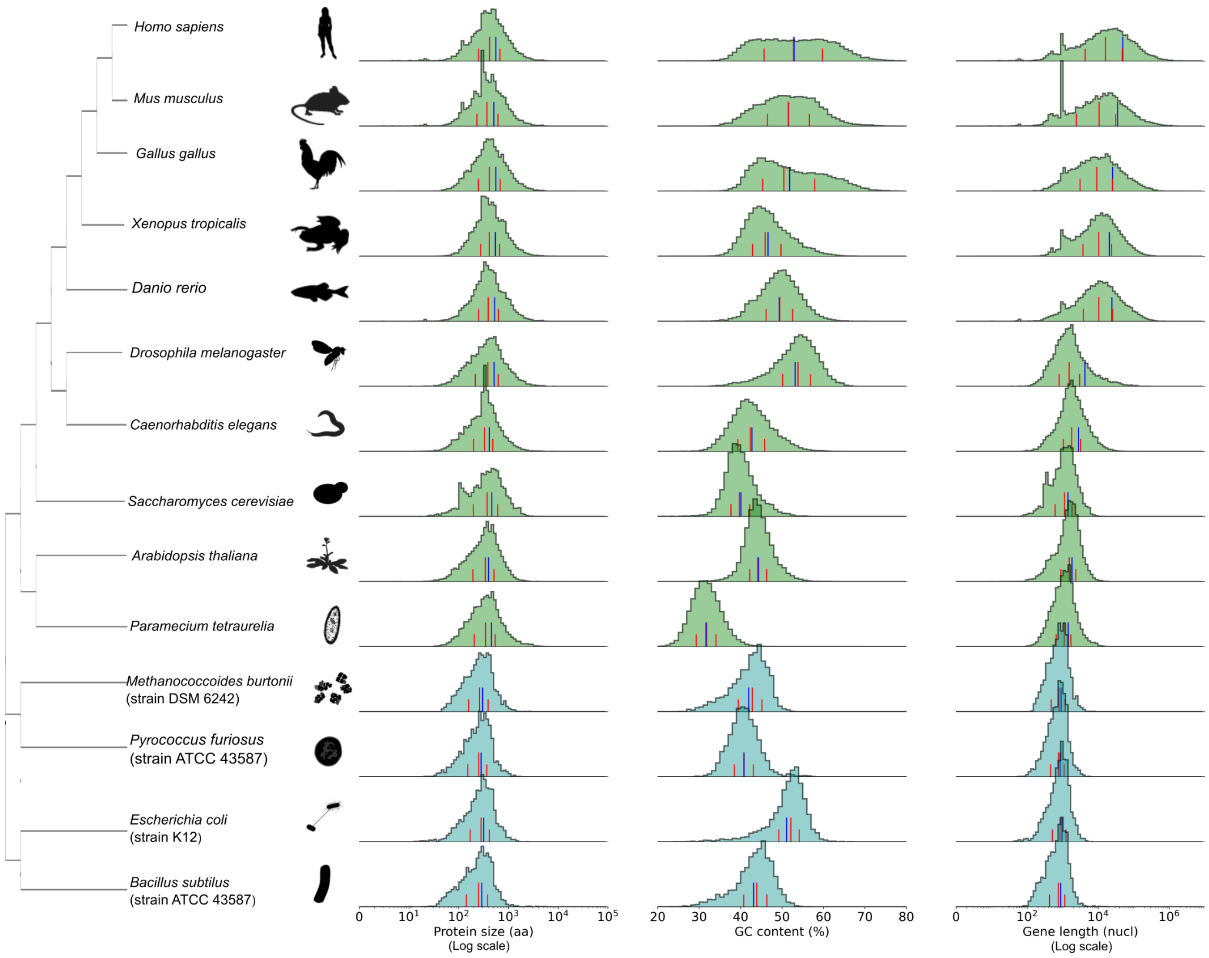

Figure S2: Distribution of the inverse ratio in pairwise comparisons of median protein length (Heatmap in Figure 2a). a. Global. b. By life domain.

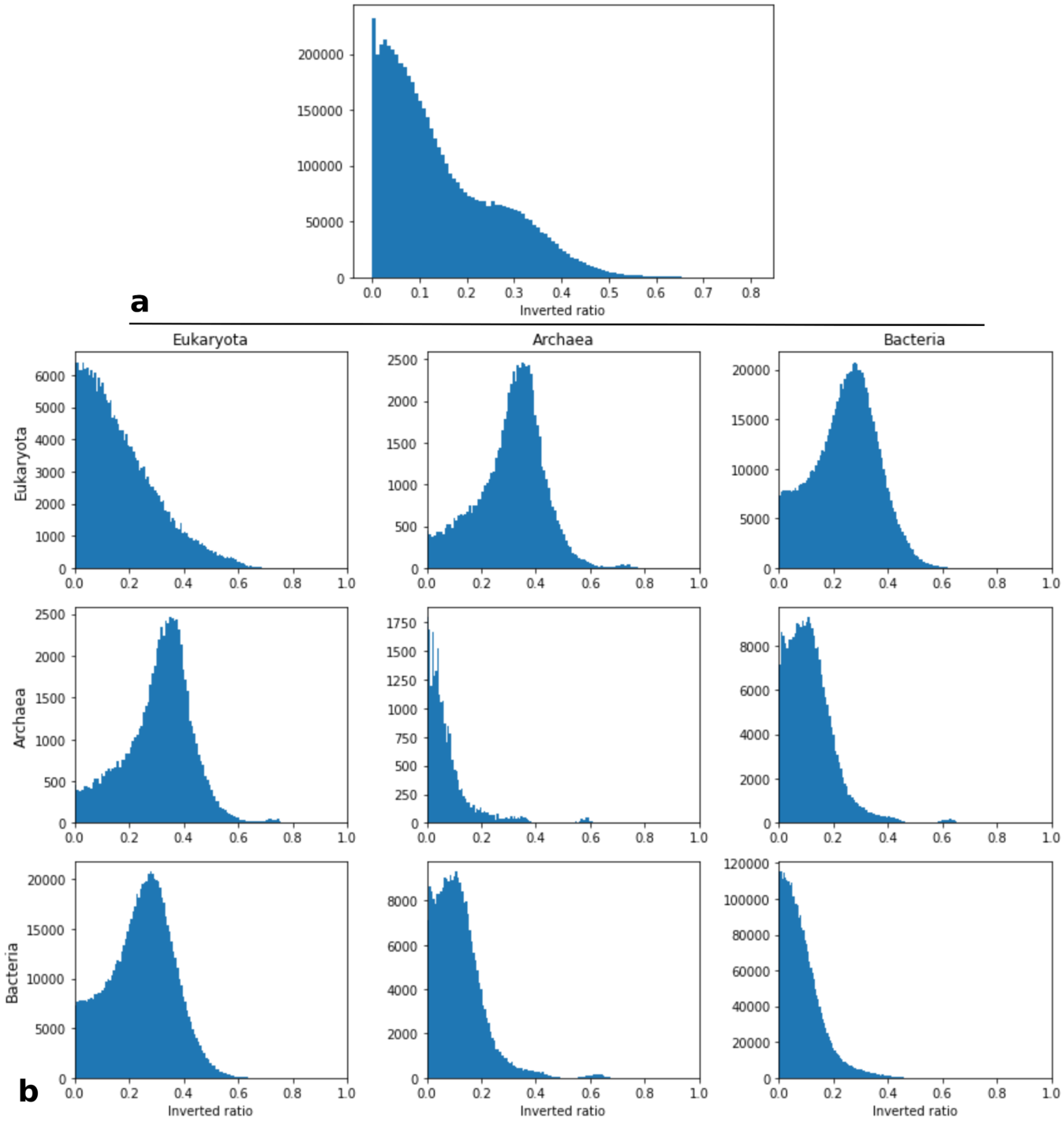

Figure S3: Distribution of the Kolmogorov-Smirnov statistics in pairwise comparisons of protein length distributions (Heatmap in Figure 3b). a. Global b. By life domain.

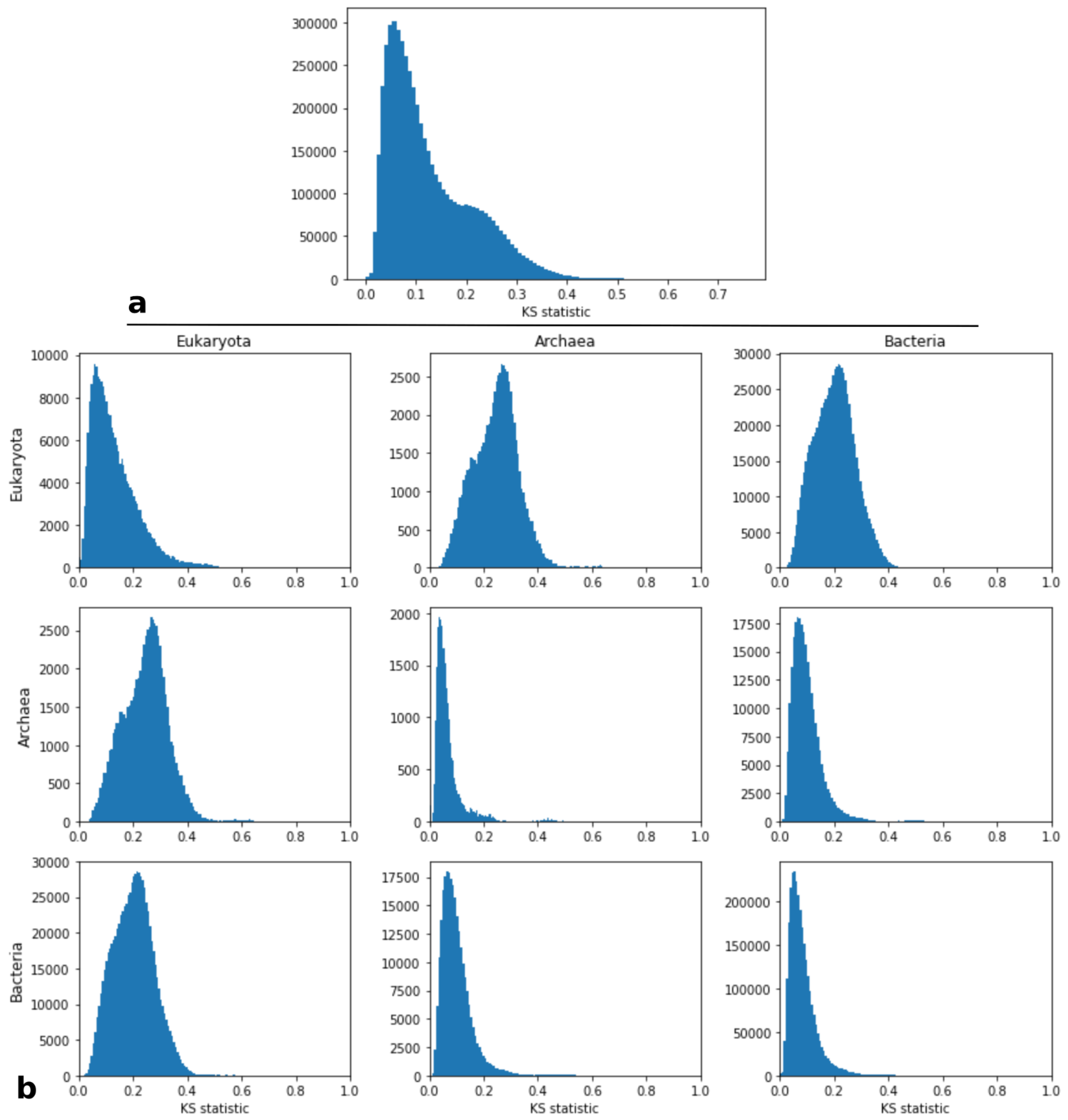

Figure S4: Distribution of the Kolmogorov-Smirnov statistics in pairwise comparisons of number of protein domain distributions (Heatmap in Figure 3b). a. Global b. By life domain.

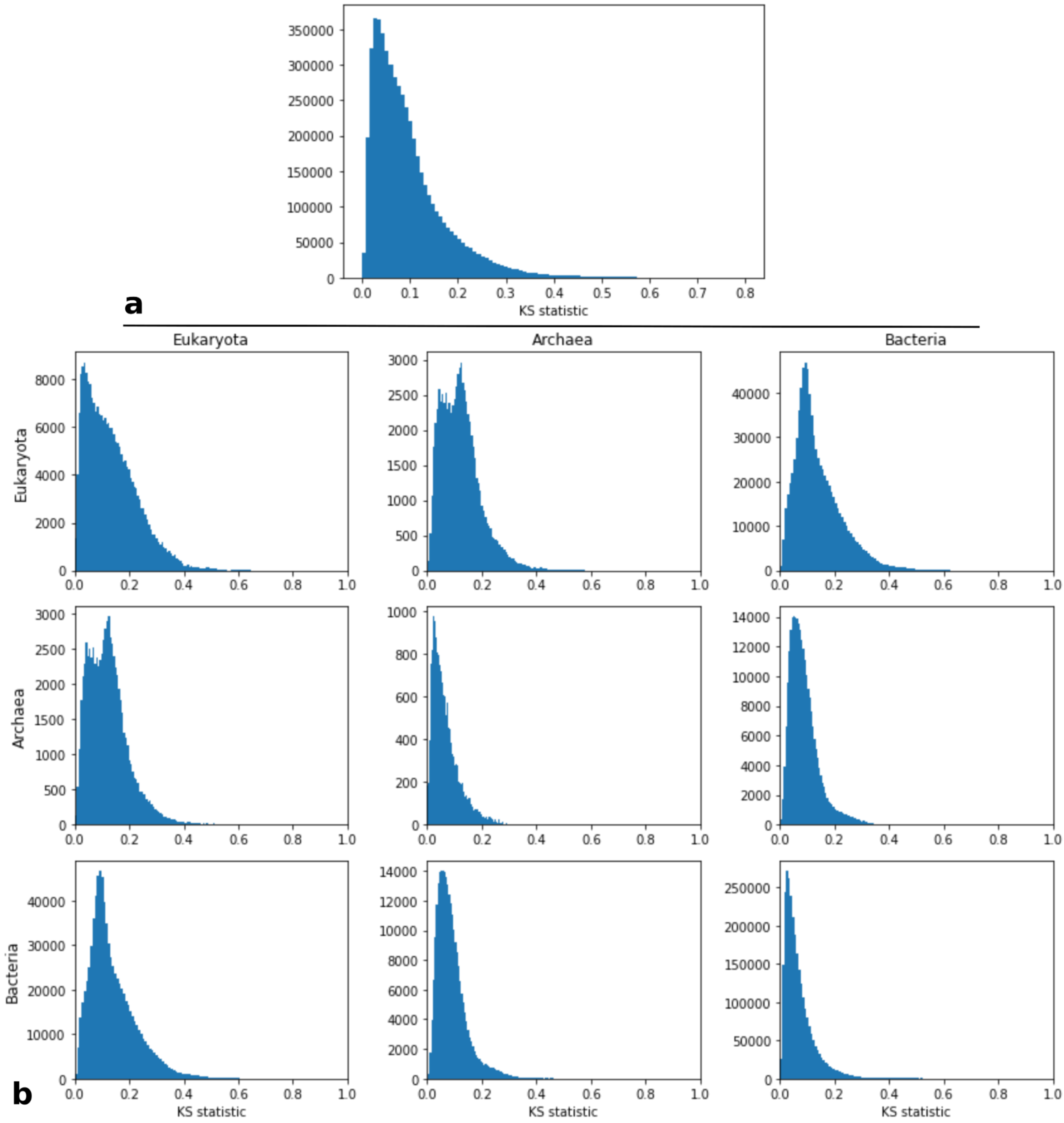

Figure S5: Distribution of the inverse ratio in pairwise comparisons of number of proteins (Heatmap in Figure 3a). a. Global b. By life domain.

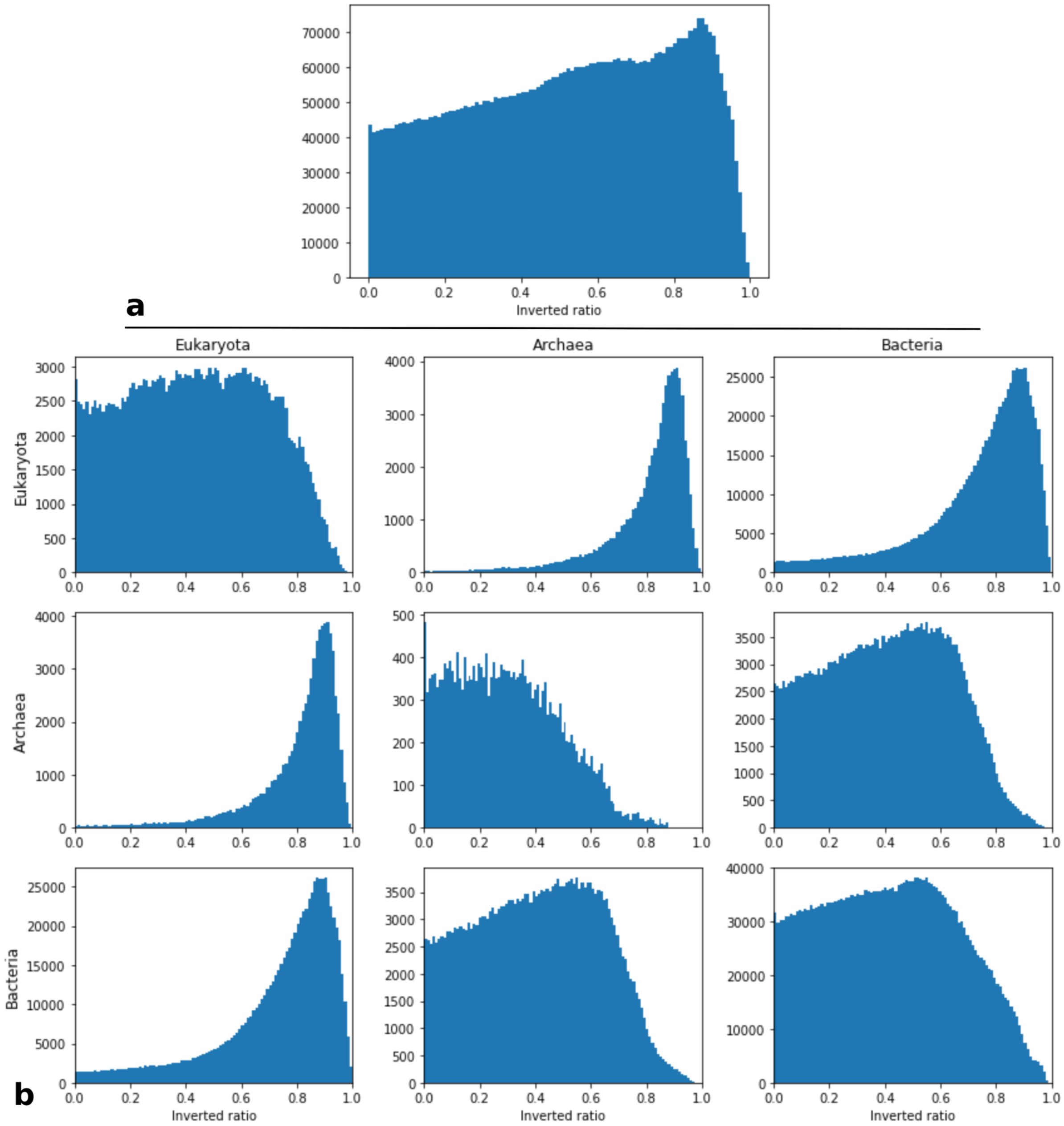

Figure S6: Distribution of the inverse ratio in pairwise comparisons of genome length (Heatmap in Figure 3a). a. Global b. By life domain

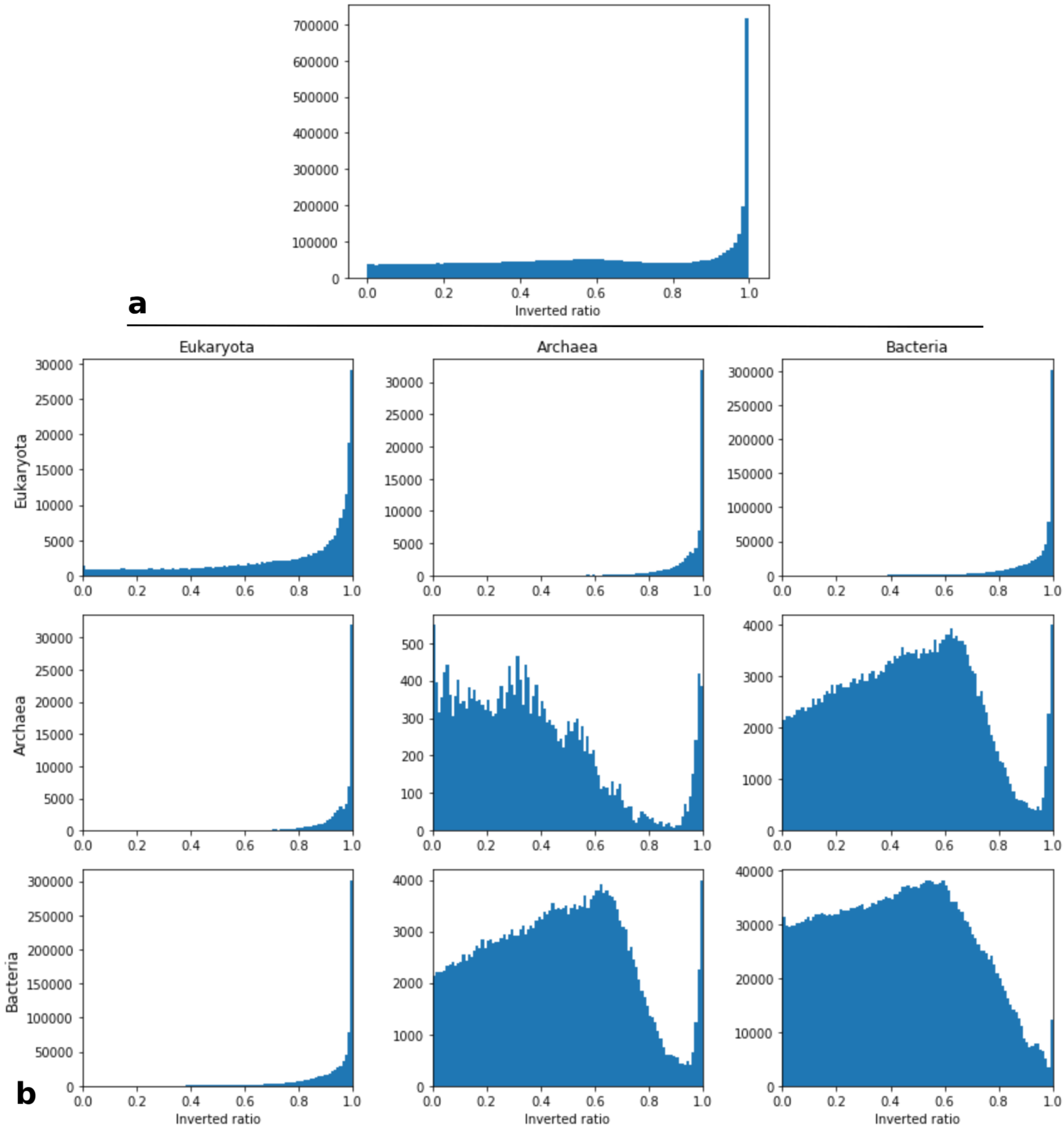

Figure S7: Distribution of the Kolmogorov-Smirnov statistics in pairwise comparisons of gene length distributions (Heatmap in Figure 3b). a. Global b. By life domain.

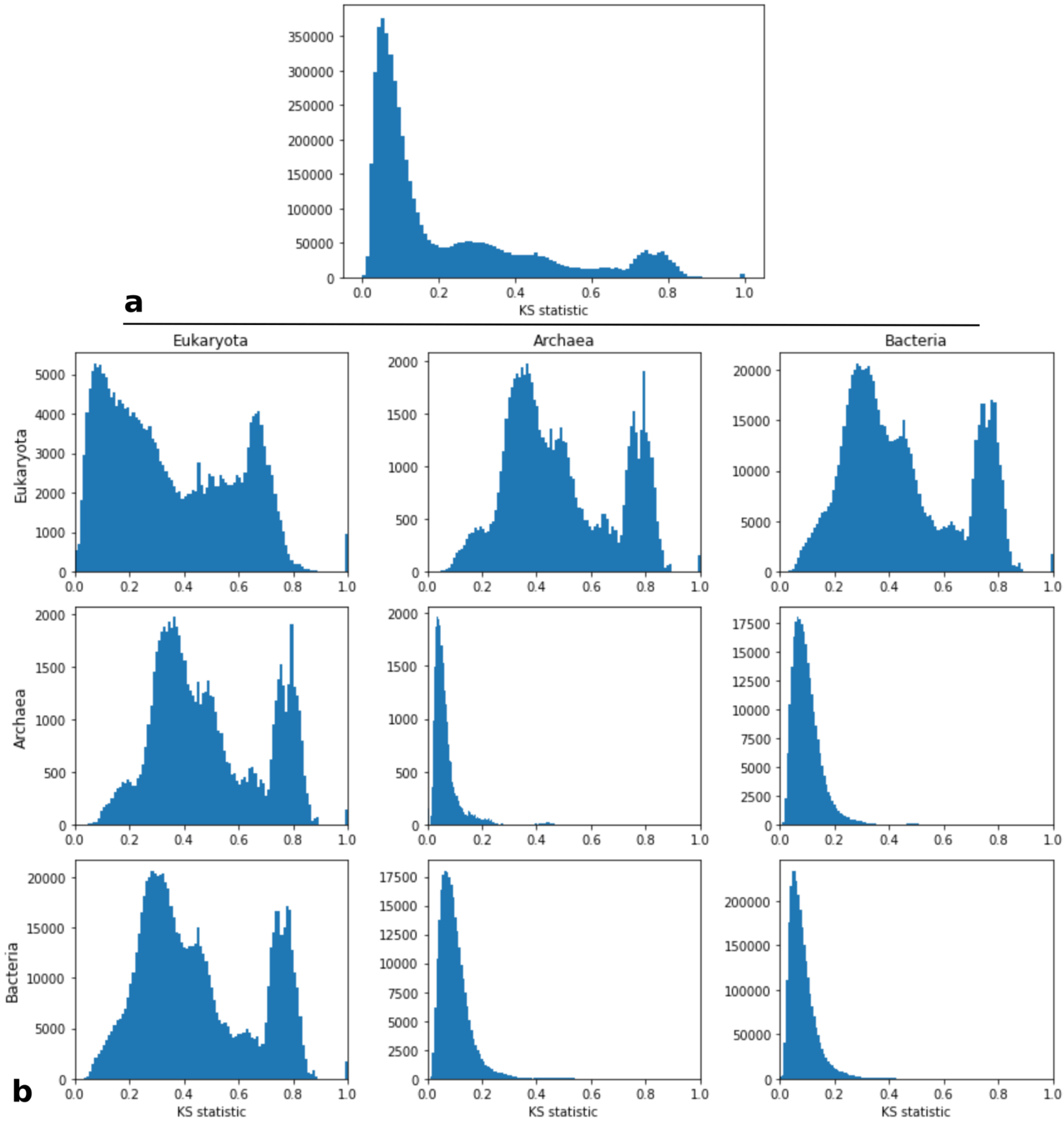

a
KS statistic
b
Eukaryota
Archaea
Bacteria
Eukaryota
Archaea
Bacteria
KS statistic
KS statistic
KS statistic

Figure S8: Distribution of the Kolmogorov-Smirnov statistics in pairwise comparisons of number of gene GC content distribution (Heatmap in Figure 3b). a. Global b. By life domain.

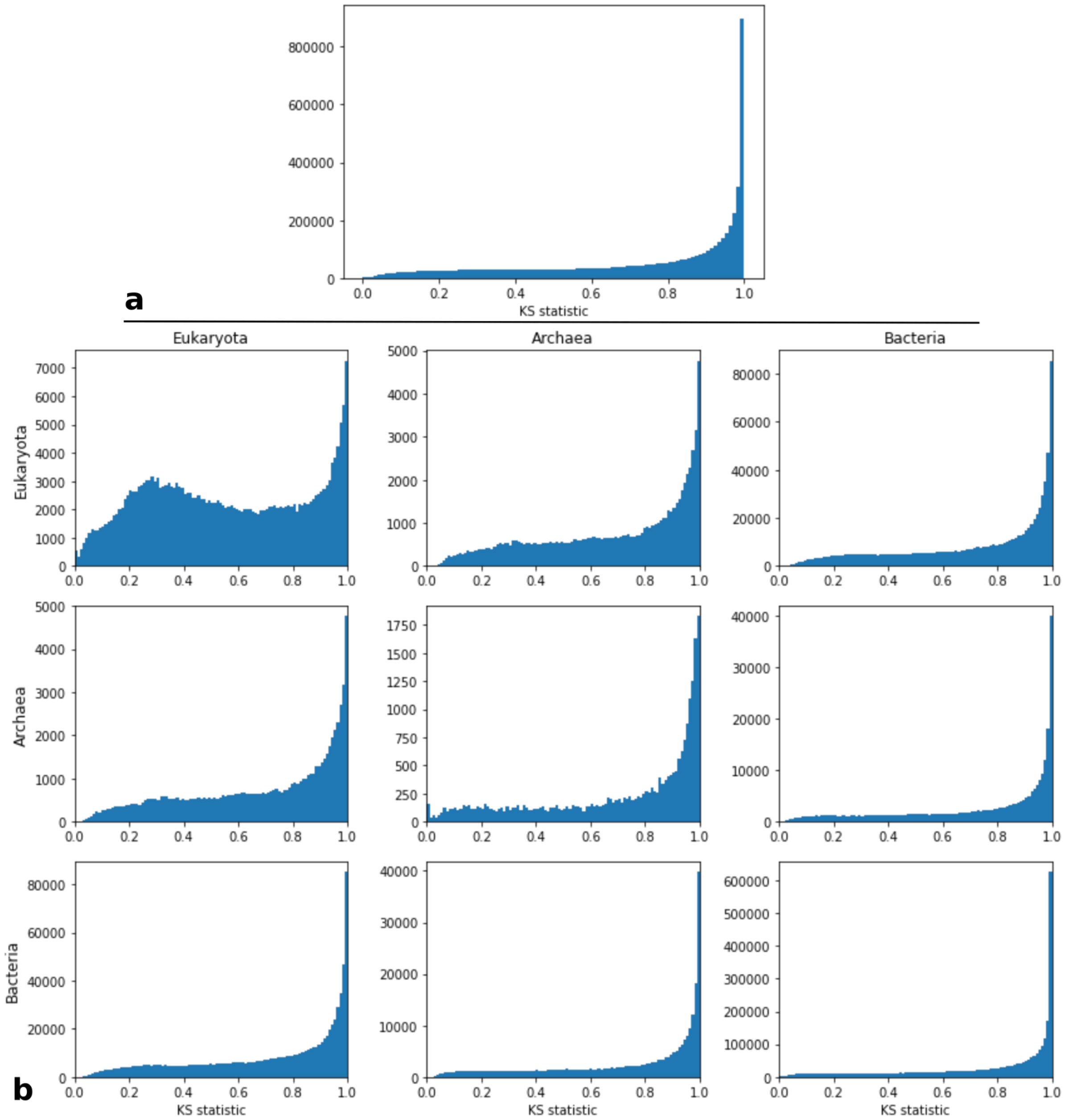

Figure S9: Distribution of the Kolmogorov-Smirnov statistics in pairwise comparisons of number of protein isoelectric point distributions (Heatmap in Figure 3b). a. Global b. By life domain.

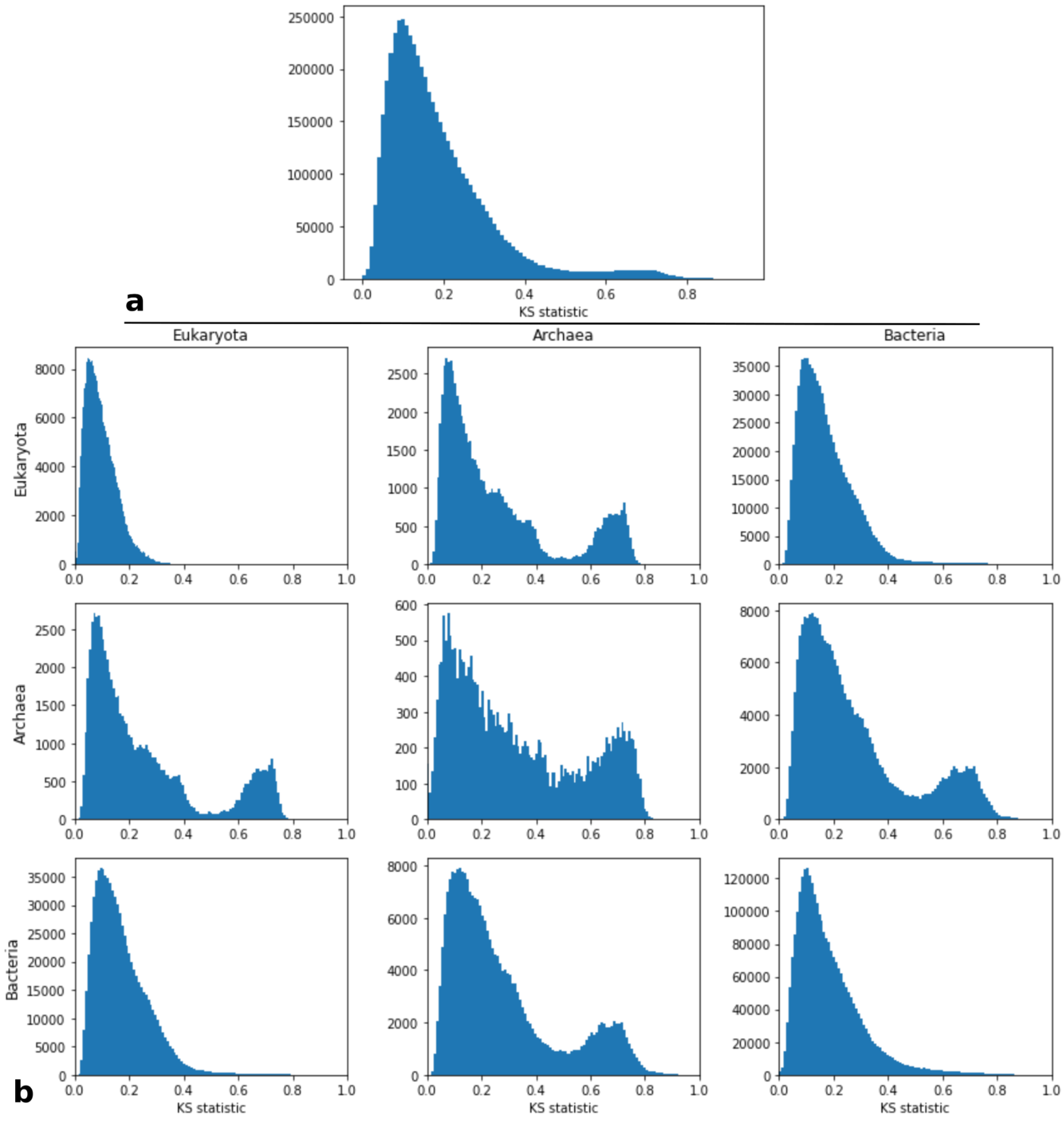

Figure S10: Examples of atypical protein length distributions and distribution heterogeneity between close species. All graphs show the density distribution of protein lengths. The red lines represent the first quartile, median and third quartile of protein lengths, and the blue lines represent the mean. All variables are plotted on logarithmic scale **a-b.**

Examples of proteomes with an overabundance of small proteins (eukaryote *Acyrthosiphon pisum* (pea aphid) (a), and bacteria *Rickettsia rickettsii* (b)) **c**. *Toxoplasma gondii*, an example of a proteome with a high proportion of longer proteins. **d-f**. Example of difference in protein length distributions in the *Drosophila* genus. *Drosophila melanogaster* **(d)** has a canonical protein length distribution shape, and similar distributions exist in other *Drosophila* species like *Drosophila grimshawi* **(e)**. *Drosophila simulans,* however, shows a relative abundance of small proteins **(f)**.

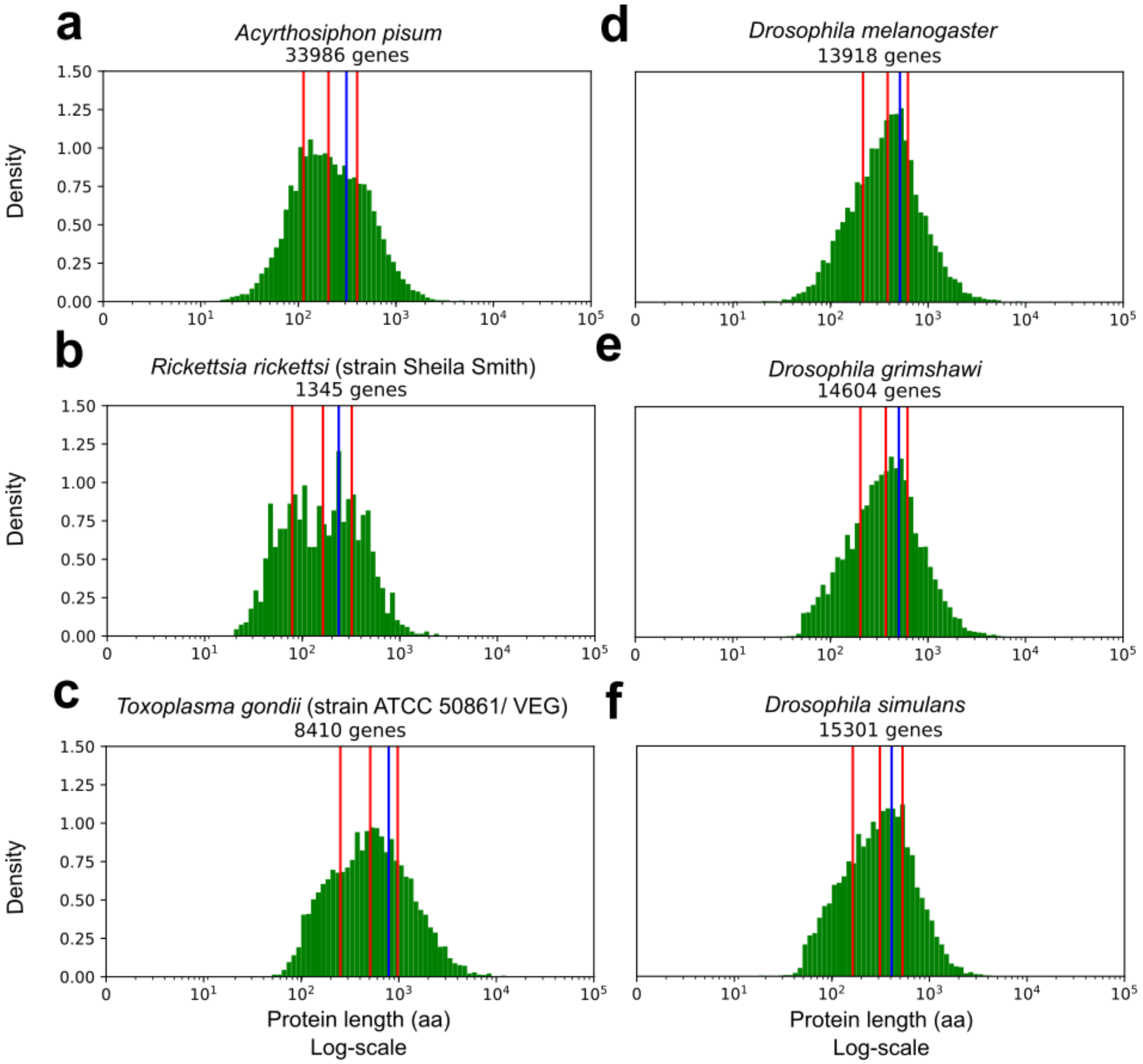

Figures S11-S35: Protein length distribution from different sources for proteomes with atypical distribution, one figure by species. For each outlier species: the protein length distribution of a close species with a canonical distribution (1st from the top), the OMA length

distribution (2nd from the top), and comparison with annotation sets from RefSeq and Uniprot (respectively 3rd and 4th from the top). Missing plots mean no annotation set was found in the corresponding database.

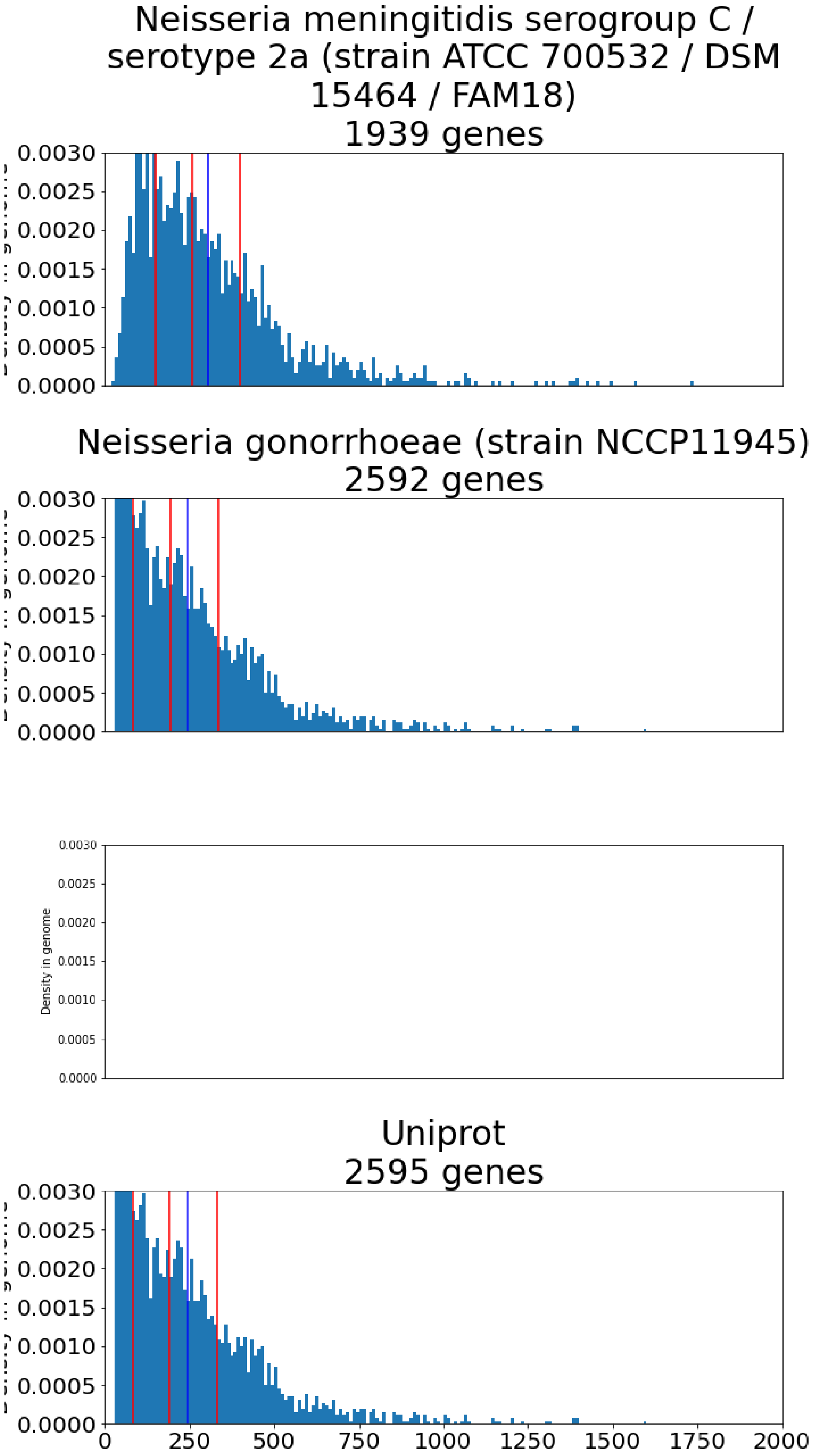
Neisseria meningitidis serogroup C / serotype 2a (strain ATCC 700532 / DSM 15464 / FAM18)
1939 genes
Neisseria gonorrhoeae (strain NCCP11945)
2592 genes
Density in genome
0.0030
0.0025
0.0020
0.0015
0.0010
0.0005
0.0000
Uniprot
2595 genes
0
250
500
750
1000
1250
1500
1750
2000

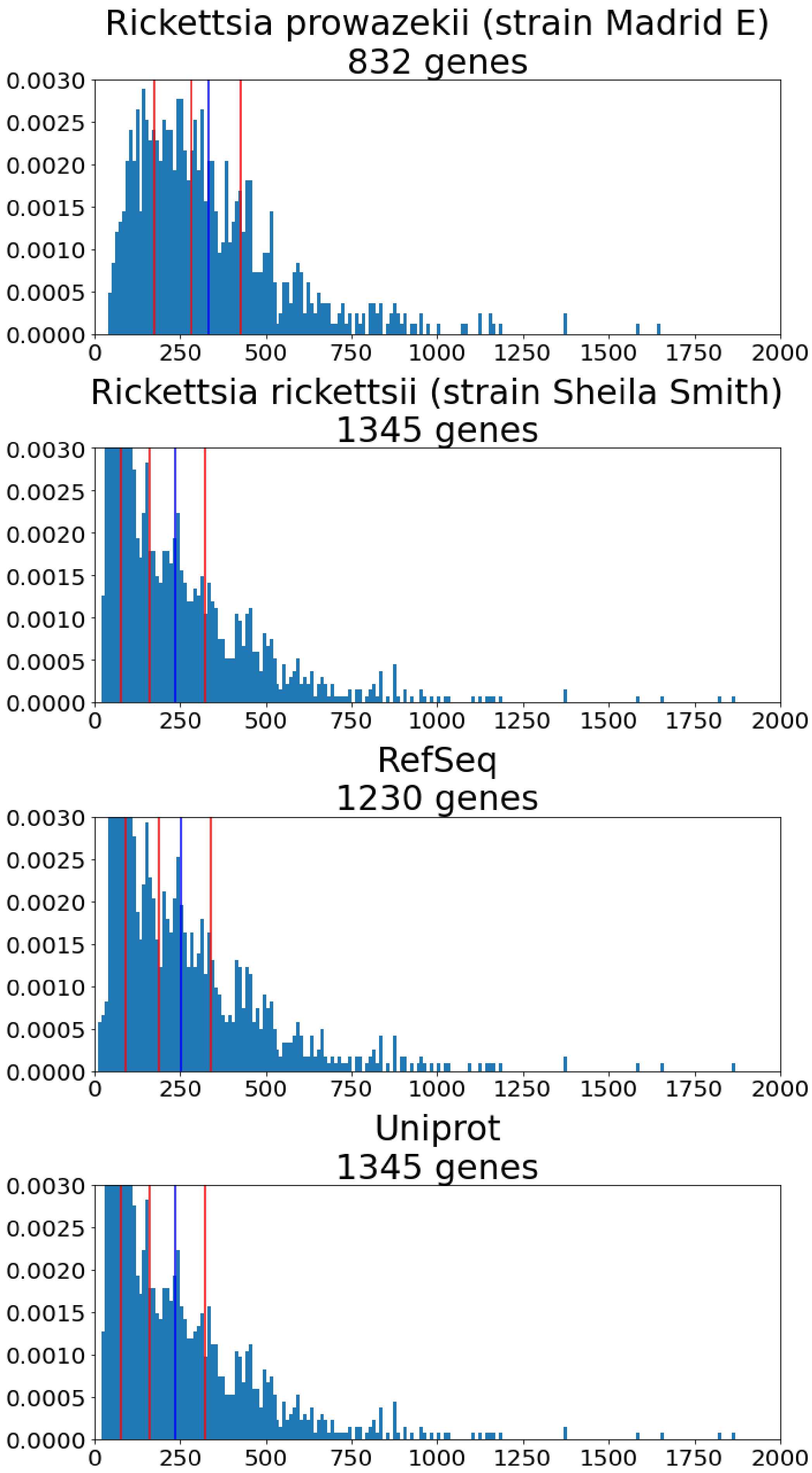
Rickettsia prowazekii (strain Madrid E)
832 genes
Rickettsia rickettsii (strain Sheila Smith)
1345 genes
RefSeq
1230 genes
Uniprot
1345 genes
0.0000
0.0005
0.0010
0.0015
0.0020
0.0025
0.0030
0
250
500
750
1000
1250
1500
1750
2000

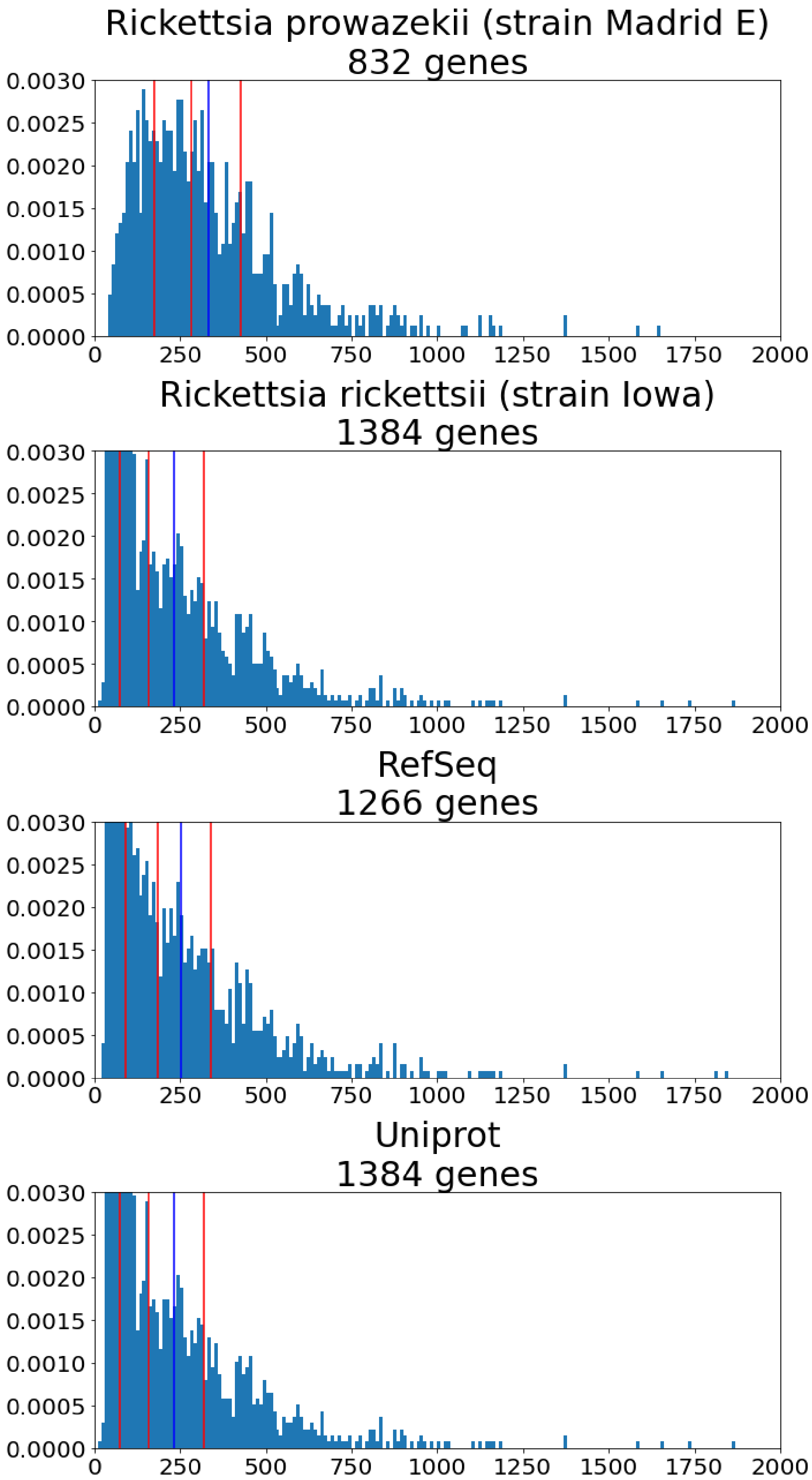
Rickettsia prowazekii (strain Madrid E)
832 genes
Rickettsia rickettsii (strain Iowa)
1384 genes
RefSeq
1266 genes
Uniprot
1384 genes
0.0000 0.0005 0.0010 0.0015 0.0020 0.0025 0.0030
0 250 500 750 1000 1250 1500 1750 2000

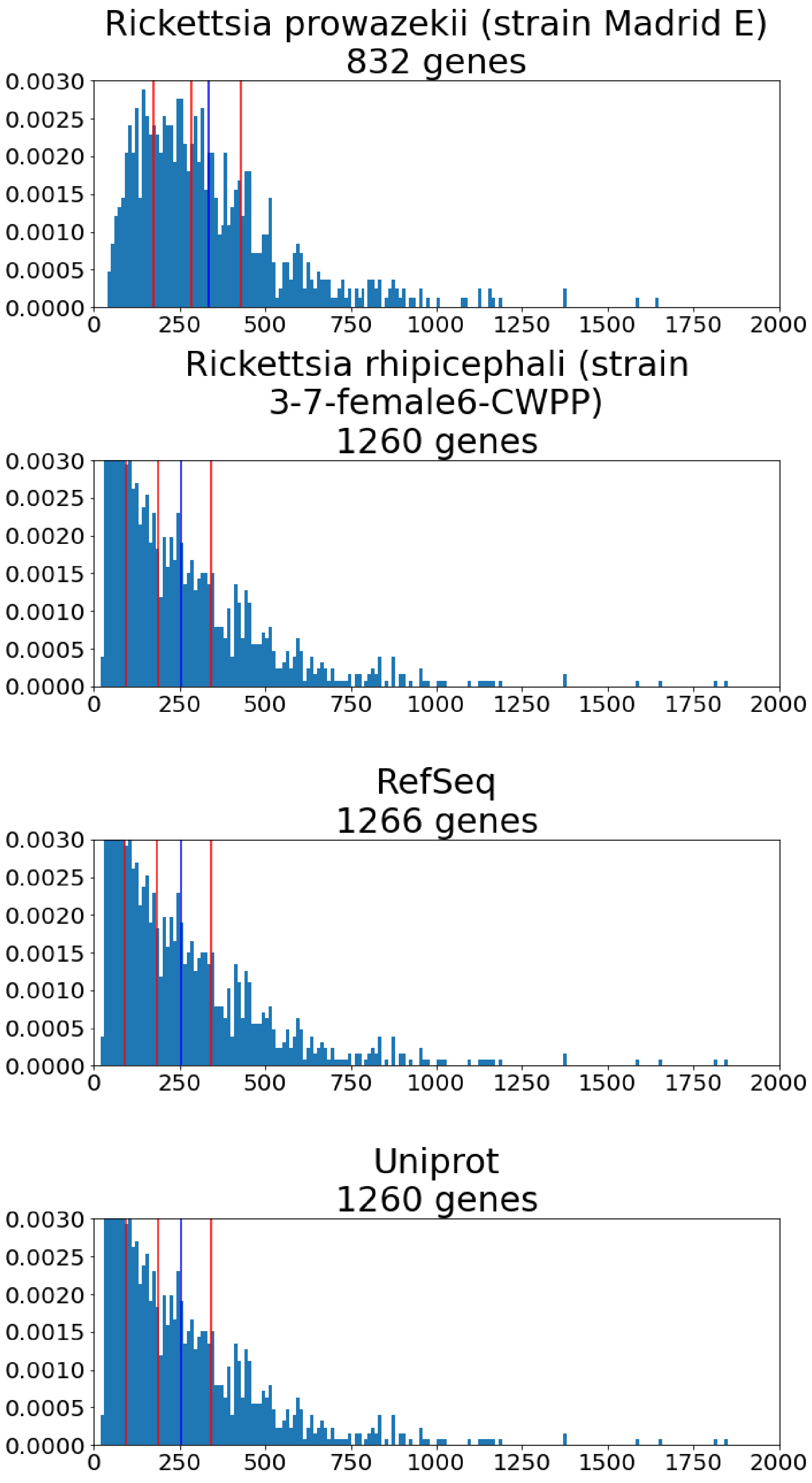
Rickettsia prowazekii (strain Madrid E)
832 genes
Rickettsia rhipicephali (strain
3-7-female6-CWPP)
1260 genes
RefSeq
1266 genes
Uniprot
1260 genes
0.0030
0.0025
0.0020
0.0015
0.0010
0.0005
0.0000
0
250
500
750
1000
1250
1500
1750
2000

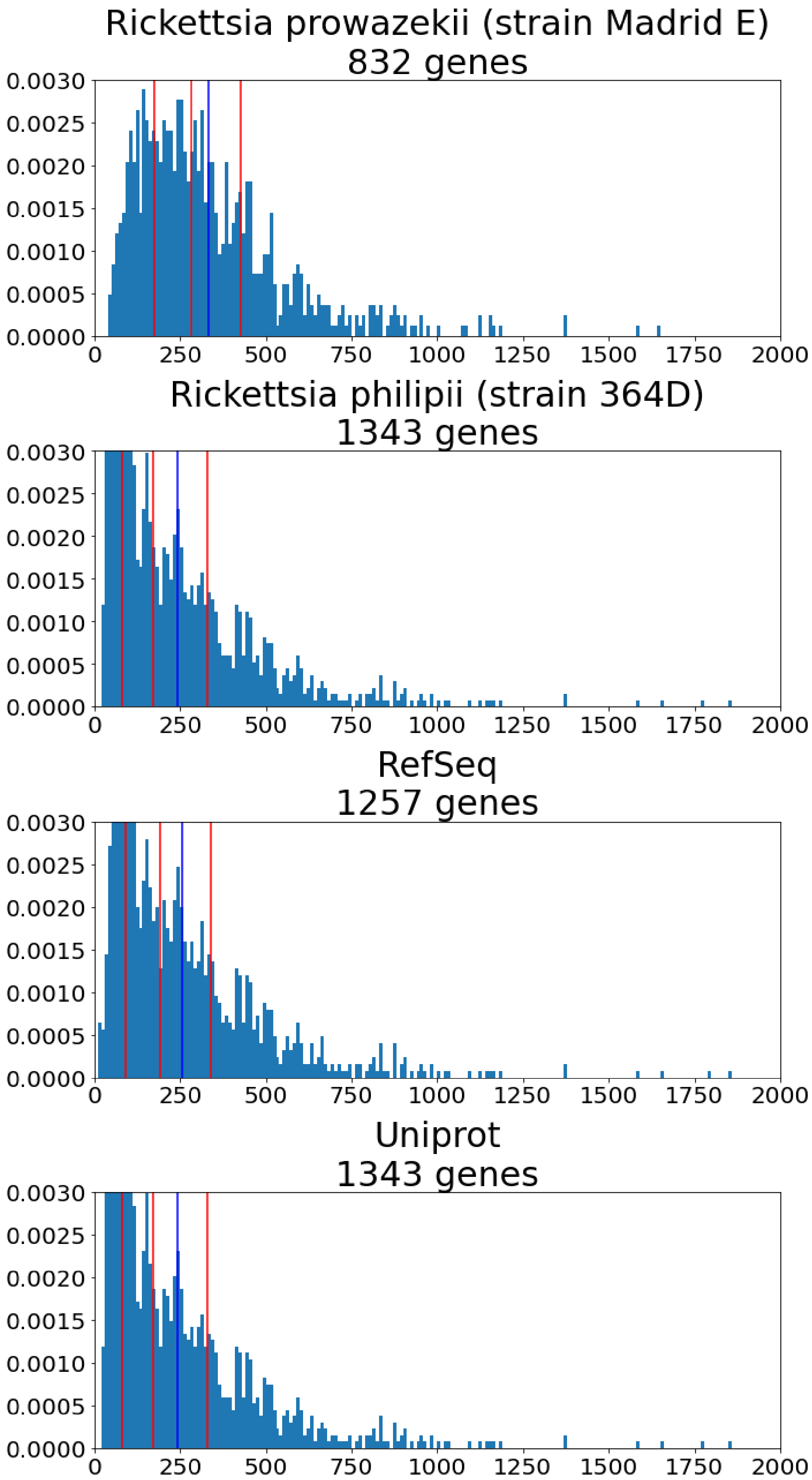
Rickettsia prowazekii (strain Madrid E)
832 genes
Rickettsia philipii (strain 364D)
1343 genes
RefSeq
1257 genes
Uniprot
1343 genes
0.0000
0.0005
0.0010
0.0015
0.0020
0.0025
0.0030
0
250
500
750
1000
1250
1500
1750
2000

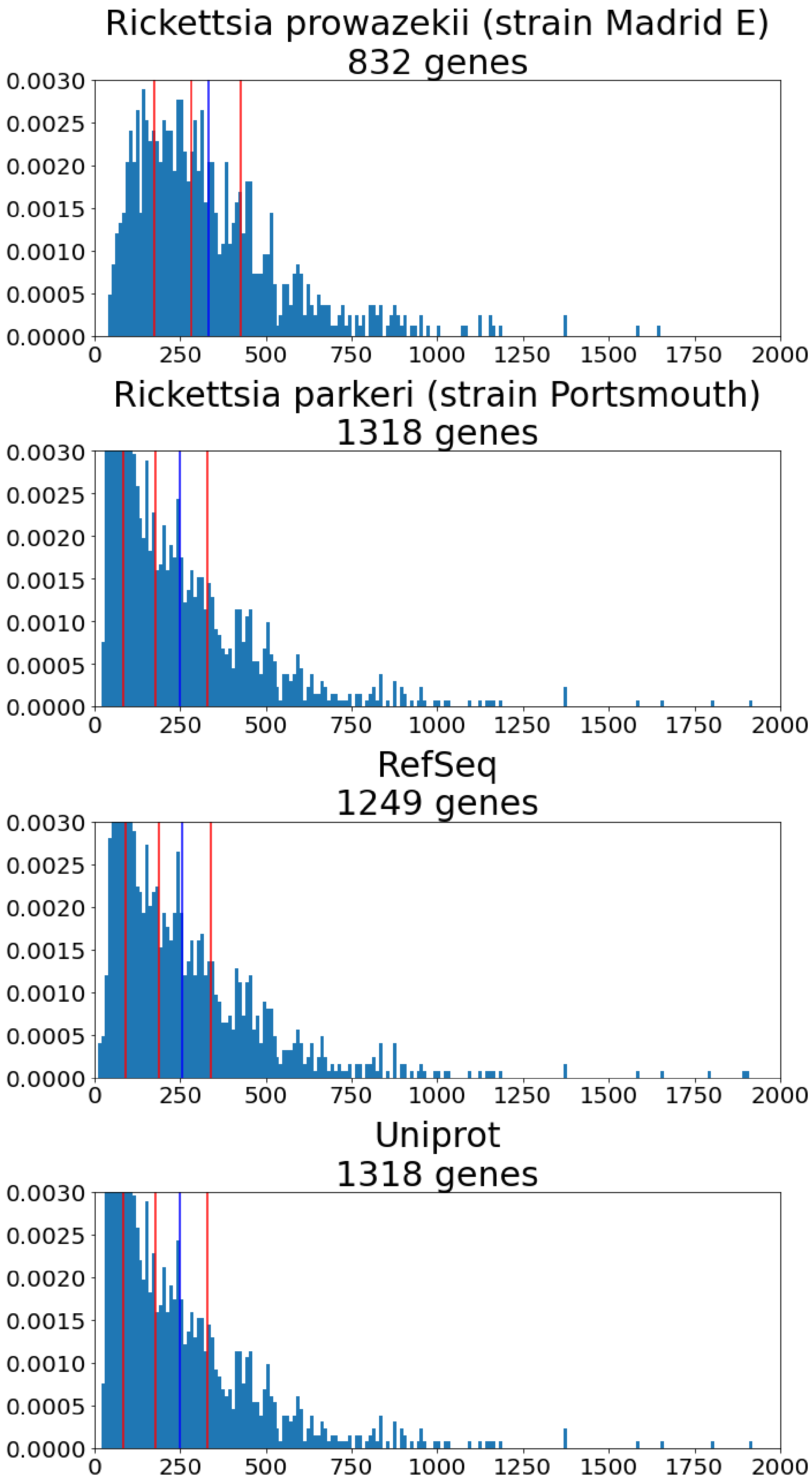
Rickettsia prowazekii (strain Madrid E)
832 genes
Rickettsia parkeri (strain Portsmouth)
1318 genes
RefSeq
1249 genes
Uniprot
1318 genes
0.0000
0.0005
0.0010
0.0015
0.0020
0.0025
0.0030
0
250
500
750
1000
1250
1500
1750
2000

Rickettsia prowazekii (strain Madrid E)
832 genes

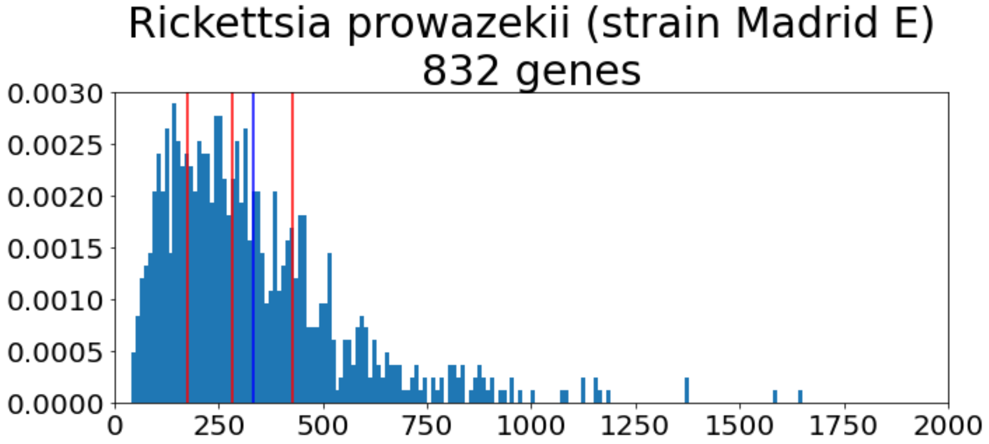

Rickettsia conorii (strain ATCC VR-613 / Malish 7)
1374 genes

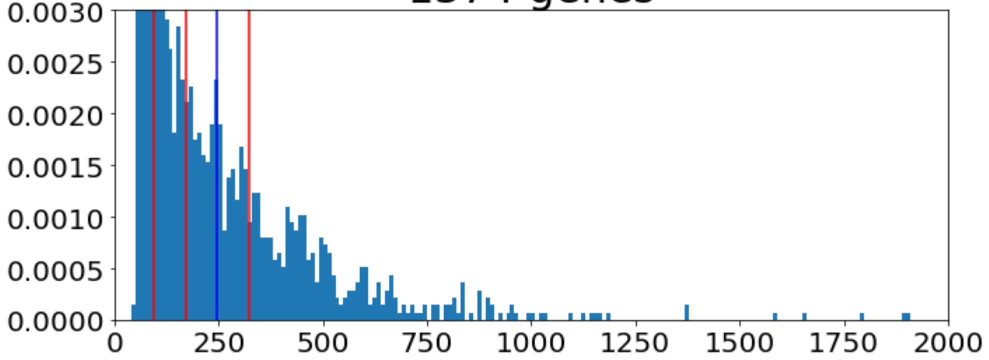

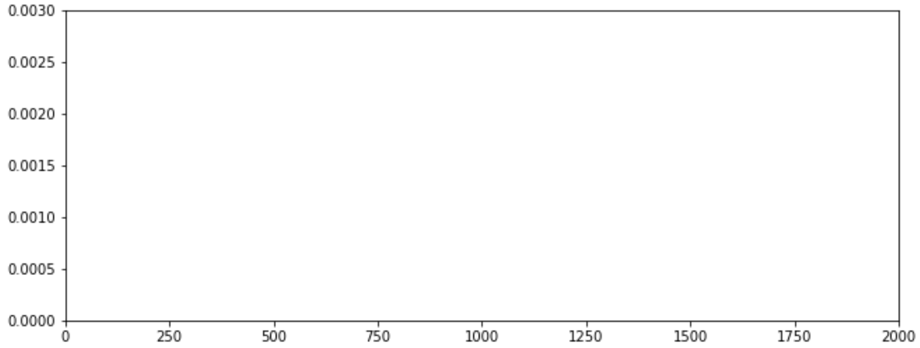

Uniprot
1371 genes

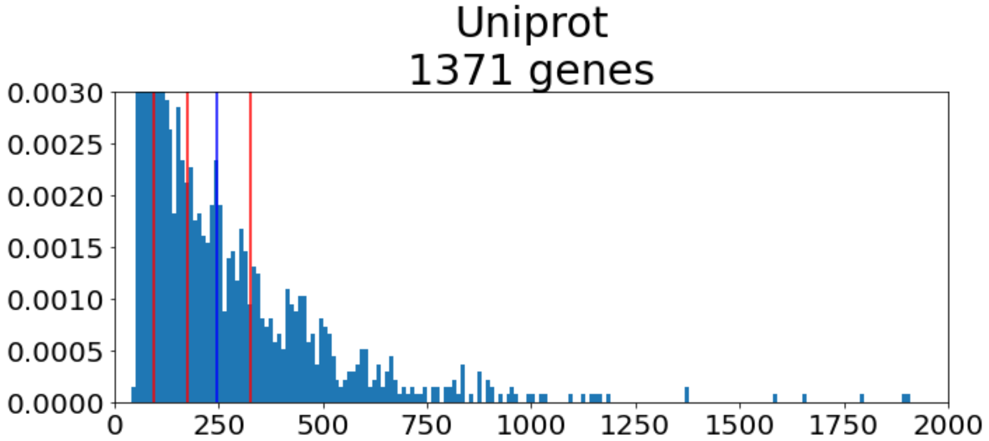

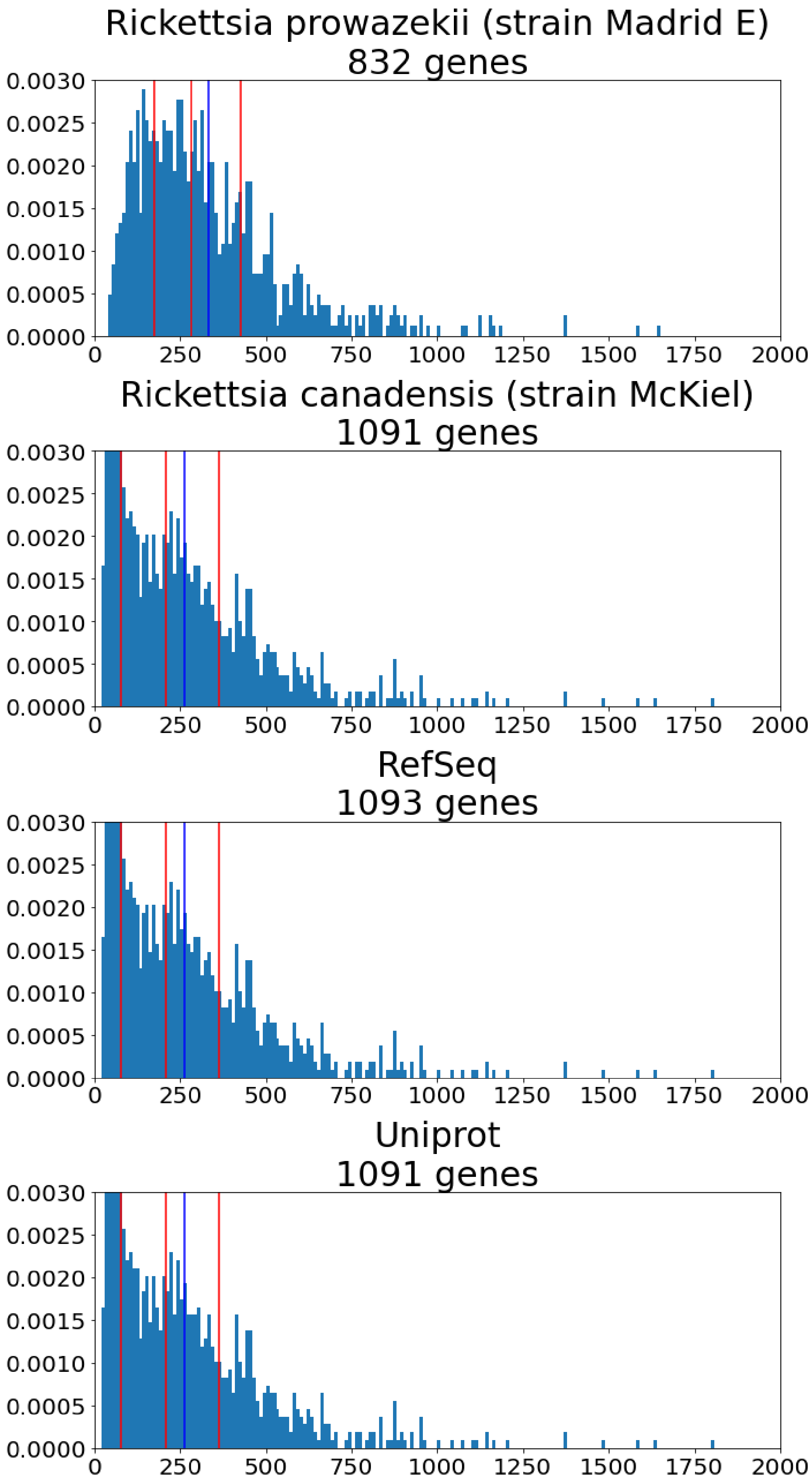
Rickettsia prowazekii (strain Madrid E)
832 genes
Rickettsia canadensis (strain McKiel)
1091 genes
RefSeq
1093 genes
Uniprot
1091 genes
0.0030
0.0025
0.0020
0.0015
0.0010
0.0005
0.0000
0
250
500
750
1000
1250
1500
1750
2000

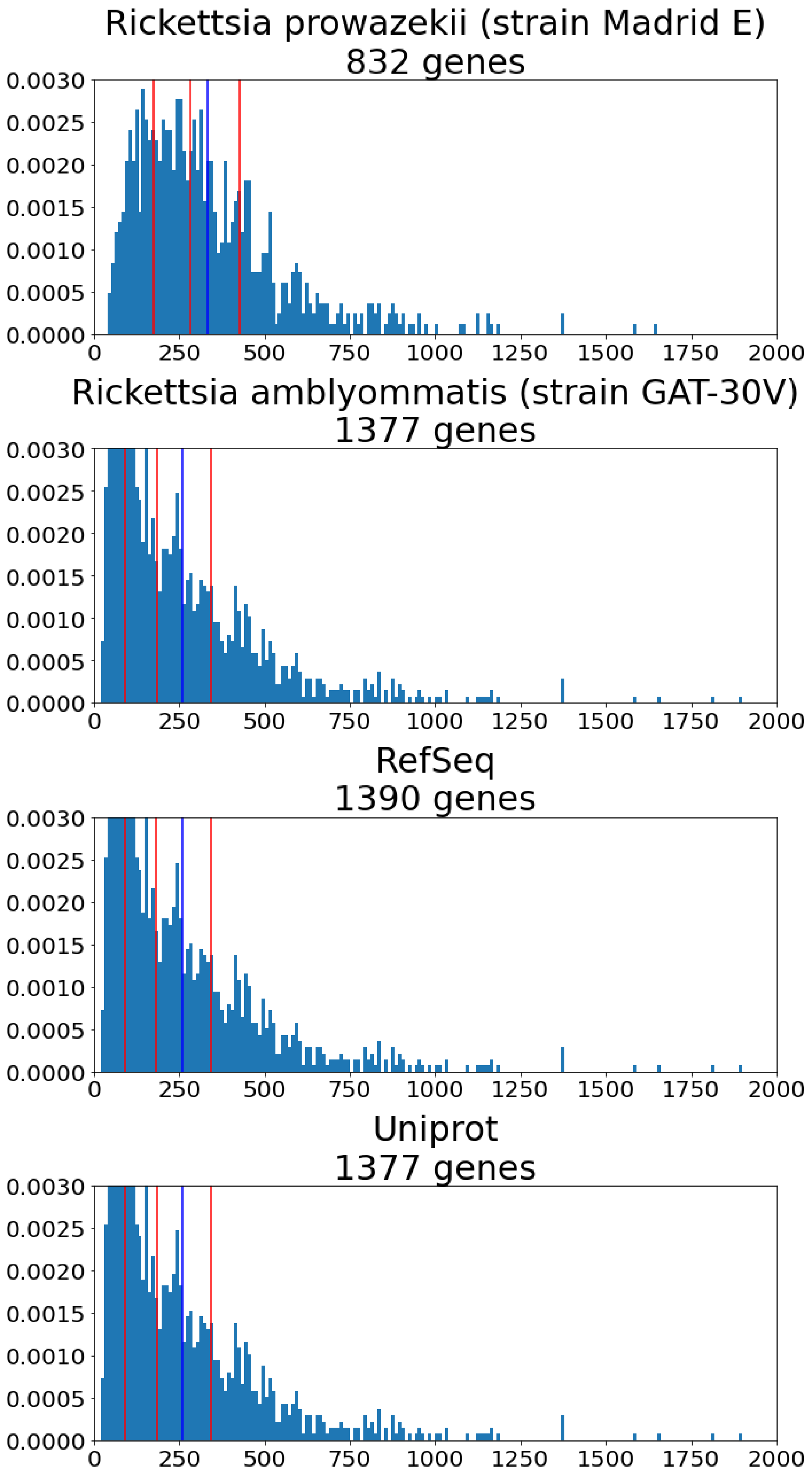
Rickettsia prowazekii (strain Madrid E)
832 genes
Rickettsia amblyommatis (strain GAT-30V)
1377 genes
RefSeq
1390 genes
Uniprot
1377 genes
0.0030
0.0025
0.0020
0.0015
0.0010
0.0005
0.0000
0
250
500
750
1000
1250
1500
1750
2000

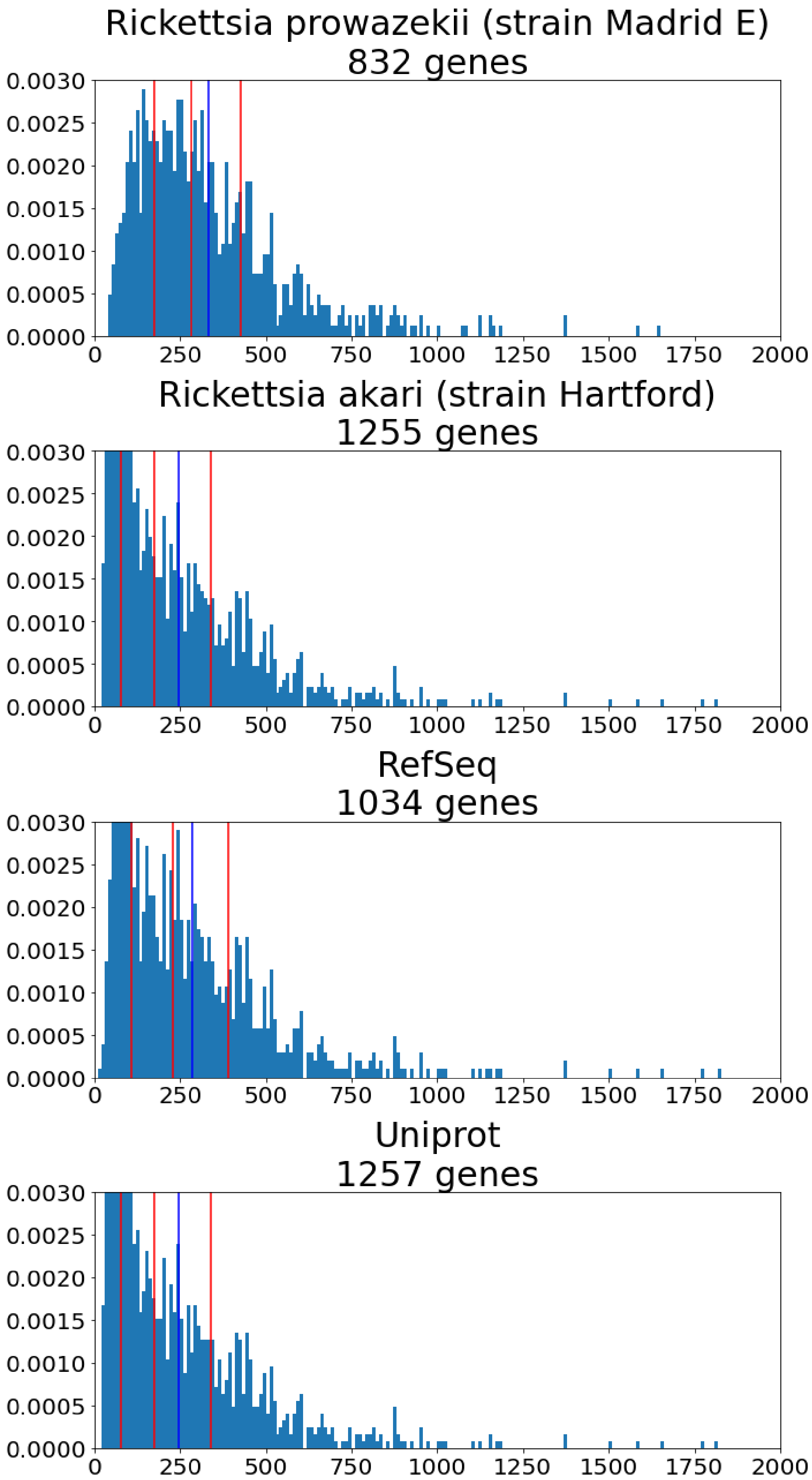
Rickettsia prowazekii (strain Madrid E)
832 genes
Rickettsia akari (strain Hartford)
1255 genes
RefSeq
1034 genes
Uniprot
1257 genes
0.0030
0.0025
0.0020
0.0015
0.0010
0.0005
0.0000
0
250
500
750
1000
1250
1500
1750
2000

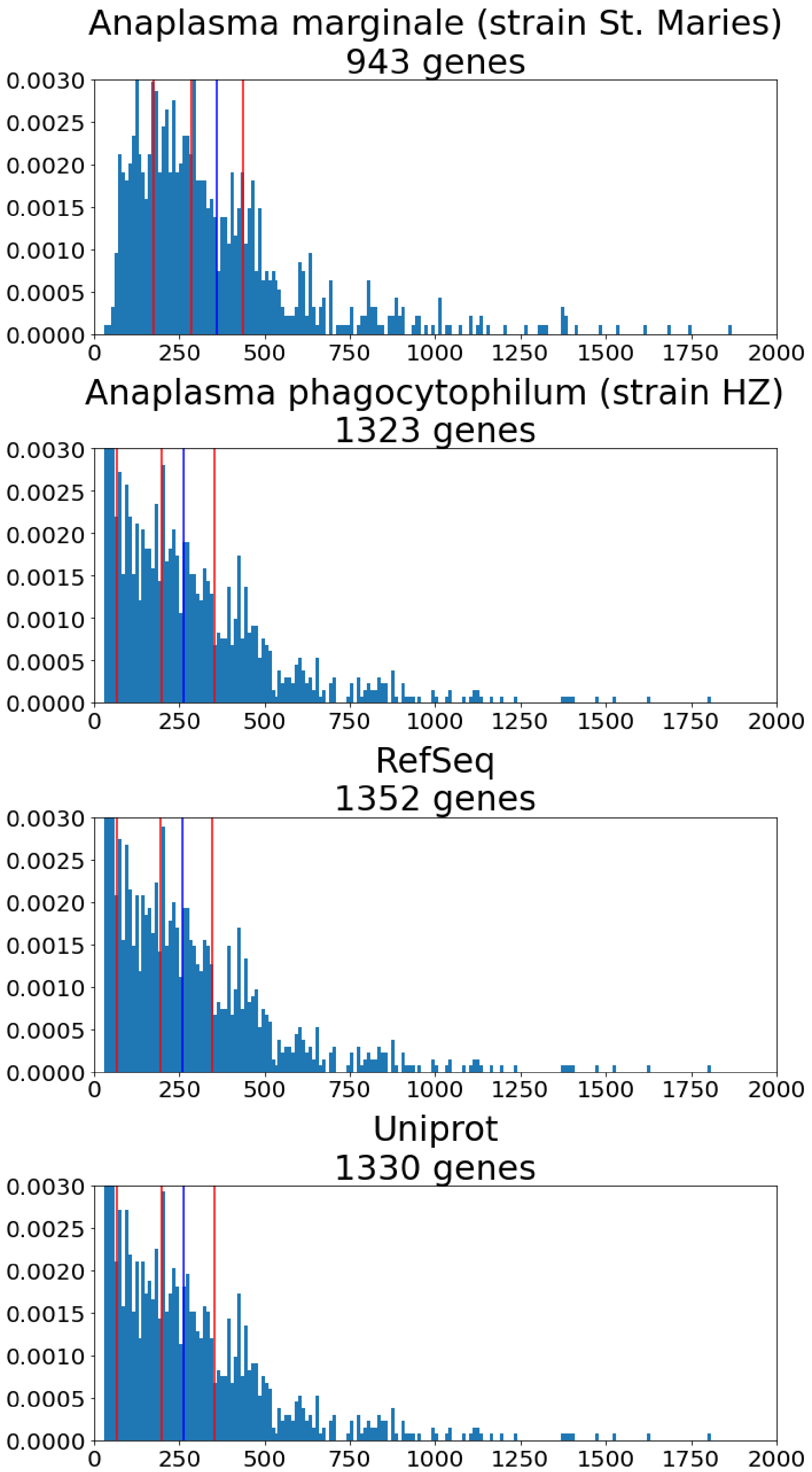
Anaplasma marginale (strain St. Maries)
943 genes
0.0030
0.0025
0.0020
0.0015
0.0010
0.0005
0.0000
0
250
500
750
1000
1250
1500
1750
2000
Anaplasma phagocytophilum (strain HZ)
1323 genes
0.0030
0.0025
0.0020
0.0015
0.0010
0.0005
0.0000
0
250
500
750
1000
1250
1500
1750
2000
RefSeq
1352 genes
0.0030
0.0025
0.0020
0.0015
0.0010
0.0005
0.0000
0
250
500
750
1000
1250
1500
1750
2000
Uniprot
1330 genes
0.0030
0.0025
0.0020
0.0015
0.0010
0.0005
0.0000
0
250
500
750
1000
1250
1500
1750
2000

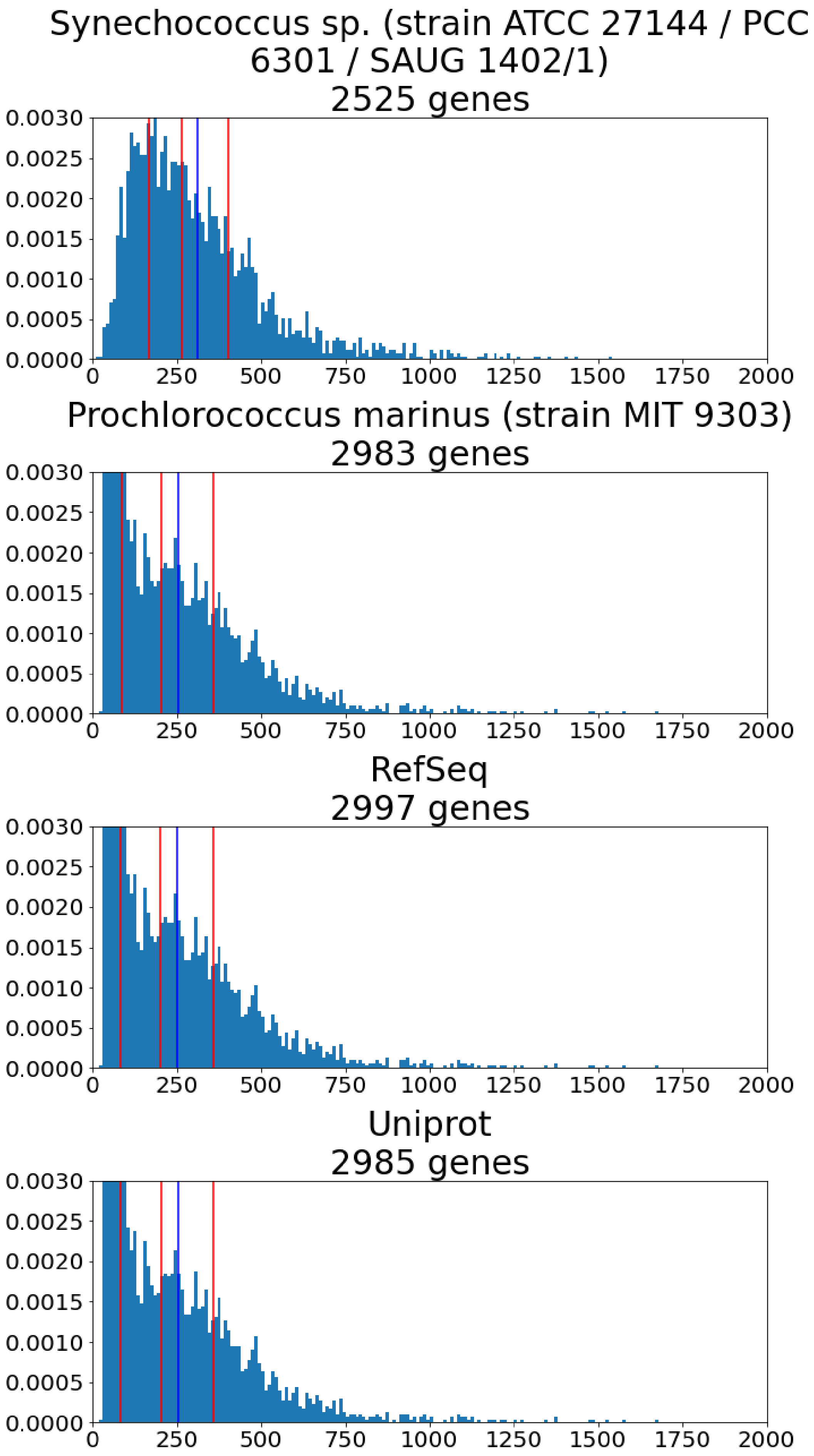
Synechococcus sp. (strain ATCC 27144 / PCC 6301 / SAUG 1402/1)
2525 genes
Prochlorococcus marinus (strain MIT 9303)
2983 genes
RefSeq
2997 genes
Uniprot
2985 genes
0.0000
0.0005
0.0010
0.0015
0.0020
0.0025
0.0030
0
250
500
750
1000
1250
1500
1750
2000

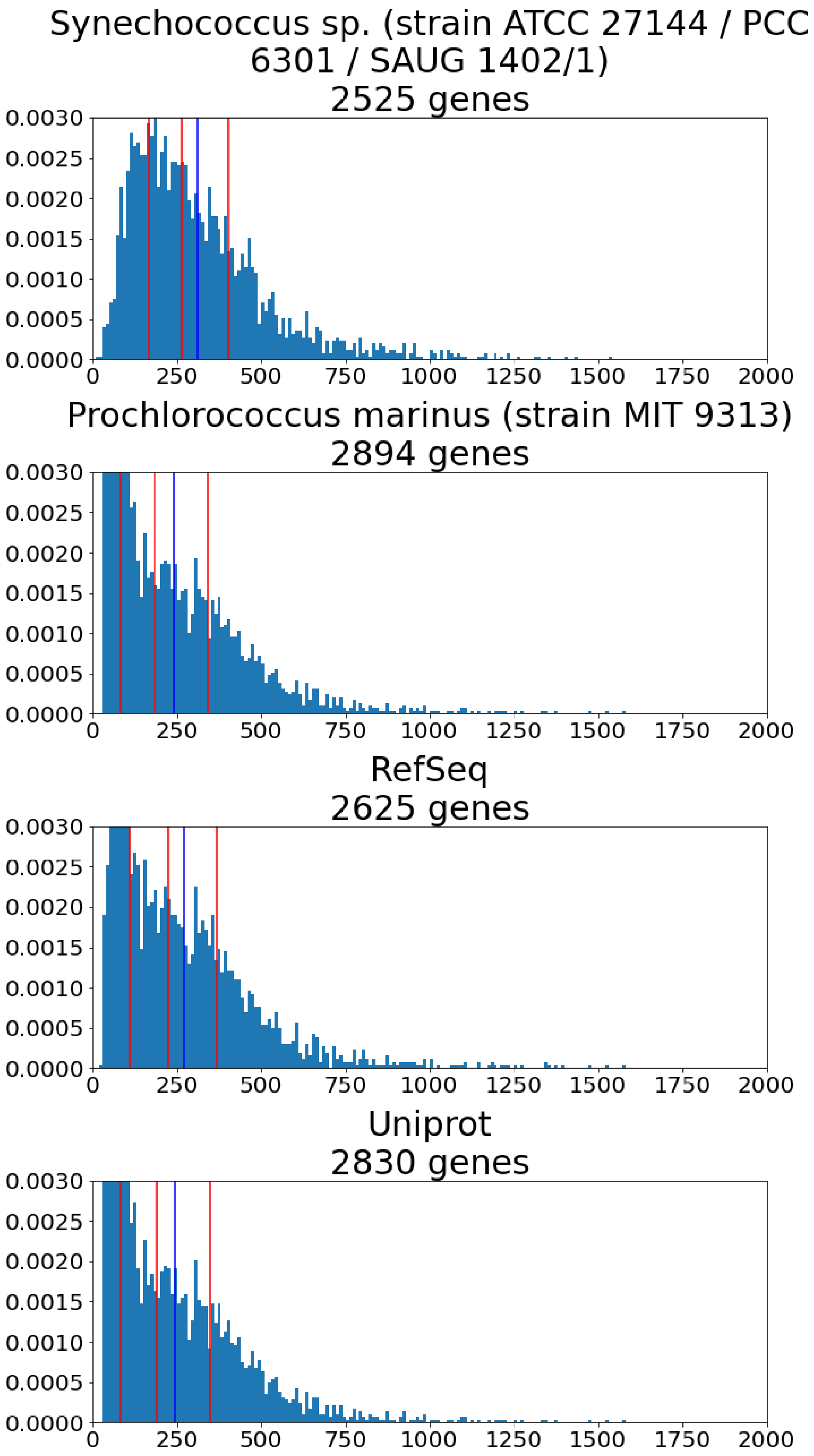
Synechococcus sp. (strain ATCC 27144 / PCC 6301 / SAUG 1402/1)
2525 genes
Prochlorococcus marinus (strain MIT 9313)
2894 genes
RefSeq
2625 genes
Uniprot
2830 genes
0.0000
0.0005
0.0010
0.0015
0.0020
0.0025
0.0030
0
250
500
750
1000
1250
1500
1750
2000

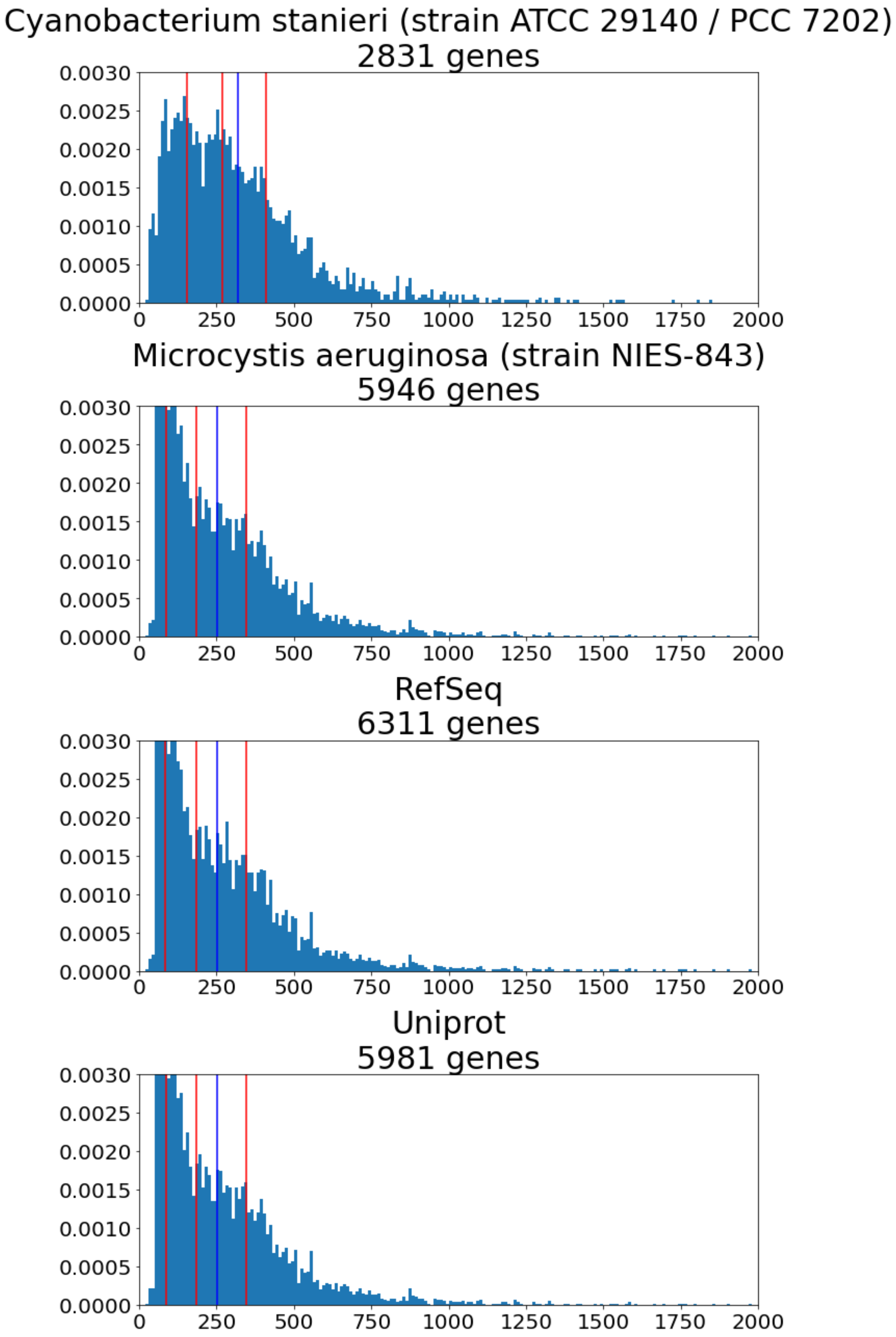
Cyanobacterium stanieri (strain ATCC 29140 / PCC 7202)
2831 genes
Microcystis aeruginosa (strain NIES-843)
5946 genes
RefSeq
6311 genes
Uniprot
5981 genes
0.0030
0.0025
0.0020
0.0015
0.0010
0.0005
0.0000
0
250
500
750
1000
1250
1500
1750
2000

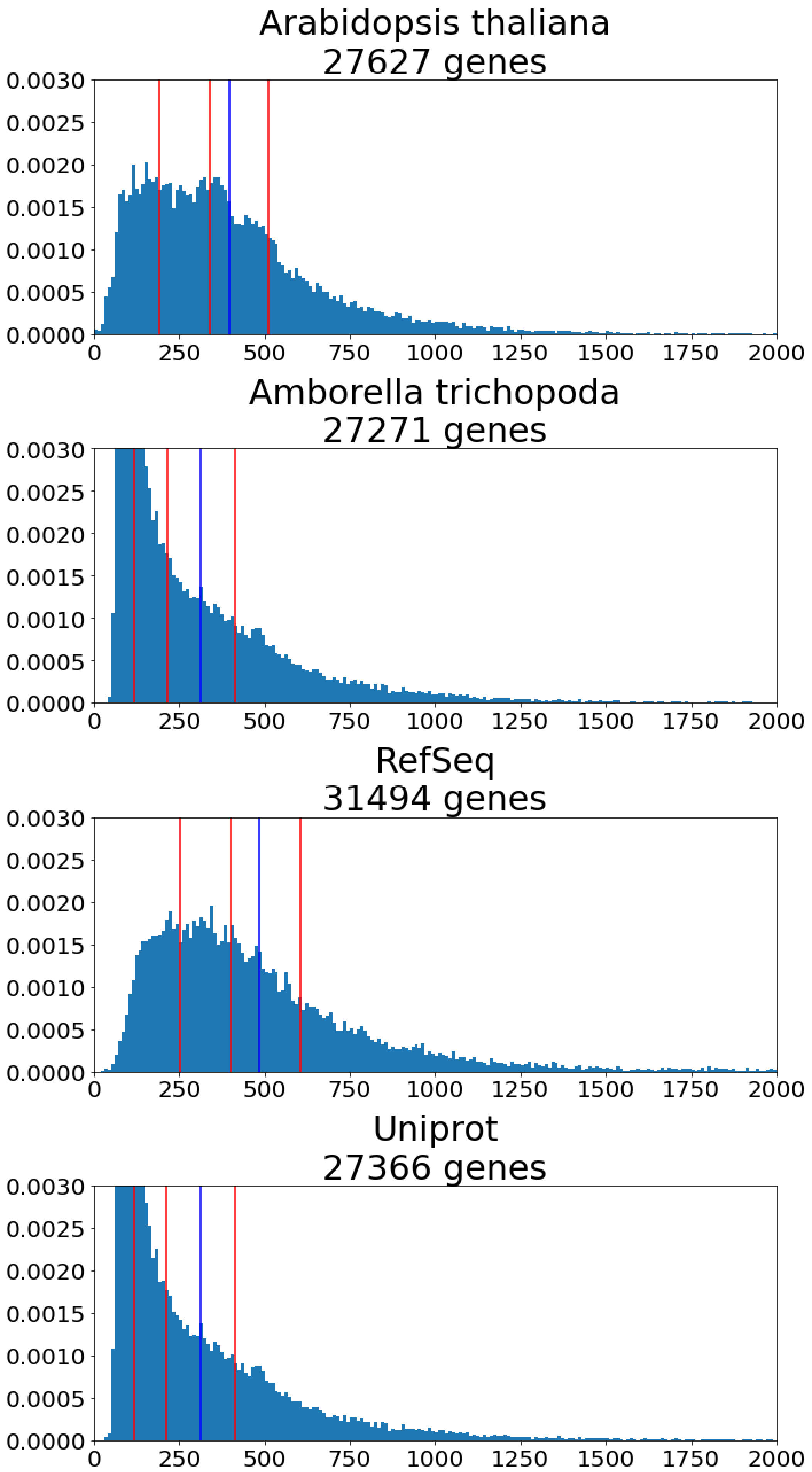
Arabidopsis thaliana
27627 genes
Amborella trichopoda
27271 genes
RefSeq
31494 genes
Uniprot
27366 genes
0.0030
0.0025
0.0020
0.0015
0.0010
0.0005
0.0000
0
250
500
750
1000
1250
1500
1750
2000

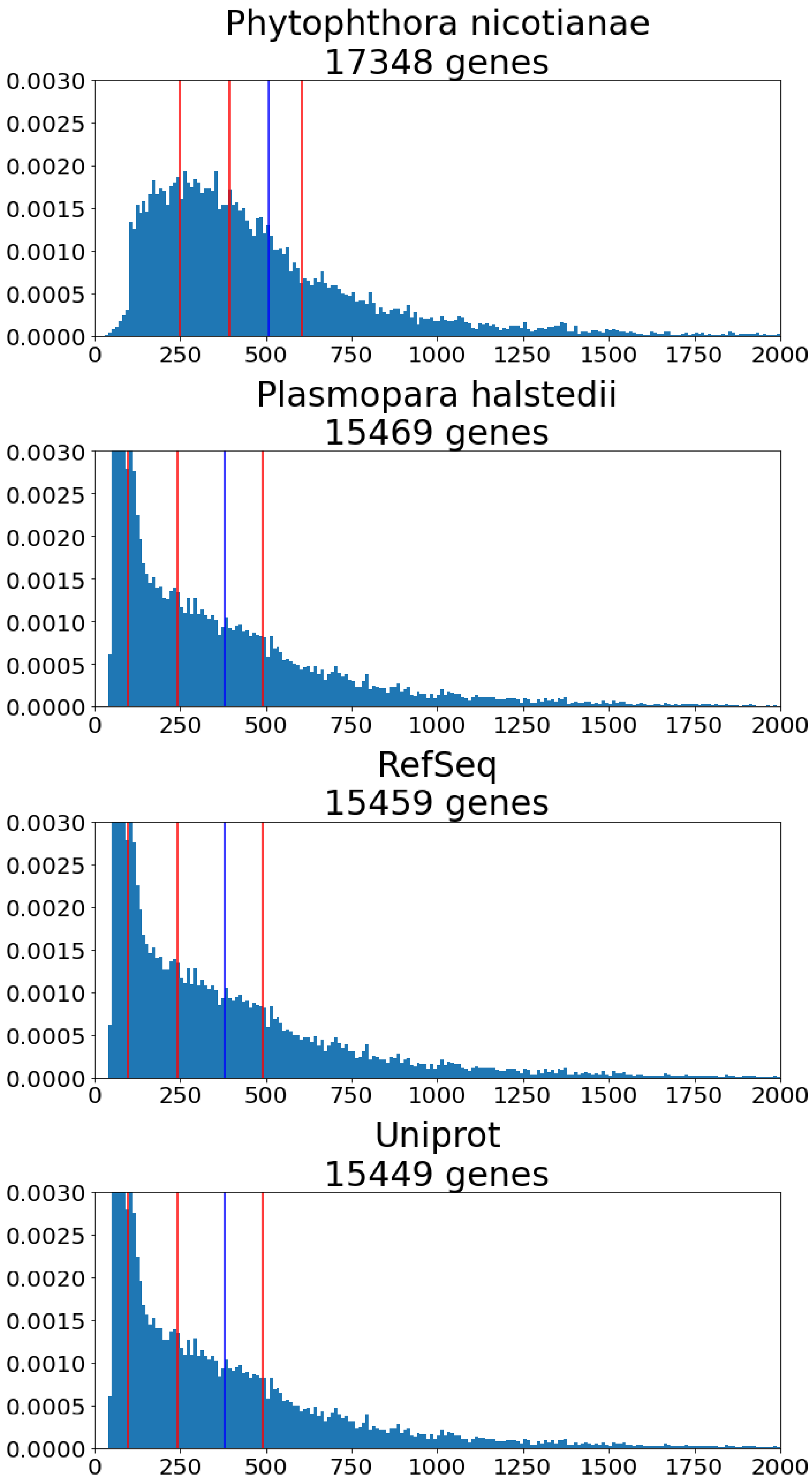
Phytophthora nicotianae
17348 genes
Plasmopara halstedii
15469 genes
RefSeq
15459 genes
Uniprot
15449 genes
0.0000
0.0005
0.0010
0.0015
0.0020
0.0025
0.0030
0
250
500
750
1000
1250
1500
1750
2000

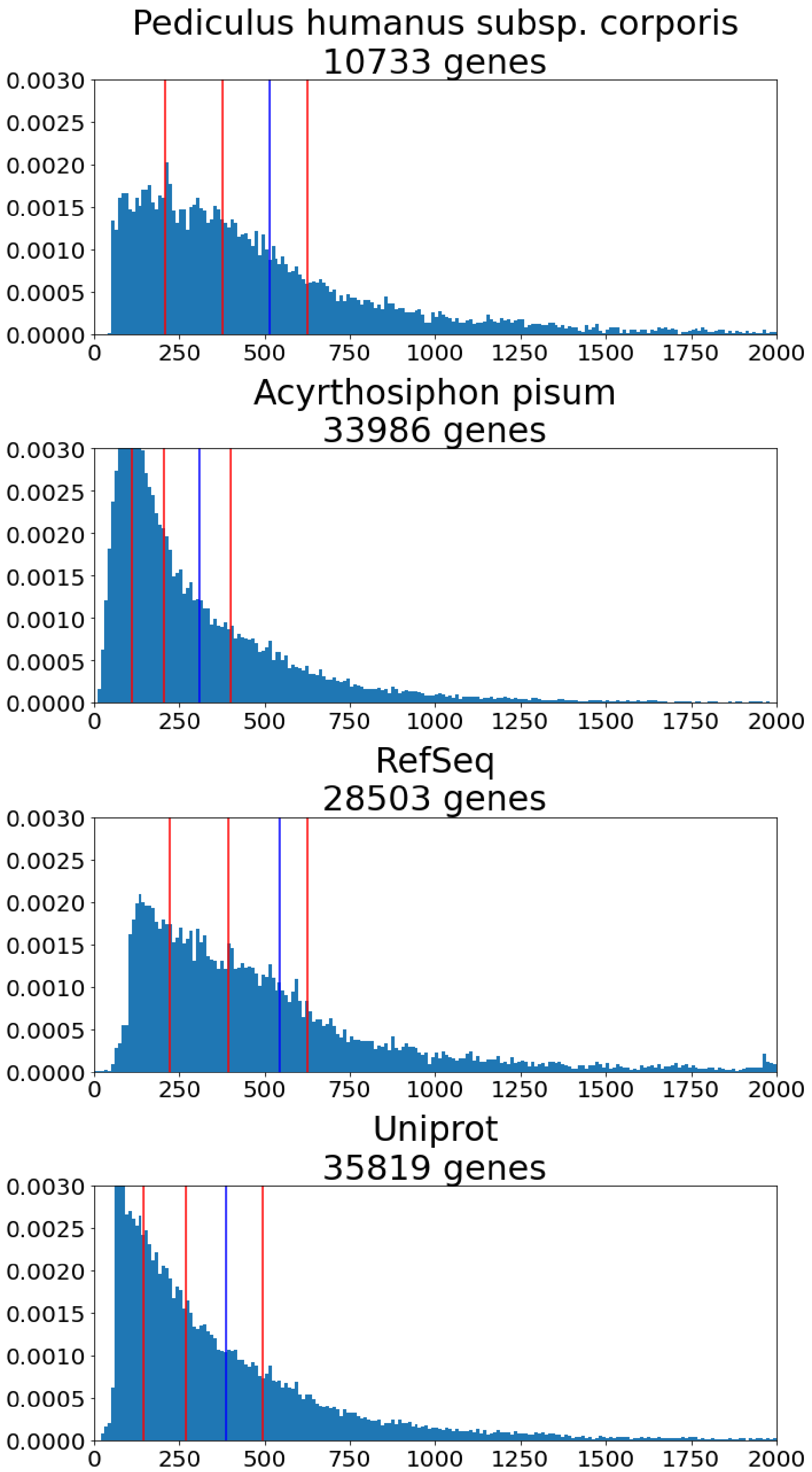
Pediculus humanus subsp. corporis
10733 genes
Acyrthosiphon pisum
33986 genes
RefSeq
28503 genes
Uniprot
35819 genes
0.0000
0.0005
0.0010
0.0015
0.0020
0.0025
0.0030
0
250
500
750
1000
1250
1500
1750
2000

Drosophila melanogaster
13918 genes

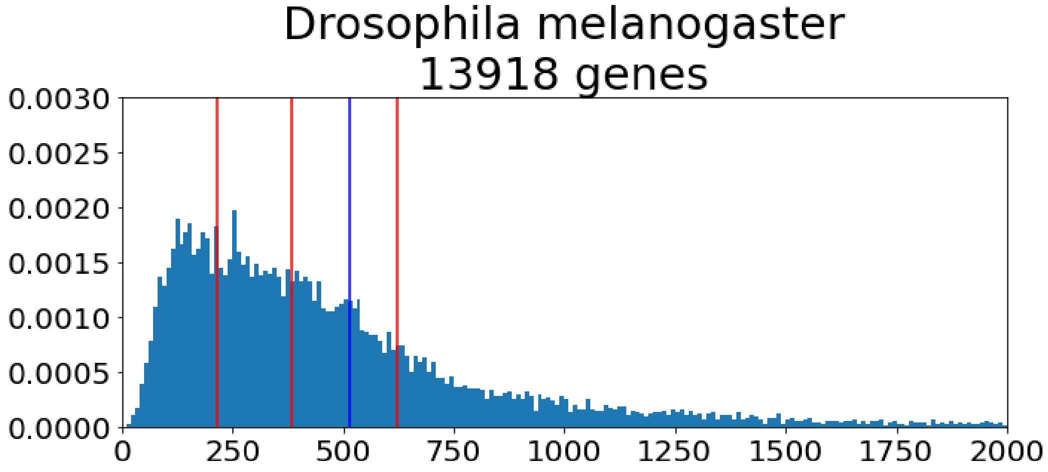

Daphnia pulex
30088 genes

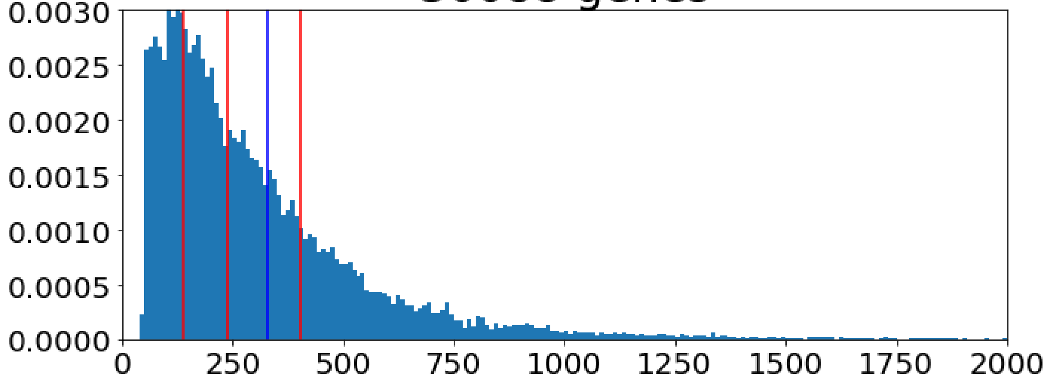

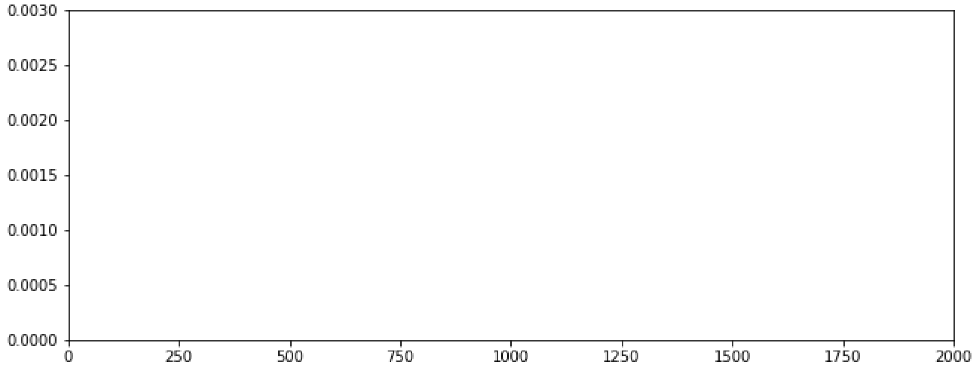

Uniprot
30118 genes

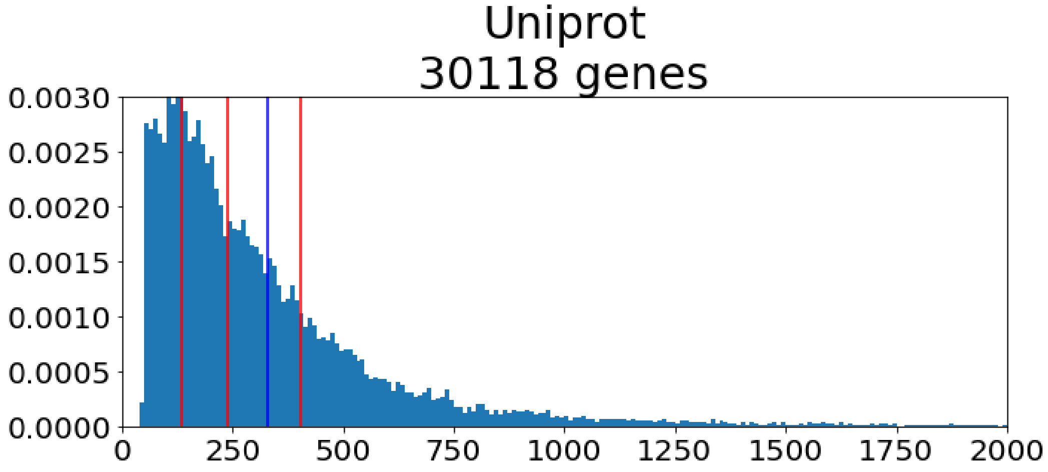

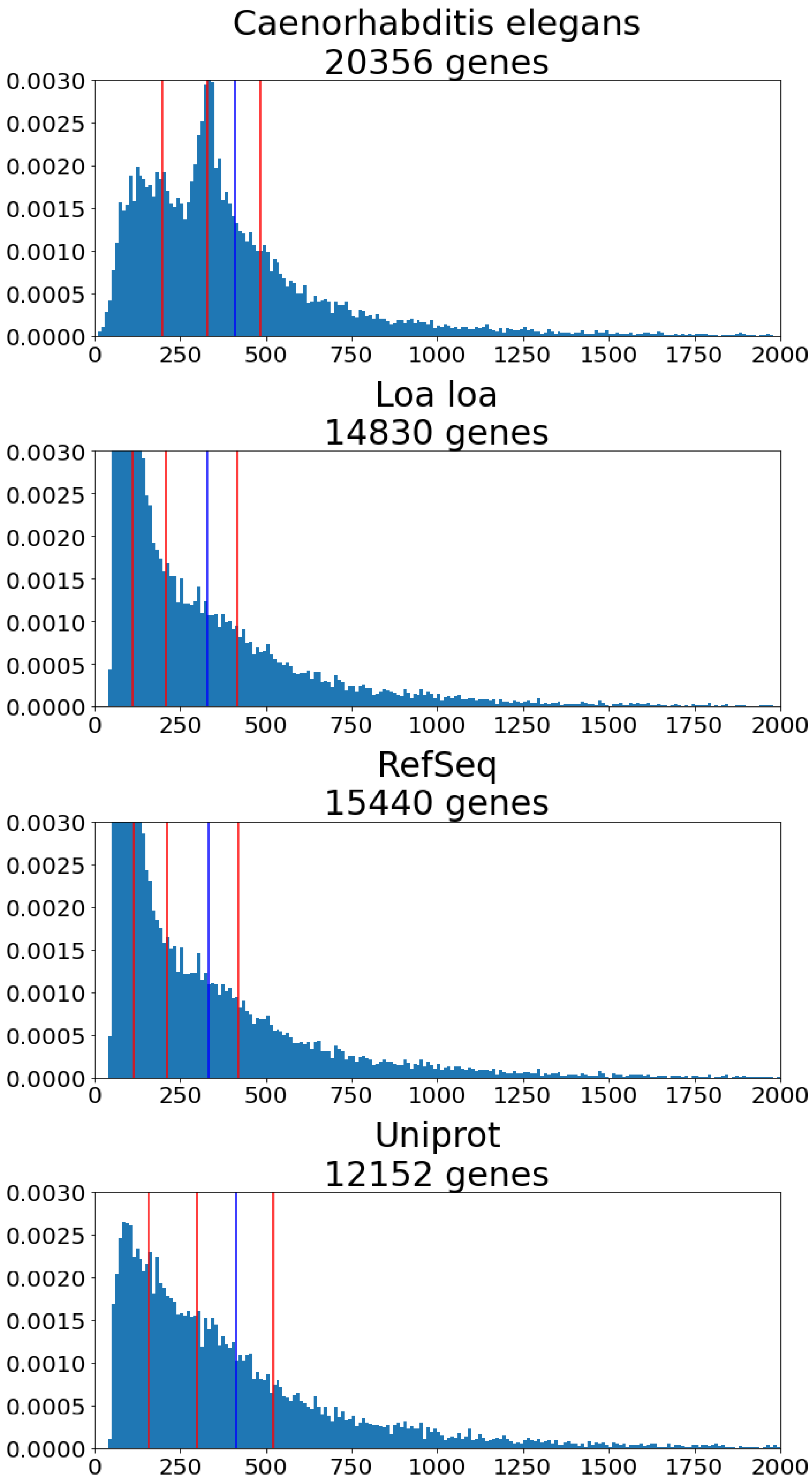
Caenorhabditis elegans
20356 genes
Loa loa
14830 genes
RefSeq
15440 genes
Uniprot
12152 genes
0.0030
0.0025
0.0020
0.0015
0.0010
0.0005
0.0000
0
250
500
750
1000
1250
1500
1750
2000

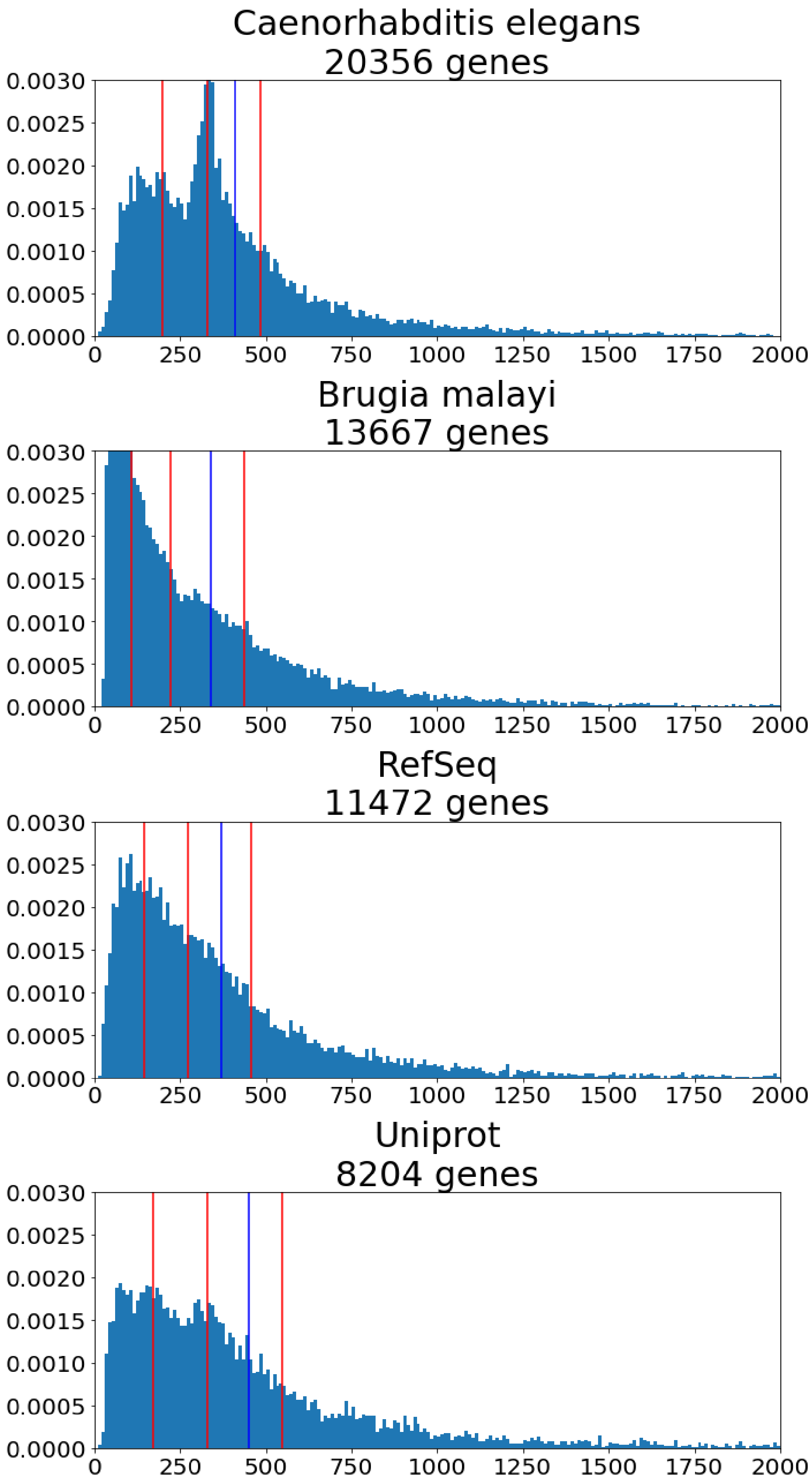
Caenorhabditis elegans
20356 genes
Brugia malayi
13667 genes
RefSeq
11472 genes
Uniprot
8204 genes
0.0030
0.0025
0.0020
0.0015
0.0010
0.0005
0.0000
0
250
500
750
1000
1250
1500
1750
2000

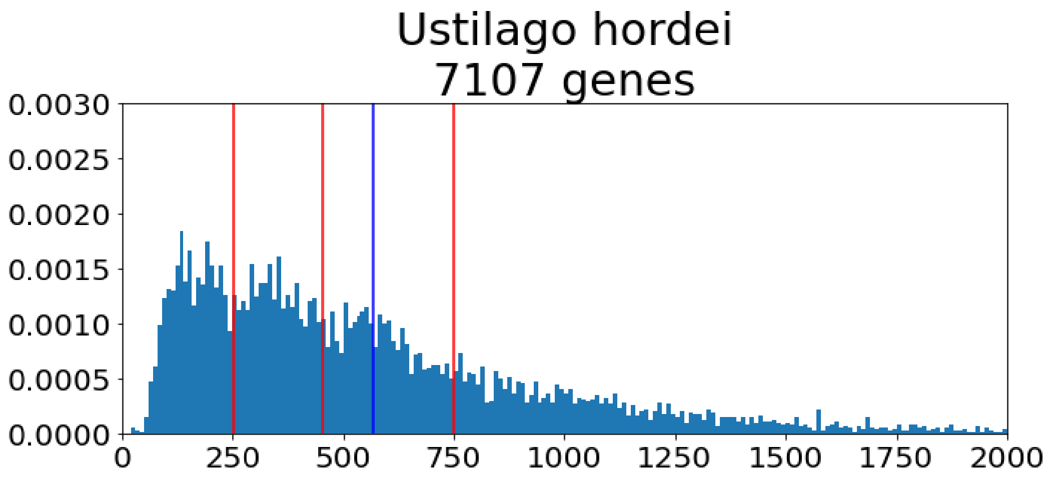
Ustilago hordei
7107 genes
0.0030
0.0025
0.0020
0.0015
0.0010
0.0005
0.0000
0
250
500
750
1000
1250
1500
1750
2000

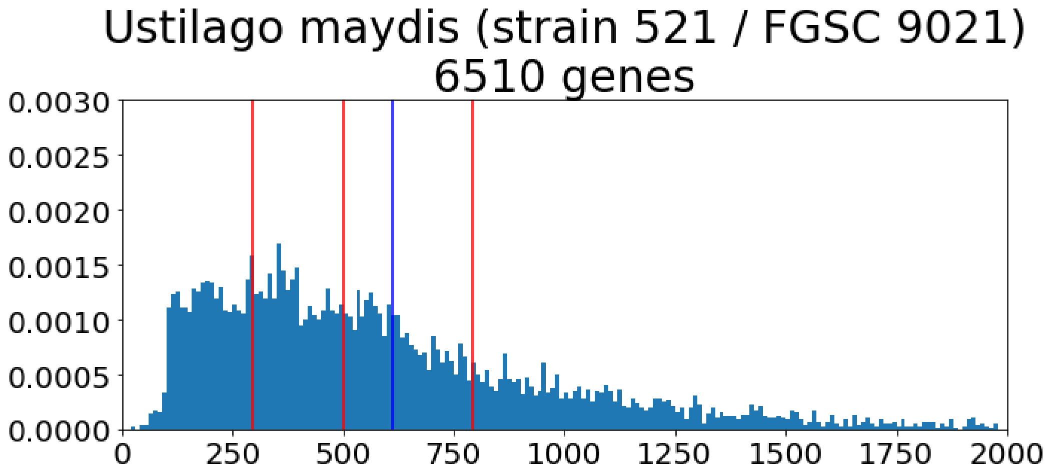
Ustilago maydis (strain 521 / FGSC 9021)
6510 genes
0.0030
0.0025
0.0020
0.0015
0.0010
0.0005
0.0000
0
250
500
750
1000
1250
1500
1750
2000

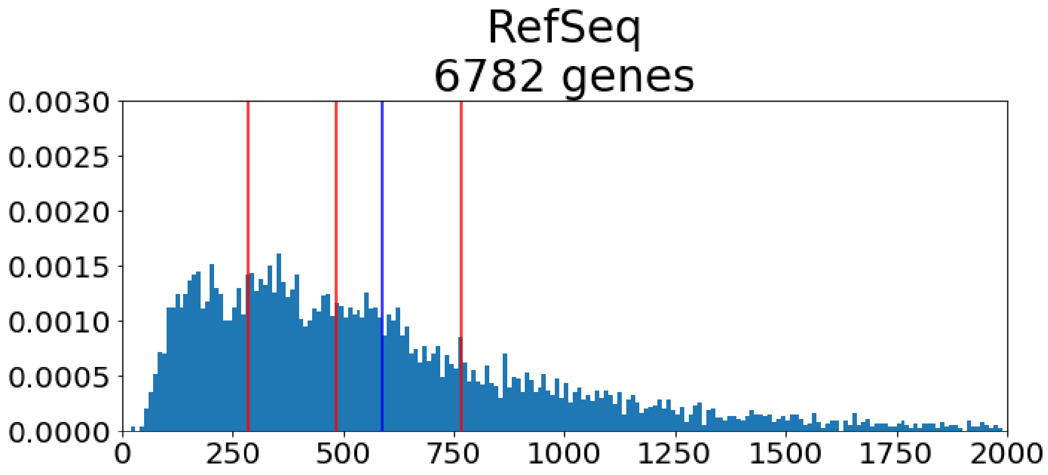
RefSeq
6782 genes
0.0030
0.0025
0.0020
0.0015
0.0010
0.0005
0.0000
0
250
500
750
1000
1250
1500
1750
2000

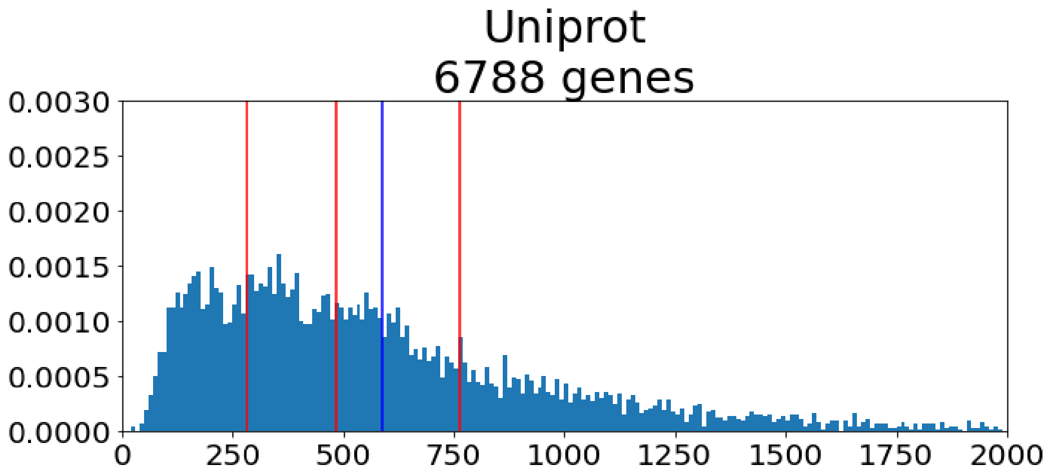
Uniprot
6788 genes
0.0030
0.0025
0.0020
0.0015
0.0010
0.0005
0.0000
0
250
500
750
1000
1250
1500
1750
2000

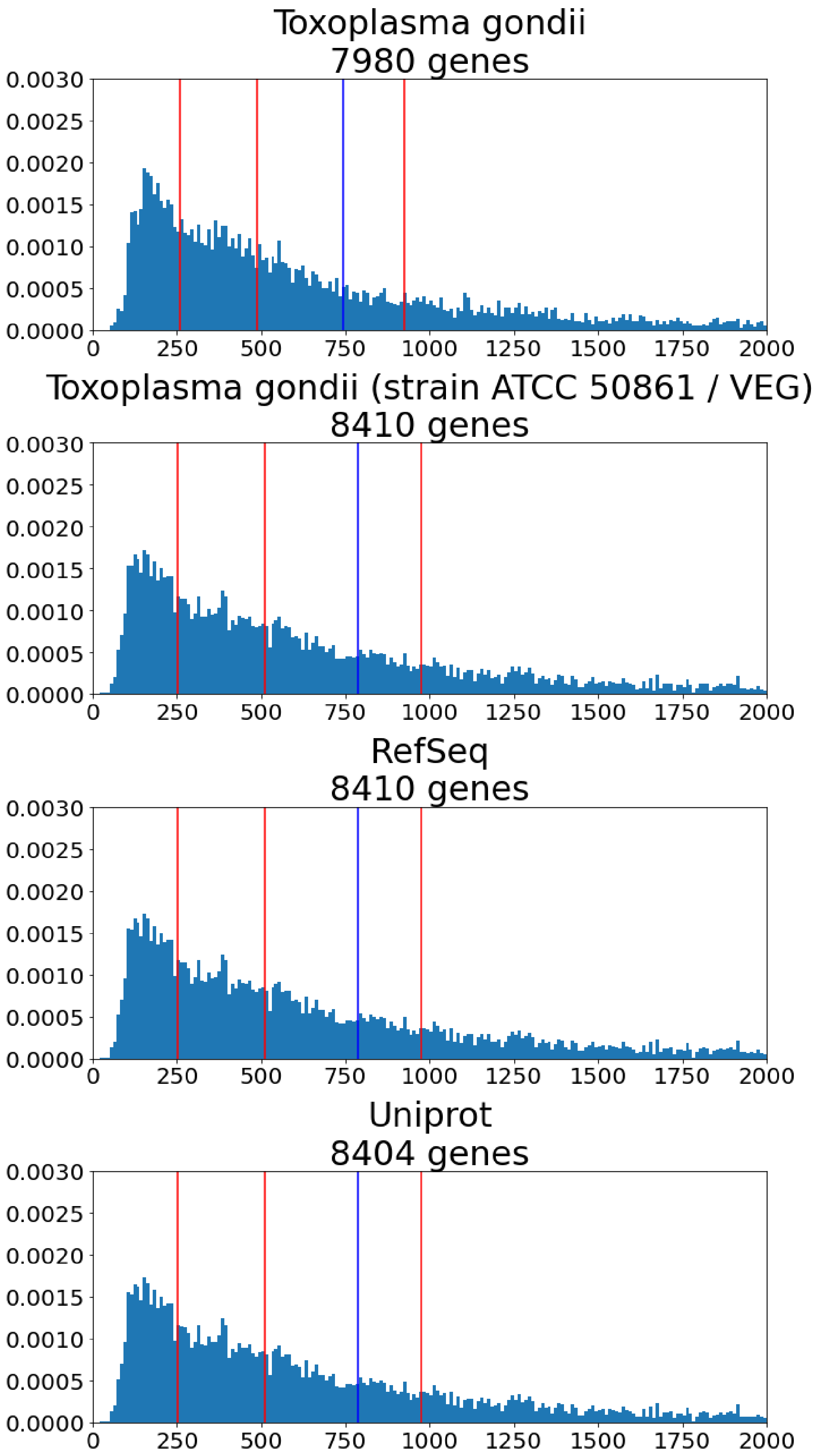
Toxoplasma gondii
7980 genes
Toxoplasma gondii (strain ATCC 50861 / VEG)
8410 genes
RefSeq
8410 genes
Uniprot
8404 genes
0.0000
0.0005
0.0010
0.0015
0.0020
0.0025
0.0030
0
250
500
750
1000
1250
1500
1750
2000

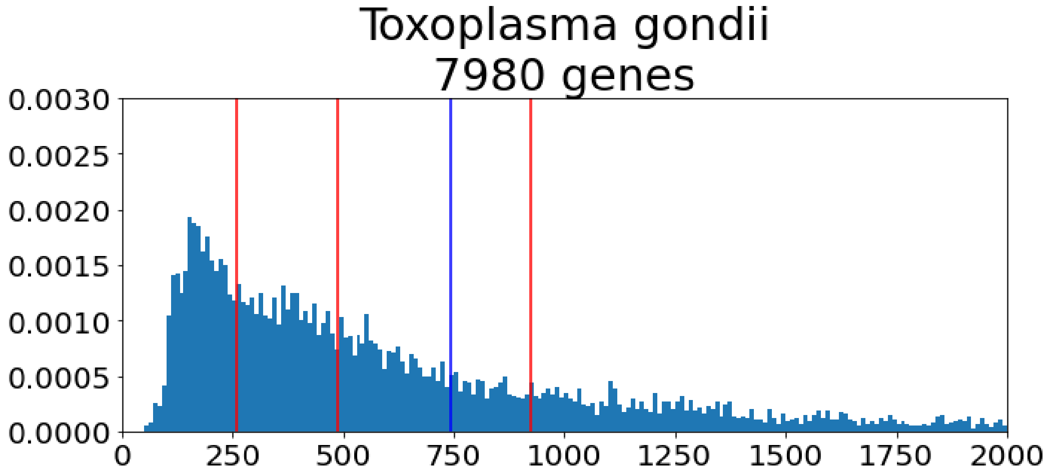
Toxoplasma gondii
7980 genes

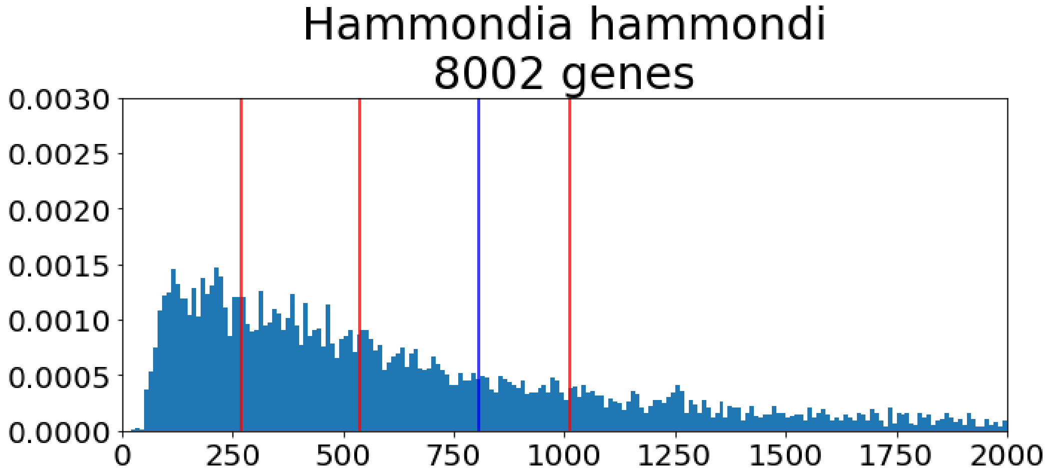
Hammondia hammondi
8002 genes

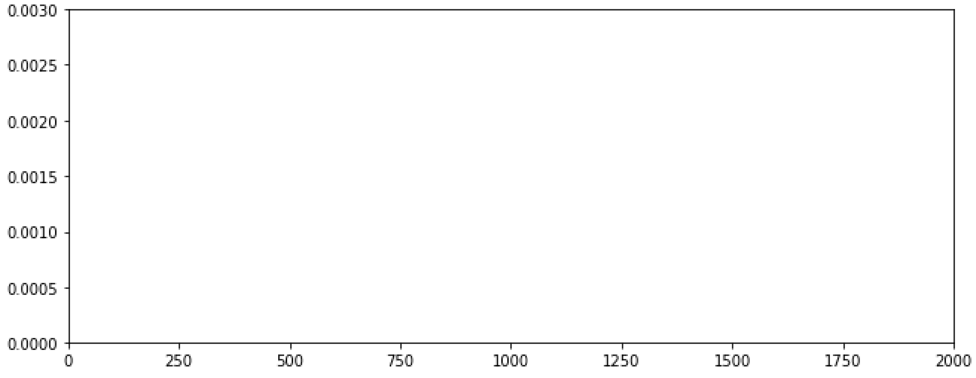

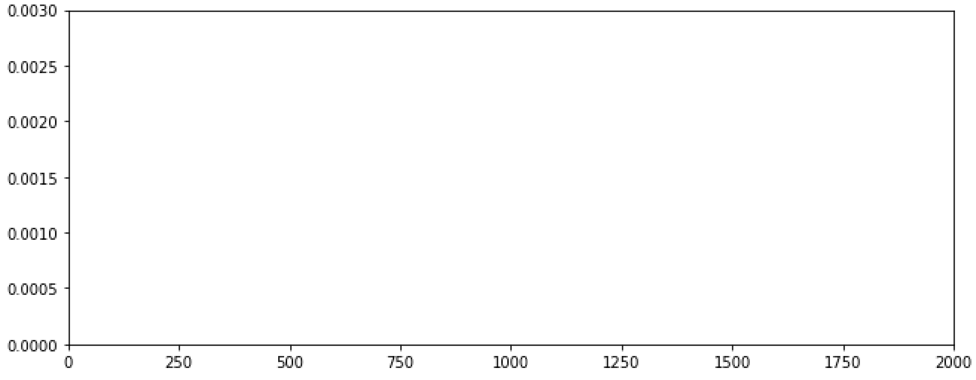

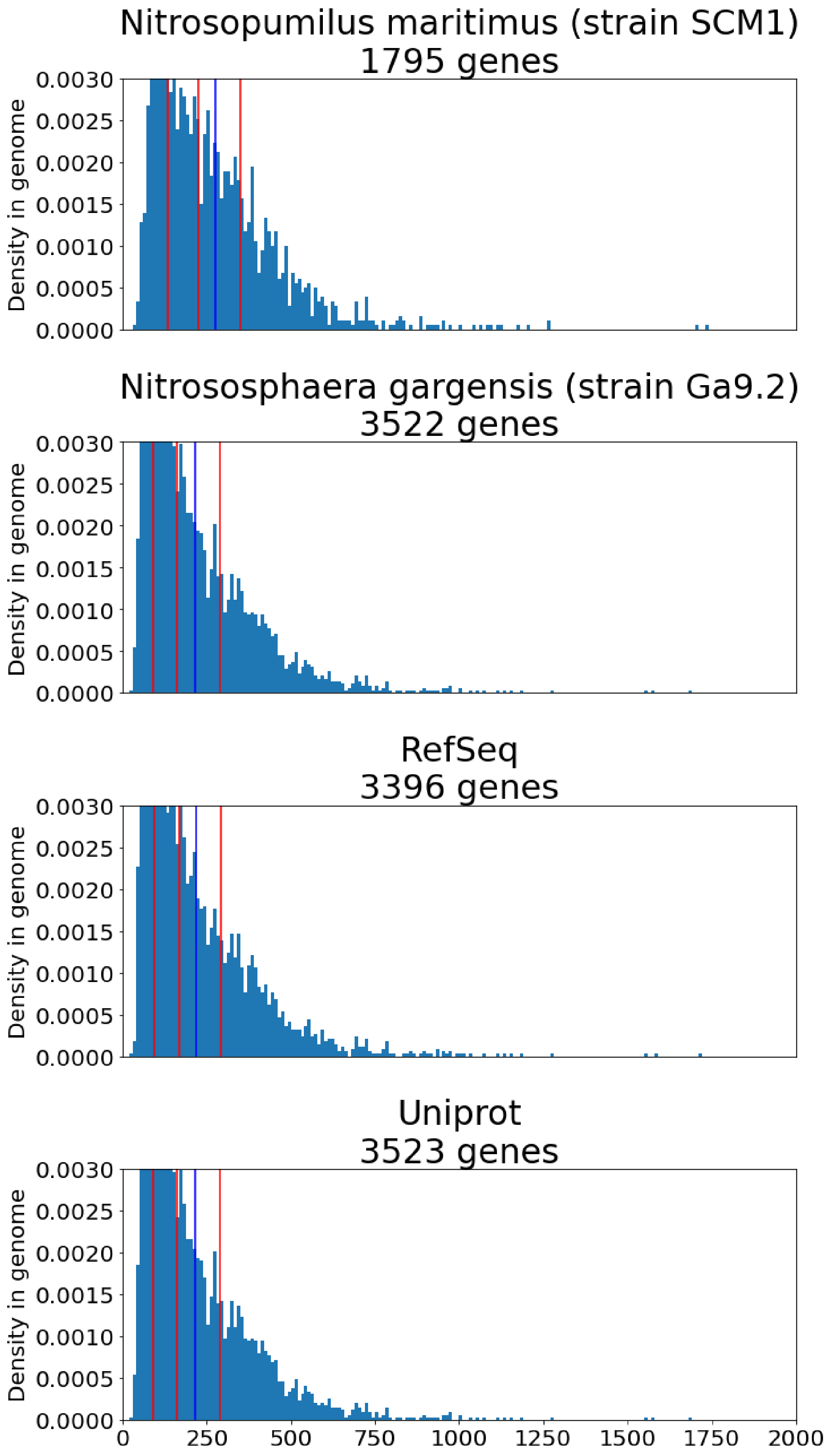

Nitrosopumilus maritimus (strain SCM1)
1795 genes
Nitrososphaera gargensis (strain Ga9.2)
3522 genes
RefSeq
3396 genes
Uniprot
3523 genes
Density in genome
0.0000
0.0005
0.0010
0.0015
0.0020
0.0025
0.0030
0
250
500
750
1000
1250
1500
1750
2000

Figure S37: Length distribution in the Rickettsia genus. Left column represents length distribution in OMA (this study's dataset), the center column, the length distribution in RefSeq, and the right column the length distribution in Uniprot. The two rightmost columns are only filled for proteomes for which the distribution in OMA was labeled as outlier.

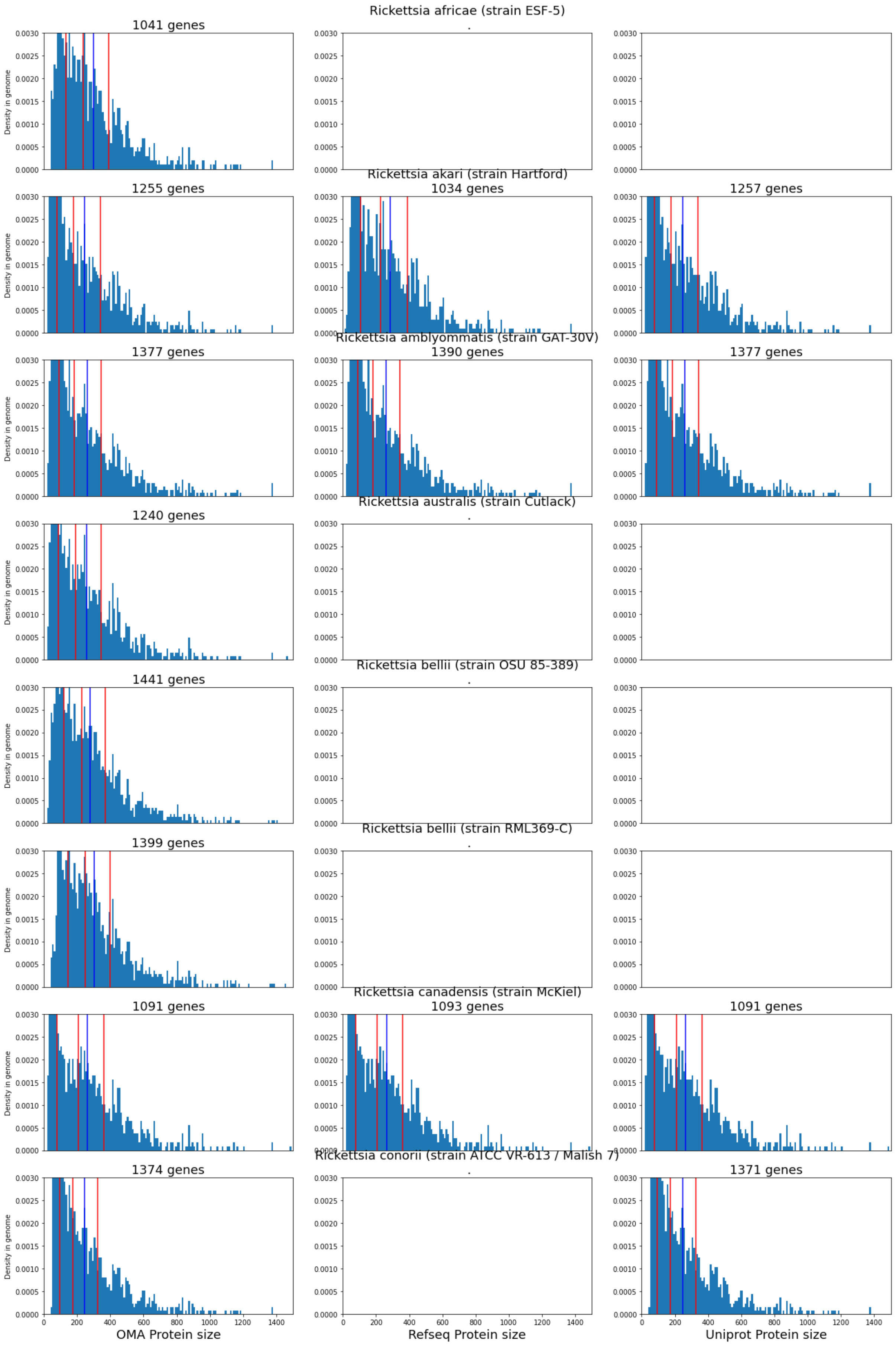
Rickettsia africae (strain ESF-5)
1041 genes
Rickettsia akari (strain Hartford)
1255 genes
1034 genes
1257 genes
Rickettsia amblyommatis (strain GAT-30V)
1377 genes
1390 genes
1377 genes
Rickettsia australis (strain Cutlack)
1240 genes
Rickettsia bellii (strain OSU 85-389)
1441 genes
Rickettsia bellii (strain RML369-C)
1399 genes
Rickettsia canadensis (strain McKiel)
1091 genes
1093 genes
1091 genes
Rickettsia conorii (strain ATCC VR-613 / Malish 7)
1374 genes
1371 genes
Density in genome
OMA Protein size
Refseq Protein size
Uniprot Protein size

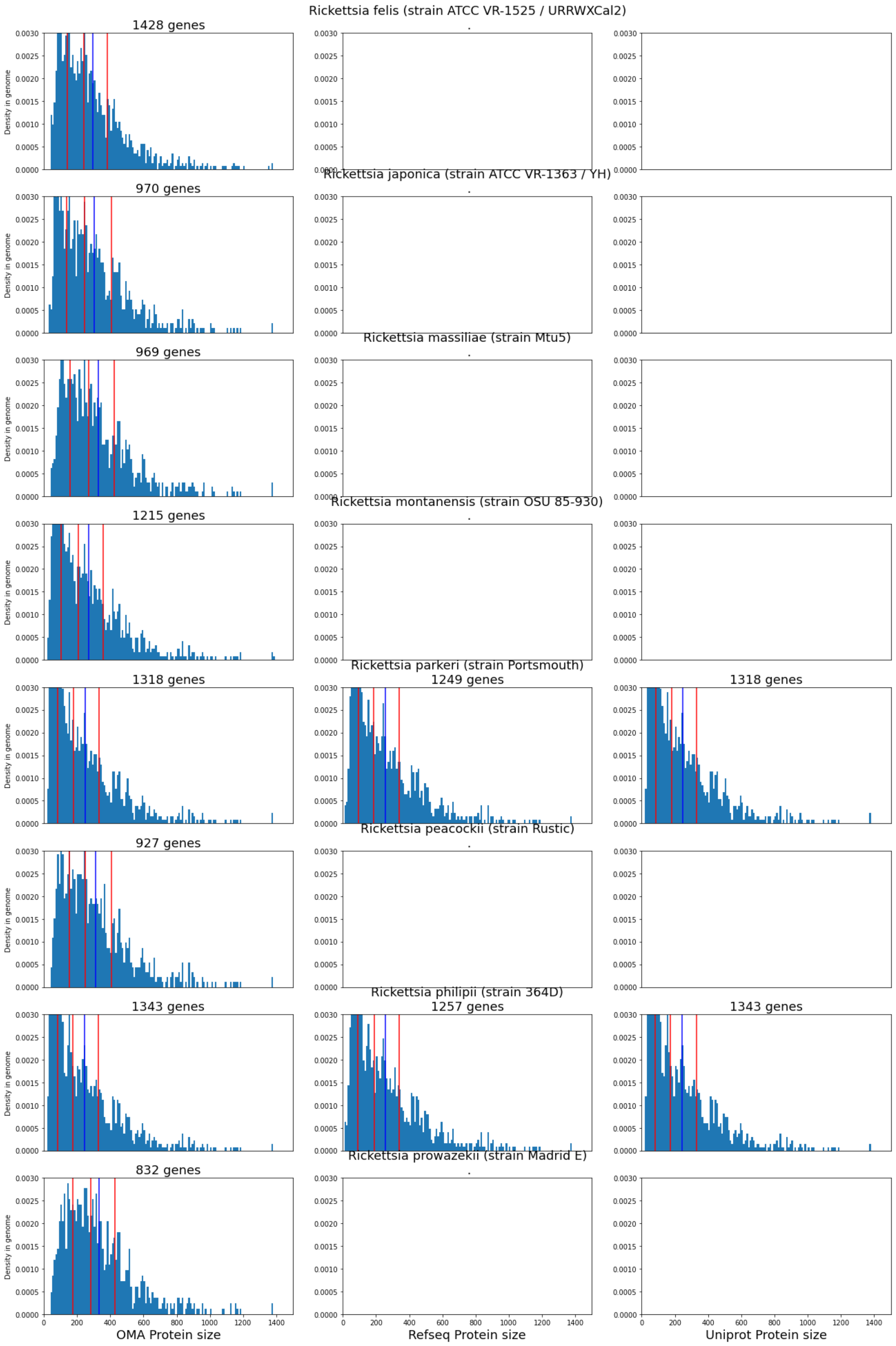
Rickettsia felis (strain ATCC VR-1525 / URRWXCal2)
1428 genes
Rickettsia japonica (strain ATCC VR-1363 / YH)
970 genes
Rickettsia massiliae (strain Mtu5)
969 genes
Rickettsia montanensis (strain OSU 85-930)
1215 genes
Rickettsia parkeri (strain Portsmouth)
1318 genes
1249 genes
1318 genes
Rickettsia peacockii (strain Rustic)
927 genes
Rickettsia philipii (strain 364D)
1343 genes
1257 genes
1343 genes
Rickettsia prowazekii (strain Madrid E)
832 genes
Density in genome
OMA Protein size
Refseq Protein size
Uniprot Protein size

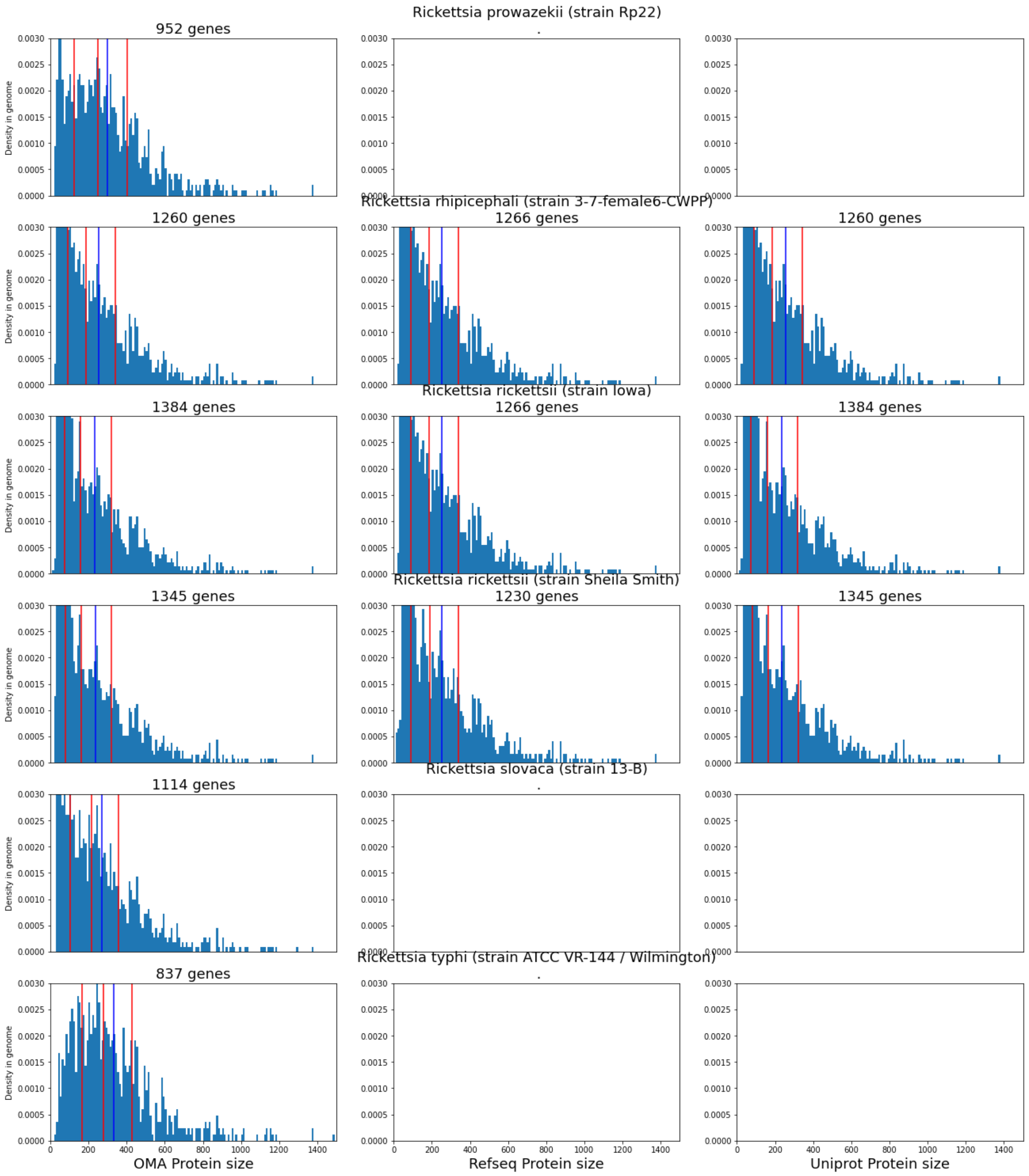
Rickettsia prowazekii (strain Rp22)
952 genes
Rickettsia rhipicephali (strain 3-7-female6-CWPP)
1260 genes
1266 genes
1260 genes
Rickettsia rickettsii (strain Iowa)
1384 genes
1266 genes
1384 genes
Rickettsia rickettsii (strain Sheila Smith)
1345 genes
1230 genes
1345 genes
Rickettsia slovaca (strain 13-B)
1114 genes
Rickettsia typhi (strain ATCC VR-144 / Wilmington)
837 genes
Density in genome
OMA Protein size
Refseq Protein size
Uniprot Protein size

Figure S36 : Protein length distributions in Apicomplexa proteomes. Each plot represents the protein length distribution of the species proteome in OMA. Only length up to 2000 aa are represented. Species name is indicated as the top, with the number of proteins in the proteome. Red lines indicate, from left to right: 1st quartile, median and 3rd quartile of protein length. The blue line represents the mean.

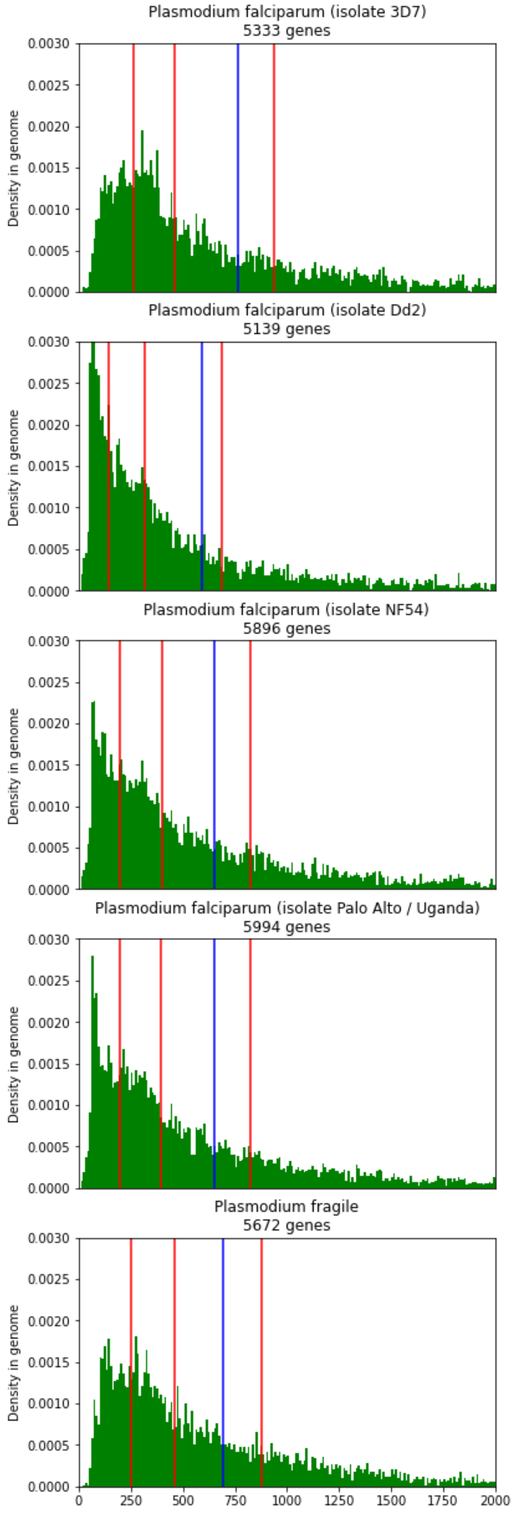

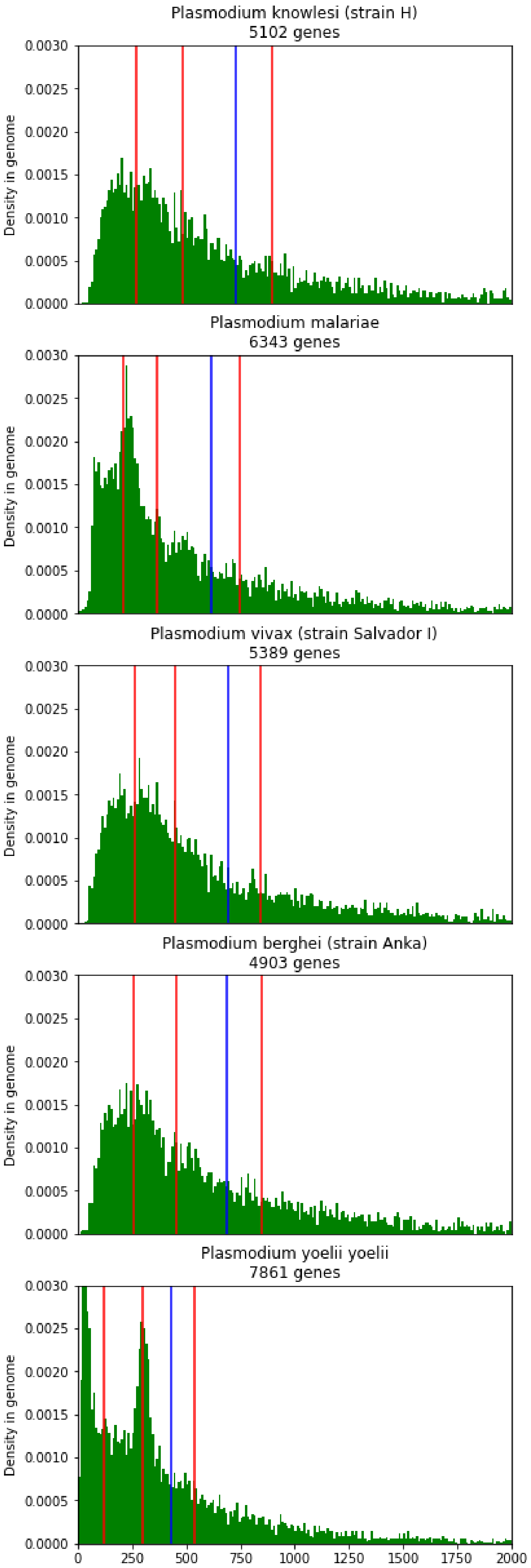

Plasmodium knowlesi (strain H)
5102 genes
Plasmodium malariae
6343 genes
Plasmodium vivax (strain Salvador I)
5389 genes
Plasmodium berghei (strain Anka)
4903 genes
Plasmodium yoelii yoelii
7861 genes
Density in genome
0.0000
0.0005
0.0010
0.0015
0.0020
0.0025
0.0030
0
250
500
750
1000
1250
1500
1750
2000

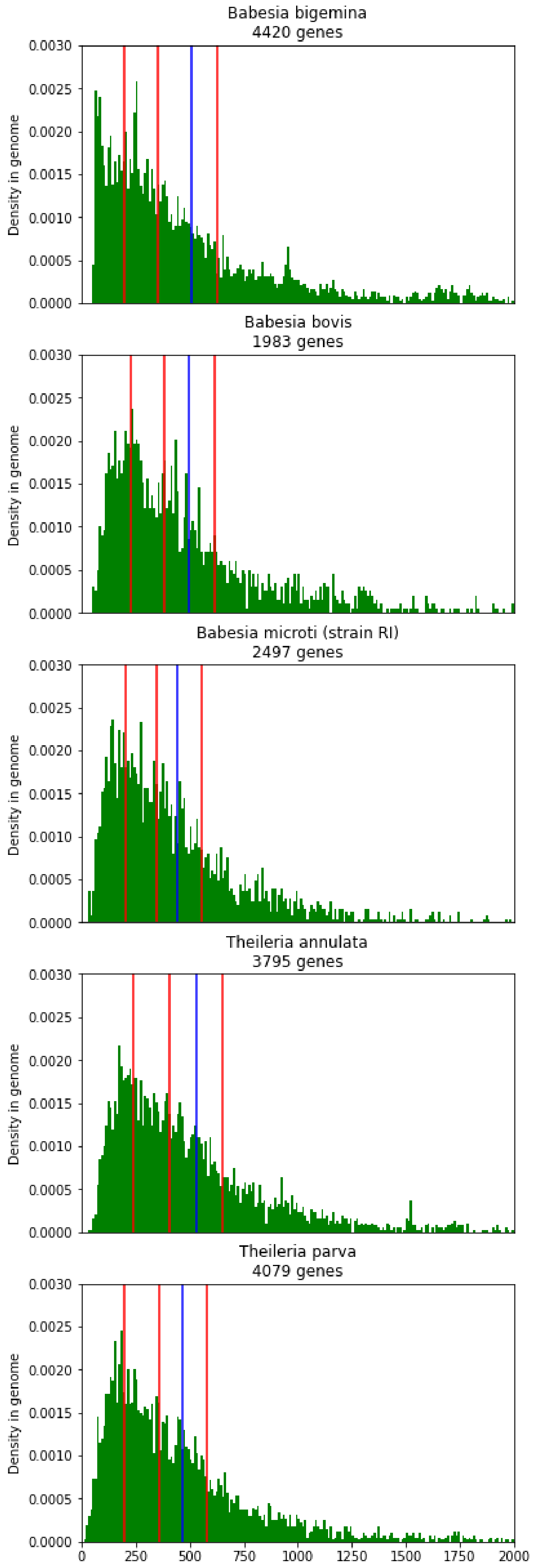
Babesia bigemina
4420 genes
Babesia bovis
1983 genes
Babesia microti (strain RI)
2497 genes
Theileria annulata
3795 genes
Theileria parva
4079 genes
Density in genome
0.0000
0.0005
0.0010
0.0015
0.0020
0.0025
0.0030
0
250
500
750
1000
1250
1500
1750
2000

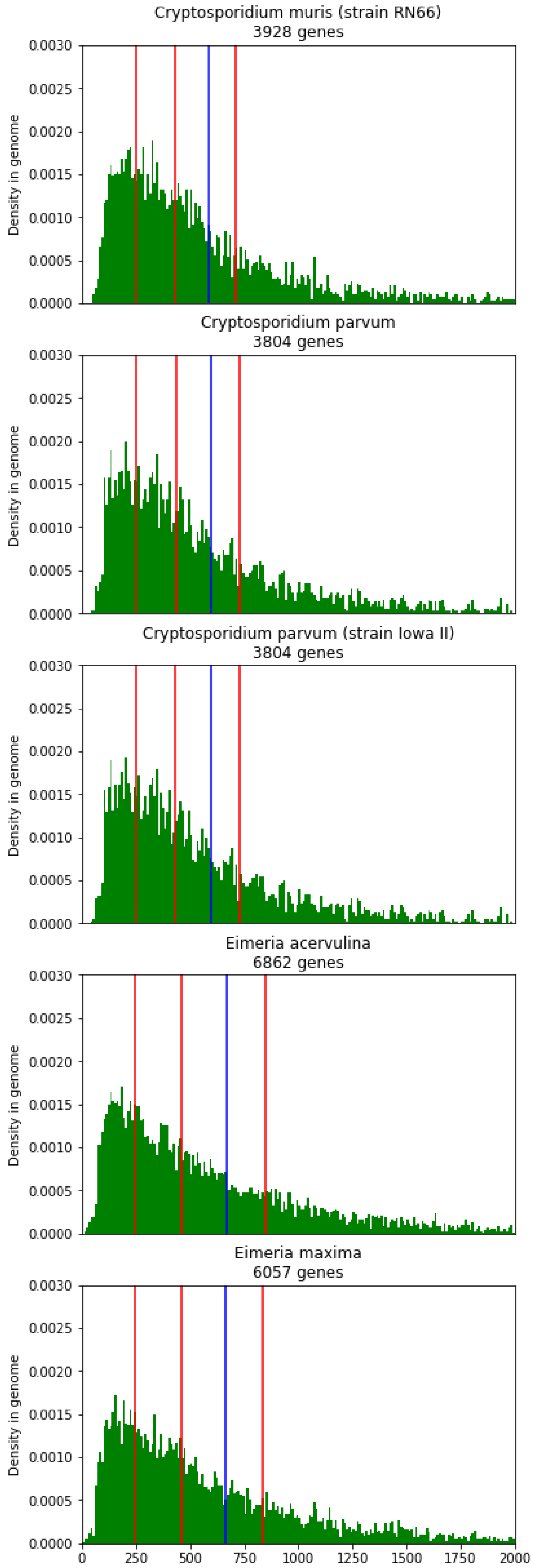
Cryptosporidium muris (strain RN66)
3928 genes
Cryptosporidium parvum
3804 genes
Cryptosporidium parvum (strain Iowa II)
3804 genes
Eimeria acervulina
6862 genes
Eimeria maxima
6057 genes
Density in genome
0.0030
0.0025
0.0020
0.0015
0.0010
0.0005
0.0000
0
250
500
750
1000
1250
1500
1750
2000

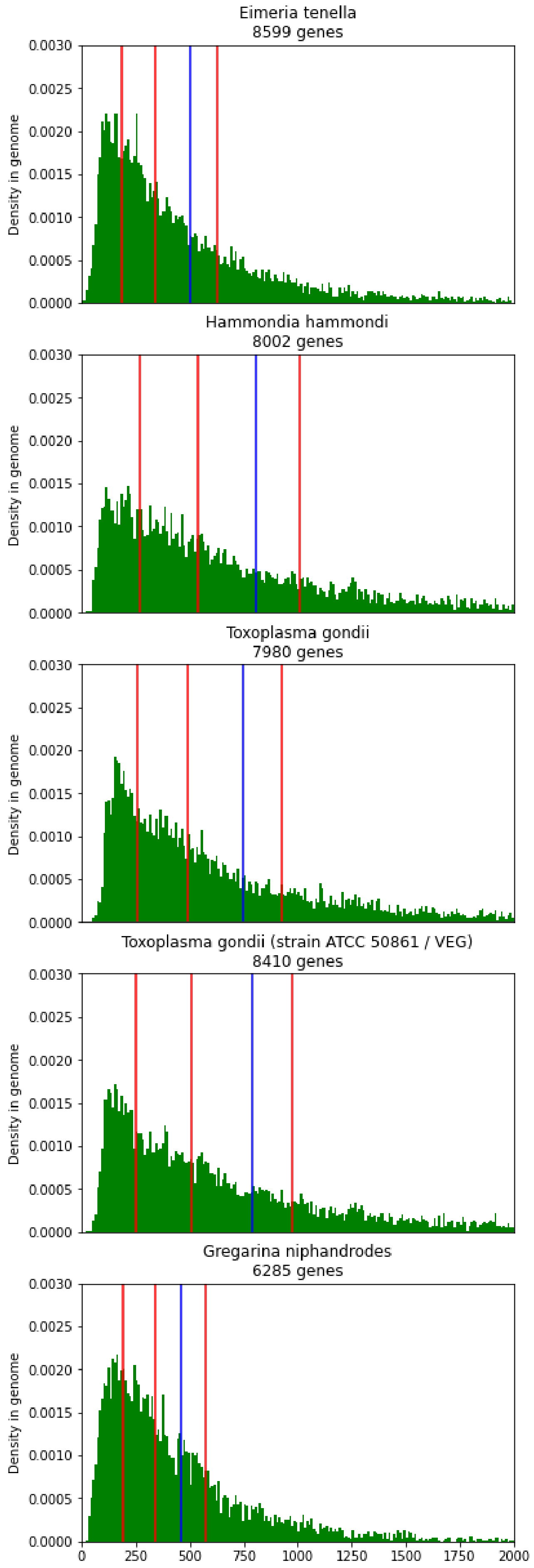
Eimeria tenella
8599 genes
Hammondia hammondi
8002 genes
Toxoplasma gondii
7980 genes
Toxoplasma gondii (strain ATCC 50861 / VEG)
8410 genes
Gregarina niphandrodes
6285 genes
Density in genome
0.0000
0.0005
0.0010
0.0015
0.0020
0.0025
0.0030
0
250
500
750
1000
1250
1500
1750
2000

Figure S37: Protein length distributions in proteomes from the *Ustilago* genus. Each plot represents the protein length distribution of the species proteome in OMA. Only length up to 2000 aa are represented. Species name is indicated as the top, with the number of proteins in the proteome. Red lines indicate, from left to right : 1st quartile, median and 3rd quartile of protein length. The blue line represents the mean.

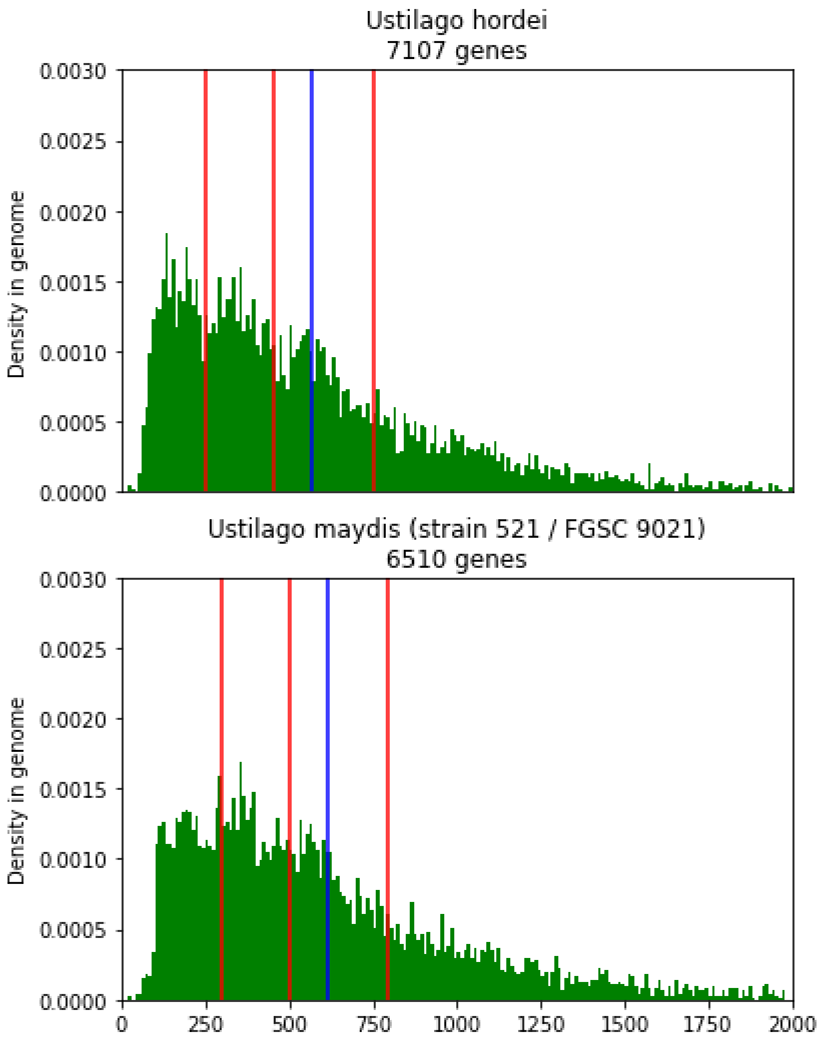

Figure S38 : Gene ontology enrichment of long genes for *Ustilago maydis*. Enrichment of genes larger than 1,000 aa for the different GO categories (top: BP- biological process, middle: MF - Molecular Function, bottom : CC - Cellular component), with the background set being either the whole gene repertoire of the species (left) or all the proteins in the dataset larger than 1,000 aa (right). Results are shown in semantic similarity scatterplots, which summarize the enriched GO terms by removing term redundancy. The p-values for the enriched terms are shown by color, and the number of terms each circle represents is shown by its size.

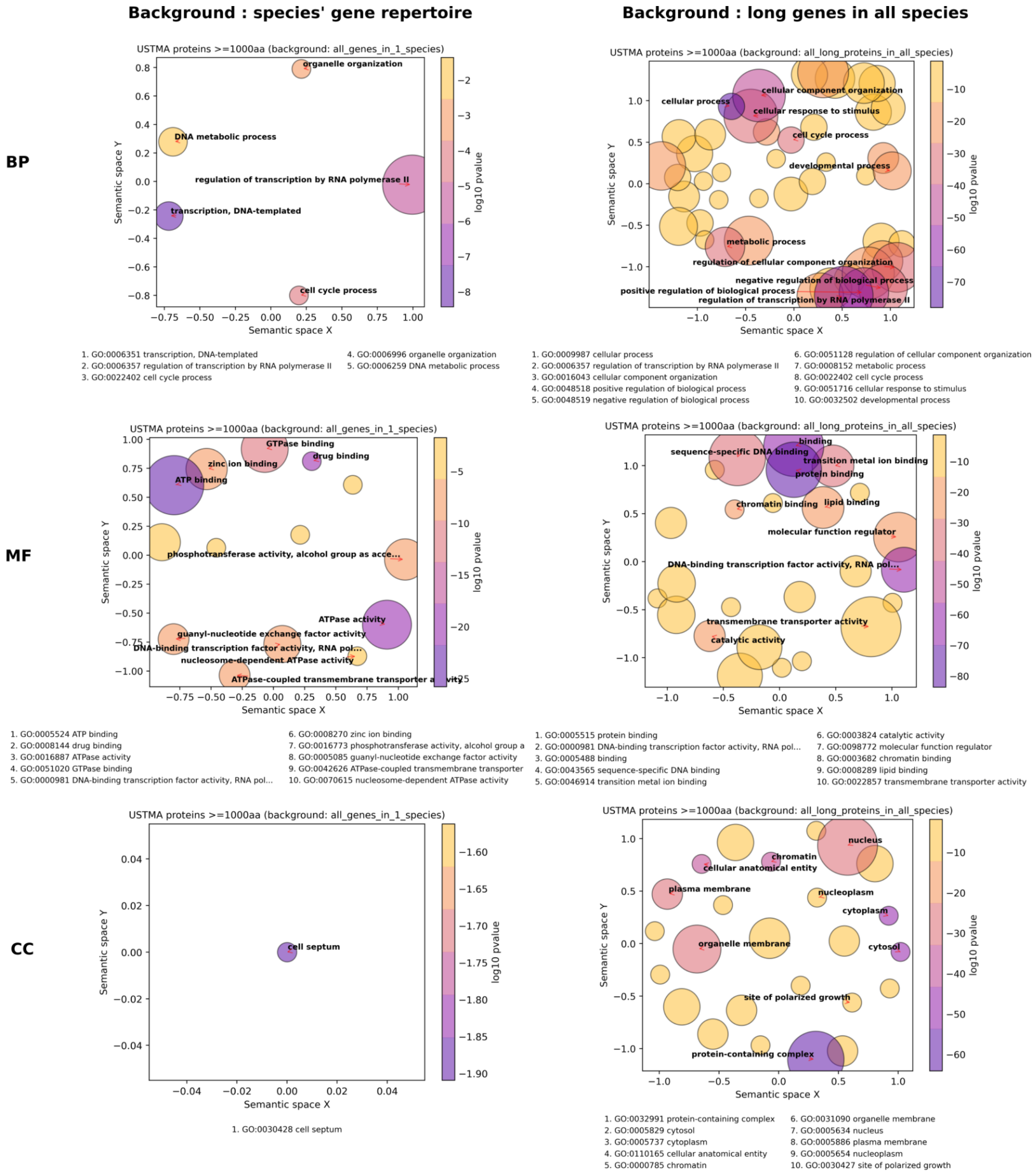

Figure S39: Gene ontology enrichment of long genes for *Toxoplasma gondii* (strain VEG). Enrichment of genes larger than 1,000 aa for the different GO categories (top: BP- biological process, middle: MF - Molecular Function, bottom : CC - Cellular component), with the background set being either the whole gene repertoire of the species (left) or all the proteins in the dataset larger than 1,000 aa (right). Results are shown in semantic similarity scatterplots, which summarize the enriched GO terms by removing term redundancy. The p-values for the enriched terms are shown by color, and the number of terms each circle represents is shown by its size.

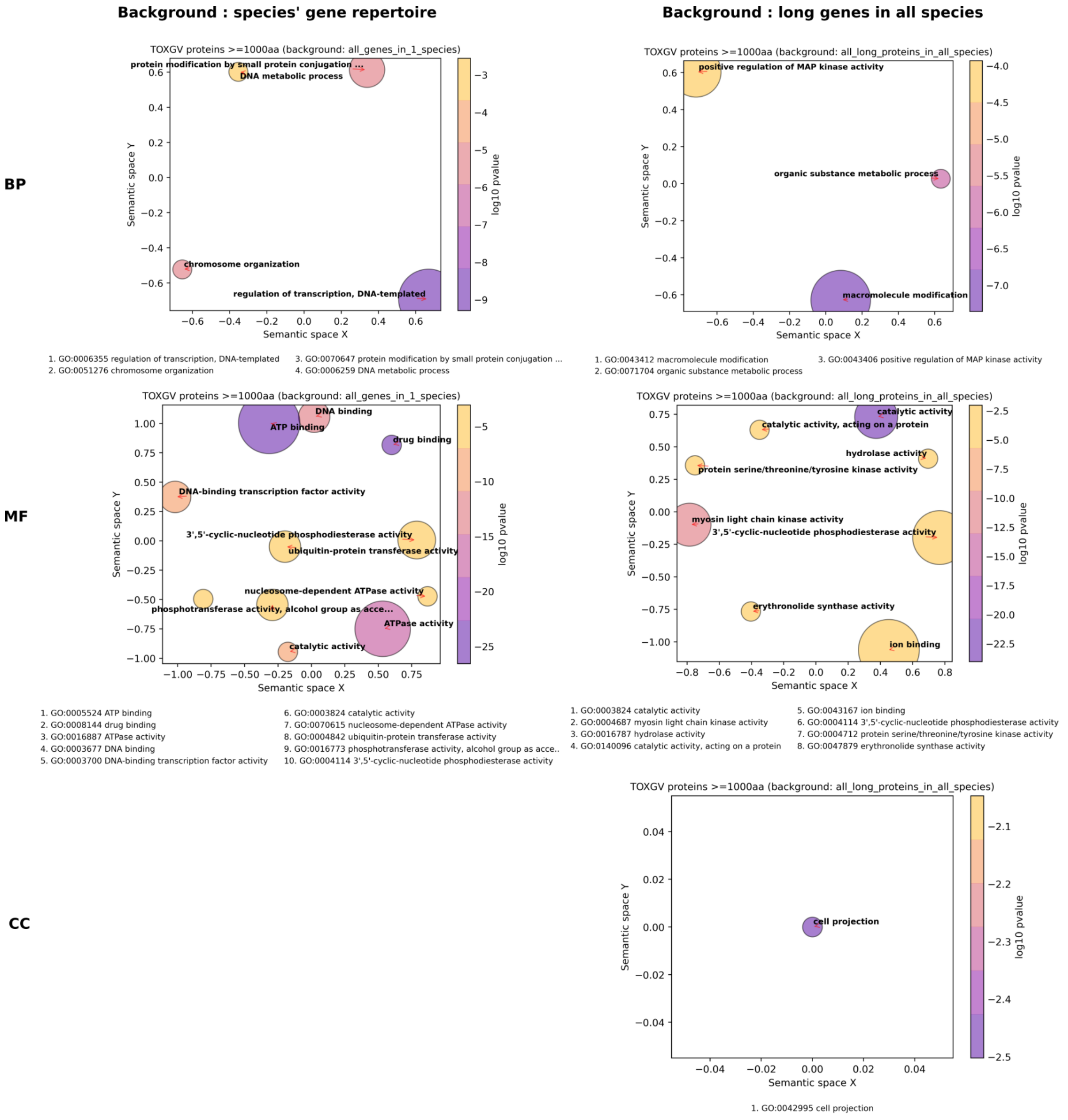

Figure S40: Gene ontology enrichment of long genes for *Plasmodium falciparum* (isolate 3D7**).** Enrichment of genes larger than 1,000 aa for the different GO categories (top: BP- biological process, middle: MF - Molecular Function, bottom : CC - Cellular component), with the background set being either the whole gene repertoire of the species (left) or all the proteins in the dataset larger than 1,000 aa (right). Results are shown in semantic similarity scatterplots, which summarize the enriched GO terms by removing term redundancy. The p-values for the enriched terms are shown by color, and the number of terms each circle represents is shown by its size.

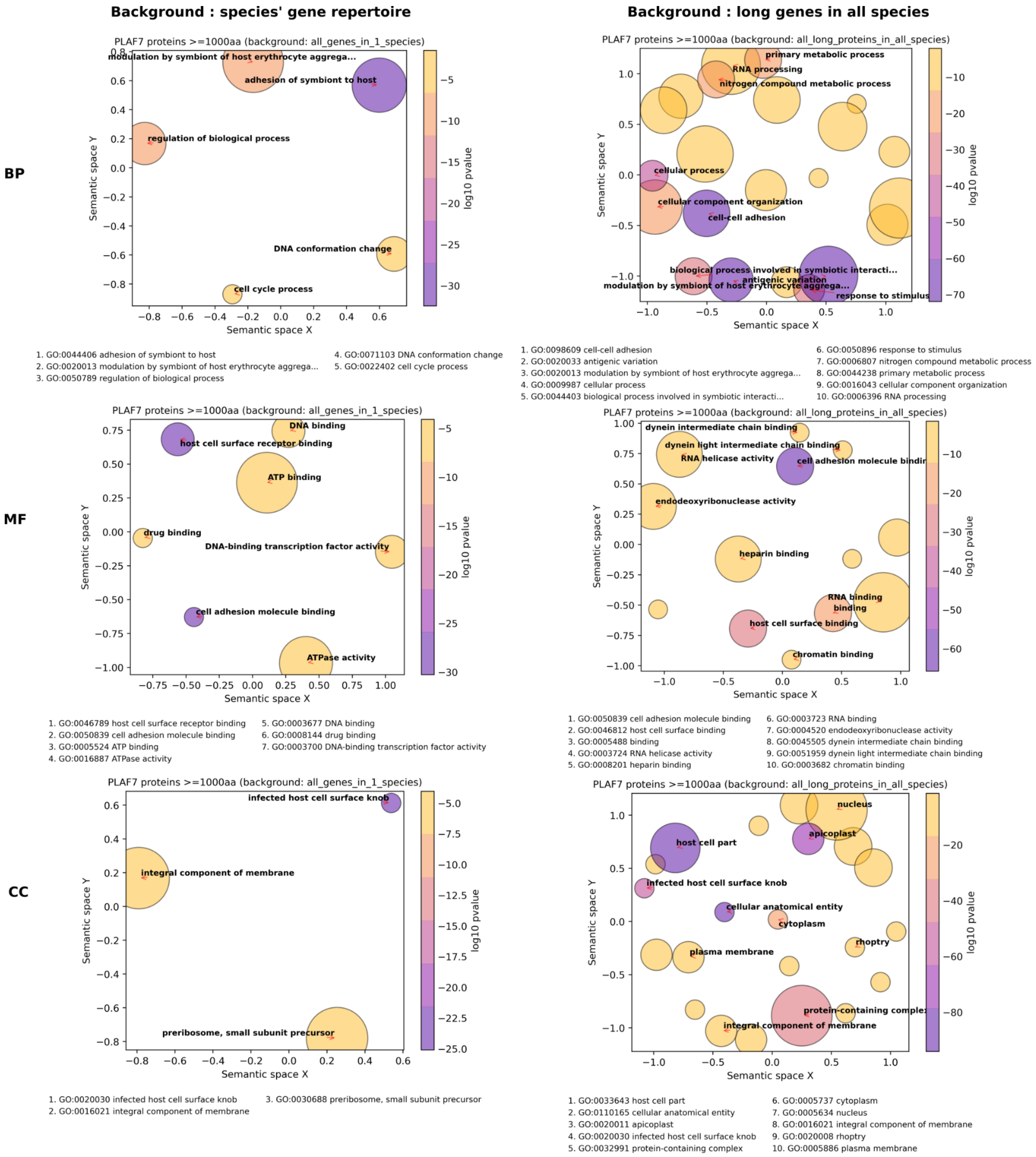

Supplement: Supplementary file 2 — Additional file 2. Supplementary Materials and Results. This file include all Supplementary Results: alternative distribution comparisons using Jensen-Shannon distance and standardized distribution; analysis of correlations between genomic features; analysis of “dubious” proteins contributing to atypical distribution and its support; comparisons of outliers proteomes with other annotation sets and functional analysis of proteomes with abundance of long proteins. It also includes all Supplementary Figures and Supplementary Table S2 [72–82]. [file 13059_2023_2973_MOESM2_ESM.pdf]
