# Supplementary material for: Protein length distribution is remarkably uniform across the tree of life
Source: Genome Biol. 2023 Jun 8;24:135. doi: 10.1186/s13059-023-02973-2 (PMC10251718; doi:10.1186/s13059-023-02973-2)
Supplement: Supplementary file 8 — Additional file 8. Review history. [file 13059_2023_2973_MOESM8_ESM.docx]

Review History

**First round of review**

**Reviewer 1**

**Were you able to assess all statistics in the manuscript, including the appropriateness of statistical tests used?**

Yes, and I have assessed the statistics in my report.

**Comments to author:**

Genome Biology review for the manuscript by Nevers, Gloverm, Dessimoz and Lecompte.

In this manuscript, the authors analyze the distribution of protein lengths across the tree of life. I think this is an important and interesting topic, and is highly relevant for journals such as Genome Biology.

Comments:

(1)	In the results, the authors compare averages and standard deviations of protein lengths among various groups (e.g., bacteria and archaea). The authors should correct for the phylogenetic signal. This issue has to be addressed across the entire work. It might be possible to develop a model in which a parameter, which quantifies the protein length, evolves along the underlying tree. Then one should test whether a model which assumes that all parts of the tree evolve with the same model parameters is inferior to a model that allows some parts of the tree to have different parameters. I think that such a modeling approach, if pursued, will substantially strengthen this work, as it will place it within a statistically robust framework for analyzing evolutionary changes. Without it, some parts of the manuscript are descriptive in nature.

(2)	Similarly, on page 5 the authors write that larger proteins show more variation than smaller ones. I think this is expected, assuming that indels have more chances to occur in longer sequences. In other words, even if both short and long proteins evolve under an identical indel process, the longer sequence will have higher standard deviation of length, as there are more positions where indels can occur. Again, a model with parameters and robust statistical tests is needed to test whether indel dynamics is different between long and short proteins.

(3)	The authors use some arbitrary cutoffs (e.g., 500 amino acids, page 5 or KS<0.2 on page 8). Why were these cutoffs chosen? Can the authors avoid using arbitrary cutoffs?

(4)	Page 6, "Even among eukaryotes, deuterosteomes .. diverge highly from all other species". Can you quantify how much they diverge? Is this statistically significant?

(5)	Page 8, GC content is explained here for the first time, but it was mentioned earlier in the manuscript (I am not sure it needs an explanation).

(6)	Page 8, proteomes were compared against species of their respective domains. How do you define domain? Same order? Family? Kingdom? What is the statistical test here? Do you correct for multiple testing?

(7)	Page 10, the authors report an association between species with atypical protein lengths and BUSCO score, but here too, they neither quantify the association nor test if the association is statistically significant.

(8)	Page 10, which species have a BUSCO score of less than 90? Please provide the information.

(9)	To better verify that species with atypical protein lengths indeed reflect annotation artefact, I suggest you analyze a small sample of short proteins and test for evidence that they are artefacts. For example, in Drosophila simulans, one can search for supporting expression data or proteomic data.

(10)	If indeed genomes that show atypical protein length distribution indicate annotation problems, could the authors try to characterize these problems? What is the source of these errors? Which proteins are erroneously annotated? Can one fix this? A deeper analysis is needed here.

(11)	In the discussion, it is claimed that longer proteins imply increased costs. It is not clear whether longer proteins with more functions also imply increased costs. I guess that an increased cost is only relevant if the protein does not gain a novel function. Maybe there is no strong selection against long proteins, but it is just not beneficial, i.e., most biological functions are executed with domains that are relatively short. This issue should be better discussed.

(12)	One can test features of long versus short proteins, e.g., their expression, codon bias (in organisms in which it is relevant), and Ka/Ks. This may help clarify the selective forces dictating protein lengths.

(13)	Discussion: the authors imply that a gamma distribution better fits protein lengths compared to exponential distribution. I did not find the statistical test for it.

(14)	Page 13, Isoelectric point - please provide the name of the Biopython package.

(15)	For the discussion: is it possible that when there is an atypical length distribution, Busco is wrong?

Text issues:

(1)	Line spacing is not uniform across the manuscript.

(2)	Sometimes there is no space before the reference (e.g., "proteins[4]").

(3)	The word "data" is sometimes used as plural and sometimes as singular.

(4)	Page 15, duplication of text (lines 16-17 and lines 43-44).

(5)	The references are not properly formatted.

**Reviewer 2**

**Were you able to assess all statistics in the manuscript, including the appropriateness of statistical tests used?**

Yes, and I have assessed the statistics in my report.

**Comments to author:**

Nevers et al. turn their attention to the phenomenon of a remarkable stability of the protein length distribution across the tree of life. While the fact itself is well-known in the field of comparative genomics, the authors take it a step further, providing a formal assessment of the distribution similarity and the comparisons with other genomic traits and characters. From my perspective this provides sufficient novelty to warrant a publication at Genome Biology.

I don't have any major concerns for the design and execution of this work; here are several comments that I'd like the authors to address:

- could you comment on the choice of the Kolmogorov-Smirnov statistic to compare the distributions? Typically it is used to provide a probabilistic answer on the questions "is it likely that the two observed distribution are randomly sampled from the same population?". The advantage of KSS is its scaling with the dataset size in a predictable manner (ultimately derived from the theory of extreme values distribution), but is it good for assessing the differences between distributions that are distinct a priori (like the distribution of GC content values in genes of different organisms)? There are many measures for distribution comparisons that provide a "smooth" integrated difference/similarity across the whole range of values rather than a "point-based" estimate (KSS=max(abs(F1(x)-F2(x)))) that seem to be a better fit for the question at hand.

- for distributions that span orders of magnitude (short peptides to titin) it is usually a good practice to show (and often analyze) them in a log scale. At the very least, protein length and gene length distributions on Fig. 1 and Fig. 3 would look much more informative in a semi-log plot. Possibly, comparing the distributions of log-length values might be beneficial for the authors' points too.

- I wonder if additional insights can be gleaned from comparison of standardized distributions (normalized to the mean of 0 and the standard deviation of 1). This comparison would focus on the shapes of the distribution ignoring the scale.

**Reviewer 3**

**Were you able to assess all statistics in the manuscript, including the appropriateness of statistical tests used?**

Yes, and I have assessed the statistics in my report.

**Comments to author:**

The manuscript of Nevers et al. explores the distribution of protein length across cellular life. The manuscript is short and straightforward but involves significant computations. it evaluates a number of interesting measures (scalar features or distributions) of proteomes (length, number of protein domains, isoelectric point, gene content, etc) retrieved from 2,326 species spanning the three domains of life.

Some suggestions:

(1) The main conclusion stressed by the authors is that length distributions are 'remarkably consistent' (in title and throughout the manuscript). However, consistency is a relative term often used to negate logical contradiction or state that data matches some pattern. I think the term is used by the authors to describe variation in length distributions across species being low. Perhaps consider revisiting the title.

(1) Wang et al. (2011)(PMID: 21730144) evaluated protein length within the context of domains and linkers. This work suggests an important role of reductive evolution in Bacteria and Archaea (but also in organisms engaging in parasitism, such as some organismal exceptions listed by the authors). Some additional work from the same group provides insight into how length relates to the combination of domains (PMID: 19141283), folding speed (PMID: 23341762) and economies of scales (PMID: 26260760). All of these papers merit citation and perhaps discussion relative to the findings being reported. Perhaps some of this research can also help elaborate some evolutionary explanations for the findings of this manuscript.

(2) Figure 1 is very informative and shows that protein and gene length distributions are heavily tailed, especially for Eukarya. This is expected given that the evolutionary rise of Eukarya resulted in significant levels of combination of domains in proteins relative to those of the microbial domains. Unfortunately, statistical measures of central tendency will fail to adequately capture these heavy-tailed patterns. A number of studies since the early 2000s revealed that power law distributions exist in domains and other features of proteins. Have the authors modeled their distributions to determine if the heavy tails arise from scale free patterns associated with length?

(3) The most extreme heavy tails are associated with scale-free distributions, which are also associated with economies of scales. The Menzerath-Altmann law of language for instance, which applies to protein length and protein domain content (e.g. PMID: 26260760 mentioned before) is one such example. But also check Friar et al. (2012)(PMID: 22629319) and how genome size is linked to the Benford distribution in its different regimes.

(4) In the discussion section (and abstract), 'purifying selection' is mentioned as the driving force that 'keeps a high proportion of the coding sequences between 50 to 500 amino acids'. However, the authors do not test this hypothesis or provide data in any way or form to give it such a central role. In absence of experimental or computational tests of purifying selection, their conclusions needs to be carefully reevaluated.

Purifying selection usually manifests at population levels. Instead, protein length materializes in evolution at much longer time frames, often billions of years of evolution. Other forces behind the typical Darwinian views exist, including forces of novelty generation and recruitment that may be more important. If purifying selection would be so central, then the chances of multi-domain proteins appearing or being recruited would be low, which is not the case given their popularity throughout life, but especially in Eukarya. In addition, there may be biophysical limitations related to the structure of proteins that may be more important or even interpretations related to information transmission that would be more relevant (as proposed in PMID: 22629319). The ''second factor (line 15, page 12) listed in discussion may be more relevant than the concept of purifying selection, given that organisms have to balance economy of resources. Again, consider the existence of strong reductive evolutionary forces related to microbial life (subjected to limitations of low Reynold numbers) being responsible for the dichotomy between prokaryotes and eukaryotes.

**Authors Response**

**Point-by-point responses to the reviewers’ comments:**

As you will see from the reports, the referees are broadly favorable and find the work of potential interest, but they raise important issues that we must ask you to address, in the form of a revised manuscript, before we reach a final decision on publication. In particular, it seems to us to be essential that Referee 1's concerns about correcting for the phylogenetic signal and the bias in variation due to protein length are resolved. Please ensure that these and all other issues raised by the referees are addressed in full.

AUTHOR RESPONSE: We thank the editor for the opportunity to address the feedback and submit a revised version of the manuscript. We worked to address the issues raised by the reviewer in this version of our work.

Reviewer #1:
Genome Biology review for the manuscript by Nevers, Glover, Dessimoz and Lecompte.

In this manuscript, the authors analyze the distribution of protein lengths across the tree of life. I think this is an important and interesting topic, and is highly relevant for journals such as Genome Biology.

*AUTHOR RESPONSE: We thank Reviewer #1 for their positive assessment.*

Comments:
REVIEWER COMMENT #1: In the results, the authors compare averages and standard deviations of protein lengths among various groups (e.g., bacteria and archaea). The authors should correct for the phylogenetic signal. This issue has to be addressed across the entire work. It might be possible to develop a model in which a parameter, which quantifies the protein length, evolves along the underlying tree. Then one should test whether a model which assumes that all parts of the tree evolve with the same model parameters is inferior to a model that allows some parts of the tree to have different parameters. I think that such a modeling approach, if pursued, will substantially strengthen this work, as it will place it within a statistically robust framework for analyzing evolutionary changes. Without it, some parts of the manuscript are descriptive in nature.

*AUTHOR RESPONSE: The reviewer's comment addresses the fact that comparing species as a whole within taxonomic groups would be impacted by the degree of relatedness of these species and bias our analysis. This is a legitimate concern, although this should not alter the main conclusion of this manuscript (that protein length distribution of length changes less in amplitude and across clades than other genomic features) since this bias affects all other genomic features as well. However, we agree that modeling the evolution of the different genomic parameters would allow us to have a better understanding of evolution of genomic features across the domain of life and we thank the reviewer for their suggestion.

Such an experiment requires obtaining a well resolved phylogenetic tree of the species considered. Since constructing a phylogenetic tree at the level of Life is difficult in and of itself,* *we chose to use a previously published species tree (from Hug et al, 2016). We semi-automatically mapped the species of our original dataset to this tree and carried out our analysis on the 603 species that could be placed on the species tree. This mapping is reported in Supplementary Table 3.

We implemented a modeling approach in which our different genomic features evolved along this species tree and in all cases test whether the evolution was best represented by Brownian or Ornstein–Uhlenbeck models of evolutions with a) a same set of parameters across species, b) a different set of parameters in Eukaryotes than in other species, or c) a different set of parameters across all domains of life. We now report these additional analyses in the main text, as they provide a more robust assessment of the different trends in evolution of genomic features as well as contextualization of our previous results. The results clearly show that protein length evolves under a higher rate in Eukaryotes, a trend that is also observed in terms of gene length, genome size and proteome size but not for GC content and isoelectric point evolution. This corroborates our previous conclusion that length-associated features follow a similar trend in terms of variation across Domains of life, although protein length varies relatively much less.

We now report the results in the manuscript in:
Page 6, l.121 - The second paragraph of the “Protein length distribution in the three Domains” section (“Based on this observation,[...] the statistical descriptor.”)
-Page 11, l. 234 - The sixth paragraph of the “Protein length is more uniform across species than other genomic features” section. (“Thus, protein number,[...] confirming a distinct pattern in this group of species.”
-Page 14, l. 285 - The last paragraph of the “Protein length is more uniform across species than other genomic features” section (“The different trends[...] (Supplementary Table 4).”)

Details of the results are also available in Supplementary Table 4 . Finally we added paragraphs about this experiment in the Methods section of the paper (“Modeling the evolution of genomic features”).

We also revisited a section of our Supplementary Results where we tested for correlation of genomic features, as these were also likely affected by phylogenetic autocorrelation (Subsection: “Correlations between genomic features”). To do this, and using the same species tree, we computed the Phylogenetic Independent Contrasts for numeric values representing the different characters studied in the manuscript and complemented our previous results accordingly.*


REVIEWER COMMENT #2 Similarly, on page 5 the authors write that larger proteins show more variation than smaller ones. I think this is expected, assuming that indels have more chances to occur in longer sequences. In other words, even if both short and long proteins evolve under an identical indel process, the longer sequence will have higher standard deviation of length, as there are more positions where indels can occur. Again, a model with parameters and robust statistical tests is needed to test whether indel dynamics is different between long and short proteins.

*We write that there is higher variation in the third quartile of protein length distribution than when considering median, which we believe is due to the tails of the distribution being wider in certain species. There may, indeed, be higher variation between homologs of larger proteins in comparison to small proteins due to indels hower we believe it is difficult to attribute variations in the tail of distributions of protein length to this alone, as the sets of proteins in any species are not all homolguous and in fact the number of proteins varies much as well. As we are not considering length variation between homologous sequences in this analysis, which would be difficult to model while staying in the scope of the paper.*

REVIEWER COMMENT #3. The authors use some arbitrary cutoffs (e.g., 500 amino acids, page 5 or KS<0.2 on page 8). Why were these cutoffs chosen? Can the authors avoid using arbitrary cutoffs?

*AUTHOR RESPONSE: The cut-off of 500 amino acids (page 5) is meant as a visual cue to describe Figure 1 and indicate that typical eukaryotic distributions tend to have long proteins past these thresholds whereas prokaryotic distributions do not. This threshold is mainly descriptive and has no impact over further analyses. However, for the distribution selected in Figure 1, we performed empirical pairwise comparisons of the eukaryotes distribution against other species. Over all pairwise comparisons, the maximal protein length after which there was a higher proportion of proteins in the Eukaryotic genome was 476 (see supporting Table 1 below). Thus, our estimate of 500 was permissive. We reformulated the paragraph to make our meaning more clear:

Page 7, l. 145 - “Eukaryotes tend to have a higher number of long proteins (>500 aa) than bacteria and archaea” -> “In our representative proteomes, eukaryotes consistently have a higher proportion of long proteins longer than 475 aa than bacteria and archaea”*

*For the second case (KS<0.2 on page 8), we now use the Tukey fence method to select outliers in terms of average KS divergence compared to their domain. This method is a well established method for identifying outliers. The cut-off is still 0.2 for Eukaryotes but is now 0.12 for Archaea and 0.09 for Bacteria. This leads to more species being selected as outliers for Bacteria and Archaea.
We added a new sentence to describe this:

Page 28, l. 604 - “For each Domain, we identified outlier proteomes in terms of divergence by using the Tukey’s fences method. Outliers were defined as proteomes with an average KS dissimilarity with the species of their respective Domains of more than 0.2 for eukaryotes, 0.12 for bacteria and 0.08 for archaea (Supplementary Table 5).”

This new way to compute outliers changes the numbers reported in the section “Many protein length distribution outliers are explained by quality issues” as well as Figure 4.*

REVIEWER COMMENT #4 Page 6, "Even among eukaryotes, deuterostomes .. diverge highly from all other species". Can you quantify how much they diverge? Is this statistically significant?

*AUTHOR RESPONSE: We added quantification of the divergence in Deuterostomes. We now report the average KS to other Eukaryotes, versus the average KS of other Eukaryotes between themselves, that is twice as large. We also report the results of an ANOSIM statistical test performed over the KS dissimilarity matrix (r stat = 0.89, p<0.001). The results show highly significant support for grouping Deuterostomes and other Eukaryotes as two separate groups.

As done to answer the reviewer’s first point above, we also tested whether an evolutionary model with a shift of gene length in the deuterostome was better supported than a common model over Eukaryotes. The model with an additional set of parameters in Deuterostomes was better supported than one with a common parameter across Eukaryotes, meaning this divergence is not necessarily due to a different regime of evolution in Deuterostomes. We now report all of these results in the main text.

We added:
-Page 10, l. 228 - “(average KS between deuterostomes and other eukaryotes: 0.48, mean KS between non-deuterostomia eukaryotes: 0.21; ANOSIM R: 0.89, p-value: 0.001)”
and
-Page 11, l. 244 - “Finally, for gene length, we tested a model with an additional set of parameters for deuterostomes only, because of the aforementioned higher divergence. This model was better supported than even the ones with distinct parameters for each Domain, confirming a distinct pattern in this group of species.”*

REVIEWER COMMENT #5 Page 8, GC content is explained here for the first time, but it was mentioned earlier in the manuscript (I am not sure it needs an explanation).

*AUTHOR RESPONSE: We removed the unnecessary explanation on page 8.*

REVIEWER COMMENT #6 Page 8, proteomes were compared against species of their respective domains. How do you define domain? Same order? Family? Kingdom? What is the statistical test here? Do you correct for multiple testing?

*AUTHOR RESPONSE: Taxonomically, Domains are generally understood as the superkingdom level (Archaea, Bacteria, Eukaryotes) since https://www.ncbi.nlm.nih.gov/pmc/articles/PMC54159/. We added a definition at the first mention of Domains and chose to capitalize it through the manuscript to avoid confusion with “protein domains”. We modified the text to say:*

*Page 4, line 76 - “A second study confirmed the divergence between eukaryotic and prokaryotic protein length, and noted that protein length distribution was generally uniform within Domains of Life (bacteria, archaea and eukaryotes).”

The comparisons we are describing there are all species pairwise comparisons between genomic features. In this case, the numbers we report are the average KS or inverted ratio between species of the same domain or between species of different domains. The values we report are essentially descriptive and were meant to describe the range of variation of parameters and distribution we observed between species. We now added an ANOSIM test to assess whether the domains are significantly dissimilar to one another. As pointed out by the reviewer, these analyses did not take into account the phylogenetic relationship between species. However, this relationship was taken into account in the analyses we used to address reviewer comment #1, and we concluded for most comparisons that there are different evolutionary regimes in the three Domains.

We added an introduction to ANOSIM tests at the start of the first paragraph of the “Protein length is more uniform across species than other genomic features” section:

Page 9, l.177 - “Results of all pairwise comparisons were used to generate symmetric dissimilarity matrices. On these matrices, we performed Analysis of Similarities (ANOSIM) [...] than within groups.”

We systematically report whether there is significantly more similarity within domains than between domains in the section. Given that we did not perform many simultaneous testing, we did not correct for multiple testing.*

REVIEWER COMMENT #7 Page 10, the authors report an association between species with atypical protein lengths and BUSCO score, but here too, they neither quantify the association nor test if the association is statistically significant.

*AUTHOR RESPONSE: Our claim in this section is that the proteomes with outlier distributions are enriched in incomplete proteomes. We provided a comparative analysis of the number of incomplete genomes in the “outlier” dataset and the complete dataset, where we show that proteomes with low BUSCO score are more represented in the outlier dataset. To clarify the quantification, we now report the fold enrichment and provide the results of a one-sided Fisher’s exact test that shows the enrichment is significant.

Page 17, l. 334 - “ In particular, proteomes with an atypical distribution are enriched in genomes from the incomplete category: 28 of 37 eukaryotic proteomes (75%, 1.6 fold enrichment, Fisher one-sided exact test p-value: 3.3e-4), 6 of the 15 archaeal proteomes (40%, 3.6-fold enrichment, Fisher one-sided exact test p-value: 2.0e-3), and 26 of the 122 bacterial proteomes (21%, 2.7-fold enrichment, Fisher one-sided exact test p-value:1.1e-6), meaning that in these cases, low quality genomes may be a cause of the atypical distribution. “*

REVIEWER COMMENT #8 Page 10, which species have a BUSCO score of less than 90? Please provide the information.

*AUTHOR RESPONSE: We now include the categories of proteomes (less or more than 90% complete BUSCO) and the binary classification of whether a proteome is an outlier in Supplementary Table 5, with a label for all genomes with a lower than 90% BUSCO Completeness. We reference this table in the manuscript where appropriated.*

REVIEWER COMMENT #9 To better verify that species with atypical protein lengths indeed reflect annotation artefact, I suggest you analyze a small sample of short proteins and test for evidence that they are artefacts. For example, in Drosophila simulans, one can search for supporting expression data or proteomic data.

*AUTHOR RESPONSE: We performed additional analyses to verify whether the shortest proteins in the Drosophila simulans are likely artefactual.
In short, we compared our version of the Drosophila simulans proteome (which is skewed toward shorter proteins compared to other Drosophilae proteomes) to the current version of the RefSeq proteome for the same species (which has a length distribution closer to other Drosophilae). The short proteins that make up the difference between the two versions of the genomes 1) have less transcriptomic support, 2) are richer in annotated ‘disordered regions’, 3) have less known orthologs, and 4) have poorly resolved protein structures as predicted by AlphaFold than the ones of the same length present in both annotations. These four additional lines of evidence, in addition to our previous observation that atypical protein length distribution is never seen in model species and varies between annotation versions, corroborate the notion that most of these short proteins are likely annotation artifacts.

This analysis has been added as a supplementary analysis of the paper (Section: “Atypical protein length is attributable to weakly supported protein sequence”). We now mention it in the main text as well:

Page 15, l. 304 - “Interestingly, a most recent annotation of Drosophila melanogaster has a protein length distribution much closer to Drosophila simulans and deprecated proteins - which tend to be small proteins - have low transcriptomic support and are predicted as disordered (see Supplementary Results).”

and in the Discussion section - page 21, l. 410 - “In this manuscript, we observed a similar trend for the most recent annotation of Drosophila simulans, with now deprecated– and likely erroneous– sequences leading to overestimation of small proteins.”*

REVIEWER COMMENT #10 If indeed genomes that show atypical protein length distribution indicate annotation problems, could the authors try to characterize these problems? What is the source of these errors? Which proteins are erroneously annotated? Can one fix this? A deeper analysis is needed here.

*AUTHOR RESPONSE: We agree a more in-depth analysis would be needed to fully characterize this issue. However, this is made complicated by the fact that the genomic data we used can not be easily linked to assembly data. Additionally, data about the annotation process of the genome is often not made easily available with the public data which further complicates the task. Because of this, we believe describing the issue more thoroughly falls in the realm of future work. Nevertheless, we currently provide circumstantial evidence that this is either an annotation artifact or related to genome assembly fragmentation. In particular, the observation that this is not occurring in well curated species but does happen in some of their closely related species and* *that atypical distributions are not always observed in other annotation sets for the same species tend to support this hypothesis.*

REVIEWER COMMENT #11 In the discussion, it is claimed that longer proteins imply increased costs. It is not clear whether longer proteins with more functions also imply increased costs. I guess that an increased cost is only relevant if the protein does not gain a novel function. Maybe there is no strong selection against long proteins, but it is just not beneficial, i.e., most biological functions are executed with domains that are relatively short. This issue should be better discussed.

*AUTHOR RESPONSE: We thank the reviewer for their comment which pointed out that this point was not correctly addressed before. We reworked thoroughly the second and third paragraphs from the last section of the discussion to address this and the points raised by reviewer 3.*

REVIEWER COMMENT #12 One can test features of long versus short proteins, e.g., their expression, codon bias (in organisms in which it is relevant), and Ka/Ks. This may help clarify the selective forces dictating protein lengths.

*AUTHOR RESPONSE: Thank you for the suggestions. We agree these analyses would be interesting, but considering the substantial additional work they would require, we chose not to expand the scope of our investigations but rather prioritize additional controls on our existing claims. We have however added these future directions as part of the last paragraph of the discussion section:

Page 22, l.460 - “Future investigations of the evolutionary forces acting on protein length would necessitate measuring how the size variation of orthologous sequences vary between species and whether it is an agreement with the overall trend of protein length distribution. Other insights could be gleaned by studying how proteins of different lengths vary in terms of evolutionary rates or differential expression to better characterize how the way they evolve dictates change in overall distributions. For instance, recent studies have shown that housekeeping genes tend to be shorter than other transiently expressed genes [50], making them easier to regulate than longer genes.”*

REVIEWER COMMENT #13 Discussion: the authors imply that a gamma distribution better fits protein lengths compared to exponential distribution. I did not find the statistical test for it.

*AUTHOR RESPONSE: This implication was derived from previous work describing protein length distribution but formulated in a way that could be interpreted as an observation in our work. However, we agree this was not clear. We changed the formulation and added the relevant citations:*

*Page 22, l. 445 - “the observed gamma distribution” -> “species, has been described as a gamma distribution or a log-normal distribution with a long tail fitting of a power-law distribution in the last percentile (rather than a decreasing exponential shape) [13,14,17].”*

REVIEWER COMMENT #14 Page 13, Isoelectric point - please provide the name of the Biopython package.

*AUTHOR RESPONSE: Page 24, l.508 - We changed the sentence to “using the Protparam module within the SeqUtils package from Biopython.”*

REVIEWER COMMENT #15 For the discussion: is it possible that when there is an atypical length distribution, Busco is wrong?

*AUTHOR RESPONSE: We added a few sentences addressing this point. We wish to point out that BUSCO is composed of gene families conserved in most species of their lineage, and that for a protein to be considered as fragment, it must deviate from the mean size by more than twice the standard deviation of the gene family. While it is believable that such proteins exist, it is unlikely that many proteins be in this case while retaining their conserved function. Their existence being a result of artifactual annotation appears much more plausible.

Page 20, l. 397 - fourth paragraph of the discussion “according to BUSCO. [...] genomic assembly or annotation artifacts.”*

REVIEWER COMMENT #16 Text issues:
(1) Line spacing is not uniform across the manuscript.
(2) Sometimes there is no space before the reference (e.g., "proteins[4]").
(3) The word "data" is sometimes used as plural and sometimes as singular.
(4) Page 15, duplication of text (lines 16-17 and lines 43-44).
(5) The references are not properly formatted.

*AUTHOR RESPONSE: Thank you for pointing out these oversights. We uniformized line spacing and fixed the reference formatting to be inline with the expected format for Genome biology. We added spacing before the references. We now always use data as plural.

Registered change:
Page 22, l. 450 - “as our data suggests” -> “as our data suggest”
Page 23, l. 482 - “This data”->”Genome length data”*

*The duplicated text “A curated version of the code is available as an independent Jupyter Notebook (Supplementary Data).” was referring to two different Notebooks for each sub-analysis. Since this is already mentioned in “Data availability” however, we decided to remove it from the Method section.*

**Reviewer #2**
Nevers et al. turn their attention to the phenomenon of a remarkable stability of the protein length distribution across the tree of life. While the fact itself is well-known in the field of comparative genomics, the authors take it a step further, providing a formal assessment of the distribution similarity and the comparisons with other genomic traits and characters. From my perspective this provides sufficient novelty to warrant a publication at Genome Biology.

*AUTHOR RESPONSE: We thank Reviewer #2 for their positive assessment.*

I don't have any major concerns for the design and execution of this work; here are several comments that I'd like the authors to address:

REVIEWER COMMENT #1 - could you comment on the choice of the Kolmogorov-Smirnov statistic to compare the distributions? Typically it is used to provide a probabilistic answer on the questions "is it likely that the two observed distribution are randomly sampled from the same population?". The advantage of KSS is its scaling with the dataset size in a predictable manner (ultimately derived from the theory of extreme values distribution), but is it good for assessing the differences between distributions that are distinct a priori (like the distribution of GC content values in genes of different organisms)? There are many measures for distribution comparisons that provide a "smooth" integrated difference/similarity across the whole range of values rather than a "point-based" estimate (KSS=max(abs(F1(x)-F2(x)))) that seem to be a better fit for the question at hand.

*AUTHOR RESPONSE: We chose the Kolmogorov-Smirnov statistics as a measure of similarity between distributions since it allowed to obtain a fast measure of distribution dissimilarity that integrated both shape and location across discrete (gene length, protein length) and continuous (GC, isoelectric) distributions while avoiding transforming the data (for example, to infer a continuous distribution). We recognize smoother methods exist for this purpose and now provide a similar analysis using Jensen-Shannon divergence in the updated paper. Reassuringly, the results we obtained with JS divergence are highly similar to the one we obtained with KSS. We now include this additional analysis as a Supplementary Analysis.

Page 8, l. 169 - We added a paragraph when first describing KS statistics and referring to this analisis:
“KS is a point estimate of the largest divide between two cumulative probability distributions, thus it can account for variation in both shape and location of the distribution and does not need to estimate continuous distributions from discrete data. However, since it is only a point measurement it may not accurately reflect subtle differences in distribution. We also performed a comparison using the Jensen-Shannon distance. All values below are reported in KS, but equivalent Jensen-Shannon distances are reported in Supplementary Results and support similar conclusions.”

The difficulty to use such methods for comparison distribution is that they are designed to compare continuous distributions, which must be estimated from the data. To perform this and allow for homogeneous comparison of the data, we estimated the kernel density according to the Epanechnikov function. As the number of proteins differ between genomes, using small windows for the density estimate led to overdiscretization of the data. In order to avoid this, we used the Freedman-diaconis criterion to select an appropriate window size for each dataset. This is detailed in the Supplementary methods in the “Divergence between distributions” section.

This measure of distribution dissimilarity is still sensitive to densities not overlapping like is the case with GC content, but we believe the fact that the variation can be this extreme for this genomic feature is still noteworthy. In response to a later point, we also performed a comparison* *of standardized distribution, where GC content also appears to be more variable than protein length in shape.*

REVIEWER COMMENT #2- for distributions that span orders of magnitude (short peptides to titin) it is usually a good practice to show (and often analyze) them in a log scale. At the very least, protein length and gene length distributions on Fig. 1 and Fig. 3 would look much more informative in a semi-log plot. Possibly, comparing the distributions of log-length values might be beneficial for the authors' points too.

*AUTHOR RESPONSE: We thank the reviewer for their helpful suggestion. We redid Fig 1 and Fig 3 using log-value of protein and gene length distribution. We agree using the logarithm of gene length distribution leads to a more informative plot as the spread of gene length across species is large - we changed Figure 1 in the main text accordingly. It is more debatable for protein length distribution, where it makes it harder to visualize change in the distribution of small proteins. Regardless, we now include the novel plots as Supplementary figure 1 (complementary to Figure 1) and Supplementary Figure 10 (complementary to Figure3). The existence of these alternative representations is also mentioned in the legend of said Figure.

We also compared the distribution of log-length value for protein and gene length. Note that the Kolmogrov-Smirnov statistic is identical whether we compare distribution of length or log-length, as the transformation does not change the ranking. However, we compared using Jensen-Shannon divergence, with similar results - This is now also included in the same Supplementary Analysis as mentioned in point 1.*

REVIEWER COMMENT #3- I wonder if additional insights can be gleaned from comparison of standardized distributions (normalized to the mean of 0 and the standard deviation of 1). This comparison would focus on the shapes of the distribution ignoring the scale.

*AUTHOR RESPONSE: We now added a similar analysis of standardized distribution, focused on similarity between shapes. The overall trends we observed in the main results for gene and protein length, as well as for isoelectric points are similar in the comparison of standardized distributions. However, they differ for GC content when the dissimilarity is low overall within and across Domains, in a trend that is similar to protein length distribution. Thus, the higher divergence of GC content distribution can be mainly explained by their variation in location. We report these results as an additional Supplementary material and in the Result section when comparing GC distributions:

Page 14, l.267 - “We note, however, that the high divergence between GC content distribution is mainly due to difference of location in distribution, leading them in the most extreme case to not overlap. When performing similar analysis over standardized GC distribution, the shape of distribution displays similar uniformity as protein length distribution across Life (Supplementary Data).”*

**Reviewer #3**
The manuscript of Nevers et al. explores the distribution of protein length across cellular life. The manuscript is short and straightforward but involves significant computations. it evaluates a number of interesting measures (scalar features or distributions) of proteomes (length, number of protein domains, isoelectric point, gene content, etc) retrieved from 2,326 species spanning the three domains of life.

*AUTHOR RESPONSE: We thank Reviewer #3 for their positive assessment.*

Some suggestions:

REVIEWER COMMENT #1 The main conclusion stressed by the authors is that length distributions are 'remarkably consistent' (in title and throughout the manuscript). However, consistency is a relative term often used to negate logical contradiction or state that data matches some pattern. I think the term is used by the authors to describe variation in length distributions across species being low. Perhaps consider revisiting the title.

*AUTHOR RESPONSE: Thank you for pointing this out. We changed the title to “Protein length distribution is remarkably uniform across Life”, which is more in line with what we mean. We also changed other sentences where “consistent” was used in the same way.*

REVIEWER COMMENT #2 Wang et al. (2011)(PMID: 21730144) evaluated protein length within the context of domains and linkers. This work suggests an important role of reductive evolution in Bacteria and Archaea (but also in organisms engaging in parasitism, such as some organismal exceptions listed by the authors). Some additional work from the same group provides insight into how length relates to the combination of domains (PMID: 19141283), folding speed (PMID: 23341762) and economies of scales (PMID: 26260760). All of these papers merit citation and perhaps discussion relative to the findings being reported. Perhaps some of this research can also help elaborate some evolutionary explanations for the findings of this manuscript.

*AUTHOR RESPONSE: We thank the reviewer for their helpful suggestions and bringing to their attention these papers. We reworked the second and third to last paragraphs of the discussion in our manuscript, using concepts introduced in these references, while adding citations where appropriate.*

REVIEWER COMMENT #3 Figure 1 is very informative and shows that protein and gene length distributions are heavily tailed, especially for Eukarya. This is expected given that the evolutionary rise of Eukarya resulted in significant levels of combination of domains in proteins relative to those of the microbial domains. Unfortunately, statistical measures of central tendency will fail to adequately capture these heavy-tailed patterns. A number of studies since the early 2000s revealed that power law distributions exist in domains and other features of proteins. Have the authors modeled their distributions to determine if the heavy tails arise from scale free patterns associated with length?

*AUTHOR RESPONSE: We thank the reviewer for their interesting points. As they mentioned, mathematically modeling the different distribution of protein length itself has been the focus of previous works. The aforementioned previous works, including some we were already citing,* *reported that the long tails of distributions were better fitted by a power-law distribution. We now mention it clearly in the introduction. However, rather than revisit the specific question of the shape of the length distribution, we chose to focus our study on the related but different question of how variable the distribution is across different clades of the tree of life, in particular compared to other characteristics of genomes/proteomes. Specifically, we added:

Page 4, l. 74 - “described as either a gamma or log-normal distribution with a long tail more fitting of a power-law distribution [14]”
Page 22, l. 445 - “with a long tail fitting of a power-law distribution in the last percentile (rather than a decreasing exponential shape) [13,14,17].”*

REVIEWER COMMENT #4 The most extreme heavy tails are associated with scale-free distributions, which are also associated with economies of scales. The Menzerath-Altmann law of language for instance, which applies to protein length and protein domain content (e.g. PMID: 26260760 mentioned before) is one such example. But also check Friar et al. (2012)(PMID: 22629319) and how genome size is linked to the Benford distribution in its different regimes.

*AUTHOR RESPONSE: We added an explicit mention of how the long tails of our distributions would be consistent with scale free distribution, especially the Menzerath-Altmann law of language as it applies to protein length. This is in the second-to-last chapter of the discussion:

Page 22, l.456 - “The relationship between protein length and domain organization in the context of resource economy and functional flexibility has been explored before and shown to follow the Menzerath-Altmann law of language [58].”*

REVIEWER COMMENT #5 In the discussion section (and abstract), 'purifying selection' is mentioned as the driving force that 'keeps a high proportion of the coding sequences between 50 to 500 amino acids'. However, the authors do not test this hypothesis or provide data in any way or form to give it such a central role. In absence of experimental or computational tests of purifying selection, their conclusions needs to be carefully reevaluated.

Purifying selection usually manifests at population levels. Instead, protein length materializes in evolution at much longer time frames, often billions of years of evolution. Other forces behind the typical Darwinian views exist, including forces of novelty generation and recruitment that may be more important. If purifying selection would be so central, then the chances of multi-domain proteins appearing or being recruited would be low, which is not the case given their popularity throughout life, but especially in Eukarya. In addition, there may be biophysical limitations related to the structure of proteins that may be more important or even interpretations related to information transmission that would be more relevant (as proposed in PMID: 22629319). The ''second factor (line 15, page 12) listed in discussion may be more relevant than the concept of purifying selection, given that organisms have to balance economy of resources. Again, consider the existence of strong reductive evolutionary forces related to microbial life (subjected to limitations of low Reynold numbers) being responsible for the dichotomy between prokaryotes and eukaryotes.

*AUTHOR RESPONSE: We thank the reviewer for this insightful comment. We removed mention of purifying selection, as it is not something we can easily test. Instead, and as mentioned before we reworked the discussion in order to take into account the literature mentioned in response the Reviewer 3 Comment #1 as well as the differential constraint between eukaryotes and other species likely related to constraint of microbial life.*

**Second round of review**

**Reviewer 1**

An excellent revision of the manuscript. I don't have any additional comments or suggesitons.

**Reviewer 2**

I am satisfied with the revision; my questions have been addressed.

**Reviewer 3**

Thank you for addressing the points that I have raised in the initial review. I also see the authors have addressed in satisfactory manner other interesting commentary and suggestions from the other two reviewers that examined this contribution.
